# Novel small molecule modulators of plant growth and development identified by high-content screening with plant pollen

**DOI:** 10.1186/s12870-016-0875-4

**Published:** 2016-09-06

**Authors:** Roman Chuprov–Netochin, Yaroslav Neskorodov, Elena Marusich, Yana Mishutkina, Polina Volynchuk, Sergey Leonov, Konstantin Skryabin, Andrey Ivashenko, Klaus Palme, Alisher Touraev

**Affiliations:** 1 Moscow Institute of Physics and Technology, Dolgoprudny, 141700 Moscow region Russian Federation; 2 Research Centerof Biotechnology of the Russian Academy of Science, 117312 Moscow, Russian Federation; 3 Lomonosov Moscow State University, 119991 Moscow, Russian Federation; 4 Faculty of Biology; BIOSS Centre for Biological Signaling Studies; ZBSA Centre for Biological Systems Analysis, University of Freiburg, Schänzlestr.1, 79104 Freiburg, Germany

**Keywords:** Chemical library, Pollen, Pollen tube, Growth, Growth regulator

## Abstract

**Background:**

Small synthetic molecules provide valuable tools to agricultural biotechnology to circumvent the need for genetic engineering and provide unique benefits to modulate plant growth and development.

**Results:**

We developed a method to explore molecular mechanisms of plant growth by high-throughput phenotypic screening of haploid populations of pollen cells. These cells rapidly germinate to develop pollen tubes. Compounds acting as growth inhibitors or stimulators of pollen tube growth are identified in a screen lasting not longer than 8 h high-lighting the potential broad applicability of this assay to prioritize chemicals for future mechanism focused investigations in plants. We identified 65 chemical compounds that influenced pollen development. We demonstrated the usefulness of the identified compounds as promotors or inhibitors of tobacco and Arabidopsis thaliana seed growth. When 7 days old seedlings were grown in the presence of these chemicals twenty two of these compounds caused a reduction in Arabidopsis root length in the range from 4.76 to 49.20 % when compared to controls grown in the absence of the chemicals. Two of the chemicals sharing structural homology with thiazolidines stimulated root growth and increased root length by 129.23 and 119.09 %, respectively. The pollen tube growth stimulating compound (S-02) belongs to benzazepin-type chemicals and increased Arabidopsis root length by 126.24 %.

**Conclusions:**

In this study we demonstrate the usefulness of plant pollen tube based assay for screening small chemical compound libraries for new biologically active compounds. The pollen tubes represent an ultra-rapid screening tool with which even large compound libraries can be analyzed in very short time intervals. The broadly applicable high-throughput protocol is suitable for automated phenotypic screening of germinating pollen resulting in combination with seed germination assays in identification of plant growth inhibitors and stimulators.

**Electronic supplementary material:**

The online version of this article (doi:10.1186/s12870-016-0875-4) contains supplementary material, which is available to authorized users.

## Background

The identification of novel physiologically active compounds via phenotypic screening of chemical libraries and their application in functional studies becomes increasingly relevant to plant biology [1–3]. In the majority of studies, either plant cell cultures, or whole seedlings are used in phenotype-based chemical screens [4] aimed at identifying small molecules, which target processes, such as cell wall biosynthesis [5], cytoskeleton functions [6], hormone biosynthesis [7] and signaling [5, 8], gravitropism [9], pathogenesis, purine biosynthesis and endomembrane trafficking [10–13]. In some cases, associated gene targets have been identified [14–20]_._ Despite of these achievements, further progress in plant chemical biology largely depends on to what extent image-based screening pipelines can be improved and applied to increase the spatio-temporal phenotypic resolution of fast growing plant systems, and enable the rapid and sensitive screening of large small molecule libraries [14, 21, 22]. Published screens are typically slow using seedlings germinated from seeds and grown in medium, containing the chemicals of interest [14].

Pollen grains have several unique features, which make them ideally suited to high-throughput chemical biology screens [21, 23]. Firstly, an ample supply of uniform pollen can be easily obtained from only a few flowering plants. Secondly, pollen germination and growth of pollen tubes are very rapid processes, which can be measured efficiently over time-scales of hours. Thirdly, the complete screening procedure can be performed under non-sterile condition on the laboratory bench. Finally, and most importantly, almost 70 % of all genes of the plant under study are transcribed in developing pollen [24]. We therefore hypothesize that any compound, found to inhibit or stimulate pollen germination and tube growth is likely to affect also other plant processes, such as seed germination, growth or differentiation of roots or shoots.

## Results

**Fig. 1:**
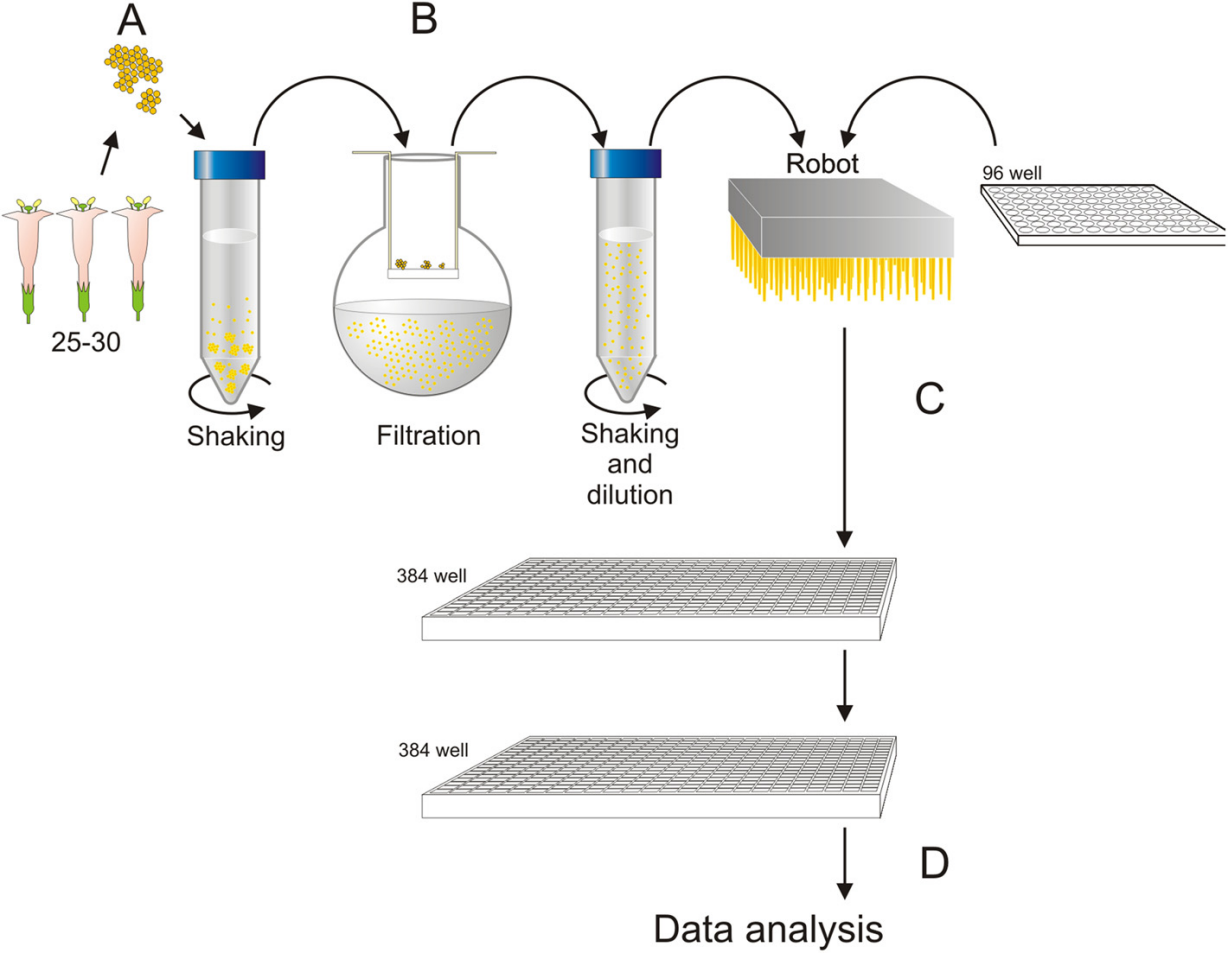
Work flow of high - throughput screening pollen assay. **a** Collecting of pollen grains. **b** Preparation of pollen suspension. **c** Preparation of assay. **d** Plate image acquisition and data analysis

**Fig. 2:**
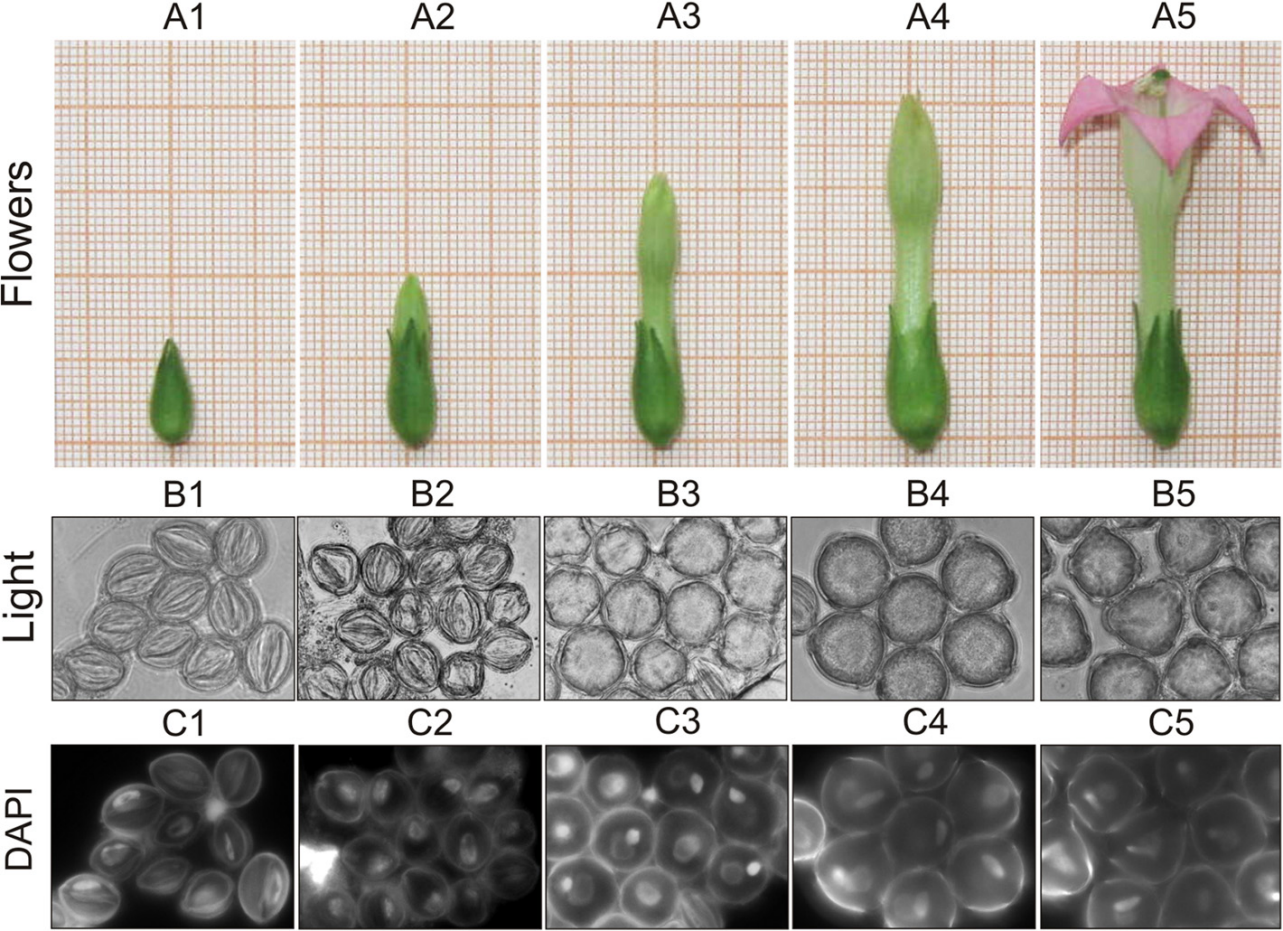
Correlation between flower bud size and the stage of pollen development in tobacco *Nicotianatabacum L*. plant. Microspores and pollen were isolated from buds of different sizes, stained with DAPI, and viewed under a fluorescence microscope using the UV light channel and normal light to determine the developmental stages of pollen. **a1-a5** Flowers of various sizes. **a1** 10-12 mm. **a2** 18-22 mm. **a3** 28–32 mm. **a4** 38–42 mm. **a5** open flower. **b1-b5** Pollen at different developmental stages visualized by light microscope. **c1-c5** Pollen at different developmental stages visualized by UV. **b1**and **c1** Unicellular microspores. **b2, c2** Early bicellular pollen. **b3, c3** Mid‐bicellular pollen. **b4, c4** Nearly mature pollen. **b5, c5** Fully mature pollen

Our high-throughput phenotypical screen integrates operational details of published small molecule screens in various plants and key considerations, when embarking on such a chemical screen. An overview of the strategy is shown in Fig. 1. In tobacco, floral bud size is a good indicator of pollen developmental stage [25]. In order to verify the applicability of this correlation, we analyzed 4′,6-diamidino-2-phenylindole (DAPI)-stained pollen taken from flower buds of different sizes. Results have shown that freshly opened flowers of approximately 40–45 mm in size, which contained fully mature pollen grains, were optimal for screening experiments (Fig. 2). For efficiency of the screen it was critical to identify the correct stage of pollen development for which tobacco pollen grains were collected by gentle stirring of freshly opened flowers into the Eppendorf tube. Then, pollen grains were re-suspended in liquid germination medium (GVH14) and incubated at room temperature. Germination of pollen took place after 30 min and growing pollen tubes could be analyzed after less than 120 min. Next, pollen culture conditions were optimized. The density and homogenous distribution of pollen grains were found to be the most important factor for obtaining reproducible results by image-based evaluation of pollen phenotypes. Most importantly, in order to obtain good images, pollen grains and their growing pollen tubes had to be well separated with little overlap and interference. We tested different densities ranging from 100 up to 10.000 pollen grains per ml medium and found that 4.000 pollen grains per ml were optimal to obtain a good discrimination of individual pollen tubes with meaningful estimation of tube length along with excellent statistical values (in terms of CV and Z’ factor).

**Fig. 3:**
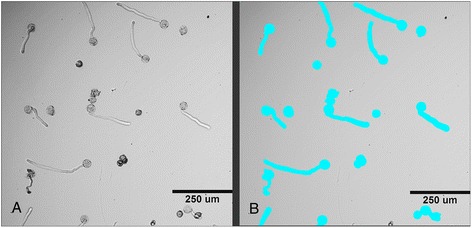
Example of the image, processed by the algorithm, based on the Custom Module Editor software to define the objects of interest. **a** The image of pollen suspension, cultured in the presence of one of tested chemical in 384-multi-well plate (transmitted light, 10x magnification, image was acquired at 120 min time-point of incubation. **b** Binary mask overlayed on the same image (**a**) after processing by algorithm to evaluate the total area of pollen suspension

For screening experiments, pollen was suspended in GVH14 medium, then transferred into 384 multi-well plates and compounds added to each well. A primary screen plate acquisition, using tobacco pollen germination and tube growth as phenotype features was started after 120 min of pollen incubation with test compounds at room temperature and completed in 8 h. For image analysis we developed an algorithm, which measures the total area, occupied with all visualized pollen grains in each well of multi-well plate (Fig. 3). The quantification of the total area, occupied by all visualized pollen grains, correlates well with inhibitory (total area decreases) or stimulatory (total area increases) effects of tested compounds.

Robustness and reproducibility of the assay was estimated by statistical analysis of the obtained numerical data, collected after a series of at least four identical experiments. Pollen in GVH14 medium containing 0.1 % dimethyl sulfoxide (DMSO) without added chemical compounds was used as a zero control, whereas pollen suspended in GVH14 media containing 0.1 % DMSO, 1 μM salicylic acid, an established inhibitor of pollen germination and tube growth, was used as negative control. For evaluation of assay performance different statistical parameters were used for quality and reproducibility assessment. We determined intra-plate and inter-plate variability, evaluating the data of maximum and minimum signals of control plates, which were set up in duplicates. We also applied Z’-factor analysis with Z-factors typically ranging in average to 0.45 in four independent experiments with +/− 5 % of variability between each experiments depending on different pollen populations chosen for each test. Statistical analysis confirmed reproducible high-throughput screening (HTS) data with standard deviation (SD) 0.45 ± 0.0276 indicating a good dynamic range and high reproducibility (Additional file 1).

Ideally, a chemical library should contain a range of diverse biologically active and structurally diverse chemicals. We first established a focused small chemical library sharing structural features with well-known plant growth inhibitors or activators from indole-, adenine-, β-carboline- and phytodisteroide families [5]. Chemicals were chosen using topological pharmacophore fingerprint, in which Tanimoto coefficient was calculated with respect to the compounds of the reference sample. The final picking included compounds with the highest ratio of topological similarity with respect to known inhibitors and activators of plant growth. In order to evaluate our assay specificity and possible hit rate, the focused small library was randomly incorporated into a panel of 940 compounds chosen *via* scaffold hopping and similarity of molecular weight, solubility and structure elements. The combined chemical library consisted of 1040 compounds (Additional file 2). Each compound was tested at 10 uM final concentration diluted from 10 mM stock solution dissolved in 100 % DMSO. Theprimary screening of 1040 compounds resulted in 65 potential hits (Additional file 3), which included inhibitors and stimulators of pollen tube growth.

**Fig. 4:**
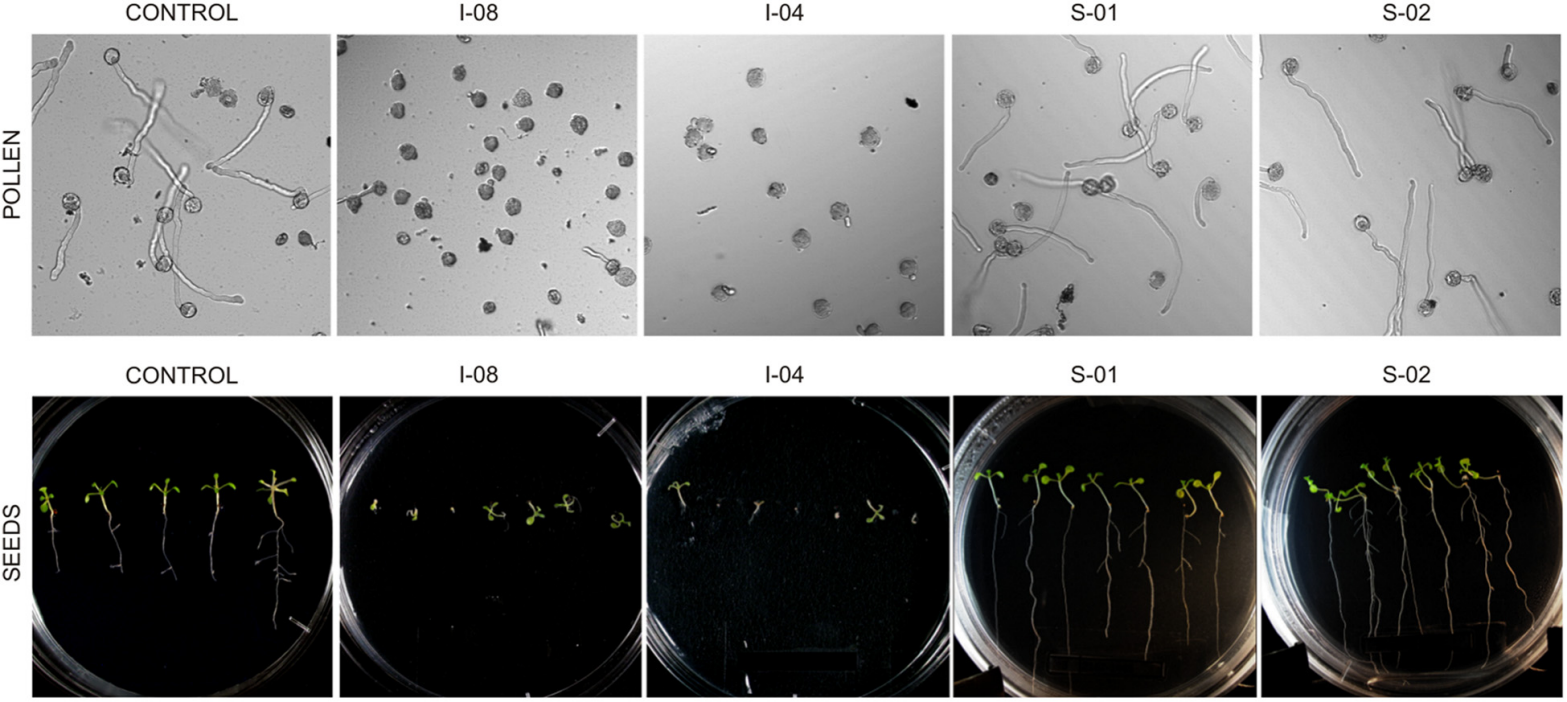
Effect of representative chemical compounds on pollen tube growth, seed germination and Arabidopsis root growth. Panel “**Pollen**”: Control: pollen tube growth in medium GVH14 without added chemicals; I-08: pollen tube growth in the presence of inhibitor I-08; I-04: pollen tube growth in the presence of inhibitor I-04; S-02: pollen tube growth in the presence of stimulator S-02; S-01: pollen tube growth in the presence of stimulator S-01. All chemicals were tested in germination medium GVH14 at concentrations of 100 μM. Pictures are taken after 120 min of incubation of pollen grains in corresponding media in one well of 384-well plate, transmitted light, 10x magnification. Panel “**Seeds**”: Control: Arabidopsis seeds germination in medium GVH14 without added chemicals; I-08: plant seeds germination in the presence of inhibitor I-08; I-04: plant seeds germination in the presence of inhibitor I-04; S-02: plant seeds germination in the presence of stimulator S-02; S-01: plant seeds germination in the presence of stimulator S-01. All chemicals were tested in medium MS at concentrations of 10 μM

Compounds, selected from primary pollen screen were further evaluated in secondary screen of Arabidopsis seed germination in order to validate their effects on general plant development. Arabidopsis seeds were germinated in the presence of selected compounds essentially, as described earlier [12]. Nineteen compounds, that were found to inhibit pollen germination and tube growth, inhibited also seed germination and root growth, whereas three compounds, found as stimulators of pollen germination and tube growth, had a similar effect in seed germination and tube growth (Fig. 4, Table 1). These growth inhibitors belong to at least ten diverse chemical classes, including pyrazole, pyrazine, thiourea, thioamide, oxazole, indoline, diazinane, thiazolidine, guaniline and benzazepin (Additional file 4) of 22 compounds selected after primary and secondary screens of 1040 chemicals from Chemical Diversity Research Institute (CDRI) library of chemical compounds). The compounds also cause a reduction in Arabidopsis root length (as % of control) in the range from 4.76 % (I-01) to 49.20 % (I-19), when 7 days old seedlings were grown in the presence of these chemicals. Two chemicals sharing structural homology with thiazolidines (S-01, S-03) stimulated root growth length (as % of control) up to 129.23 and 119.09 %, respectively. The other root growth stimulating compound (S-02) belongs to benzazepin-type chemicals and increased Arabidopsis root length up to 126.24 % as compared to untreated roots (Additional file 4, Fig. 5). We assume that pollen and seed germination are quite similar processes based on common genes and regulatory pathways (Table 1). Results for selected 22 chemical compound hits showed either inhibitory (I) or stimulatory (S) effects in pollen tube and root growth assays. Although germination of seeds occurred after 3 days of culture on agar plates, the final results on seed germination and root growth were obtained only after at least 10–14 days of incubation, thus demonstrating clearly the advantage of pollen system as fast screening system, which can be completed in 2–3 h.

**Table 1:** Chemical Structures and comparison of 22 hit compounds demonstrated either inhibitory (I-) or stimulatory (S-) effects in pollen tube assay with respect to their effects in root growth assays

#	Molecular Structure	Compound Name	Effect onrootgrowth, %
I-01		4‐({6‐[(3‐bromophenyl) amino]‐[1,2,5] oxadiazolo [3,4‐b] pyrazin‐5‐yl} amino) phenol	4,76 %, (*p* < 0.05)
I-02		(4E)‐1‐(3,4‐dichlorophenyl)‐4‐[(4‐hydroxy‐3‐iodo‐5‐methoxyphenyl) methylidene]‐3‐methyl‐4,5‐dihydro‐1H‐pyrazol‐5‐one	6,40 %, (*p* < 0.05)
I-03		(5E)-5-[(4-fluorophenyl) methylidene]-3-[(4-iodoanilino) methyl]-1,3-thiazolidine-2,4-dione	7,10 %, (*p* < 0.05)
I-04		2‐(4‐{[(2Z,5Z)‐4‐oxo‐3‐phenyl‐2‐(phenylimino)‐1,3‐thiazolidin‐5‐ylidene] methyl} phenoxy) acetic acid	7,36 %, (*p* < 0.05)
I-05		ethyl 4-[3-[(3-bromophenyl) methyl]-4-oxo-2-sulfanylidene-1H-quinazoline-7-carbonyl] piperazine-1-carboxylate	8,96 %, (*p* < 0.05)
I-06		3,4-dihydro-2H-quinolin-1-yl-[3-(4-phenylpiperazin-1-yl) sulfonylphenyl] methanone	9,60 %, (*p* < 0.05)
I-07		2-chloro-5-[(4Z)-4-[(3-methoxy-4-phenylmethoxyphenyl) methylidene]-3-methyl-5-oxopyrazol-1-yl] benzoic acid	9,92 %, (*p* < 0.05)
I-08		(4Z)-4-[(2-methoxy-1-naphthyl) methylene]-2-(4-methoxy-3-nitrophenyl)-1,3-oxazol-5 (4H)-one	11,52 %, (*p* < 0.05)
I-09		4-[(4E)-4-[(4,5-dimethoxy-2-nitrophenyl) methylidene]-3-methyl-5-oxopyrazol-1-yl] benzoic acid	12,16 %, (*p* < 0.05)
I-10		N-[(5Z)-5-[(3,4-dimethoxyphenyl) methylidene]-4-oxo-2-sulfanylidene-1,3-thiazolidin-3-yl]-3-nitrobenzamide	14,19 %, (*p* < 0.05)
I-11		(4E)-2-(3,4-dichlorophenyl)-4-[(3,4-dimethoxyphenyl) methylidene]-5-methylpyrazol-3-one	14,41 %, (*p* < 0.05)
I-12		2-phenylethyl 7-(4-chlorophenyl)-4-(4-hydroxy-3-methoxyphenyl)-2-methyl-5-oxo-4,6,7,8-tetrahydro-1H-quinoline-3-carboxylate	16,33 %, (*p* < 0.05)
I-13		4-[[2-bromo-4-[(2,4,6-trioxo-1,3-diazinan-5-ylidene) methyl] phenoxy] methyl] benzoic acid	16,97 %, (*p* < 0.05)
I-14		methyl 4-[[(5E)-5-[(4-methylsulfanylphenyl) methylidene]-2,4-dioxo-1,3-thiazolidin-3-yl] methylamino] benzoate	17,70 %, (*p* < 0.05)
I-15		4-[4-(dimethylamino) phenyl]-8-{(E)-1-[4-(dimethylamino) phenyl] methylidene}-3,4,5,6,7,8-hexahydro-2 (1H)-quinazolinethione	19,74 %, (*p* < 0.05)
I-16		[3-(1,3-benzodioxol-5-yl)-2-methyl-4-oxo-6-propylchromen-7-yl] acetate	27,09 %, (*p* < 0.05)
I-17		2-(7,7-dimethyl-3-oxobicyclo [2.2.1] hept-2-yliden)-N-(4-methylphenyl)-1-hydrazinecarbothioamide	29,82 %, (*p* < 0.05)
I-18		2-(9H-xanthen-9-yl)-1H-indene-1,3 (2H)-dione	48,49 %, (*p* < 0.05)
I-19		2-{4-[(isopentyloxy) carbonyl] phenyl}-1,3-dioxo-5-isoindolinecarboxylic acid	49,20 %, (*p* < 0.05)
S-01		4-[bis (2-methoxyethyl) sulfamoyl]-N-[4-(4-nitrophenyl)-1,3-thiazol-2-yl] benzamide	119,09 %, (*p* < 0.05)
S-02		(3-Chloro-1-benzothiophen-2-yl) (10,11-dihydro-5H-dibenzo [b,f] azepin-5-yl) methanone	126,24 %, (*p* < 0.05)
S-03		N-[4-(4-methylphenyl)-1,3-thiazol-2-yl]-2-phenoxybenzamide	129,23 %, (*p* < 0.05)

**Fig. 5:**
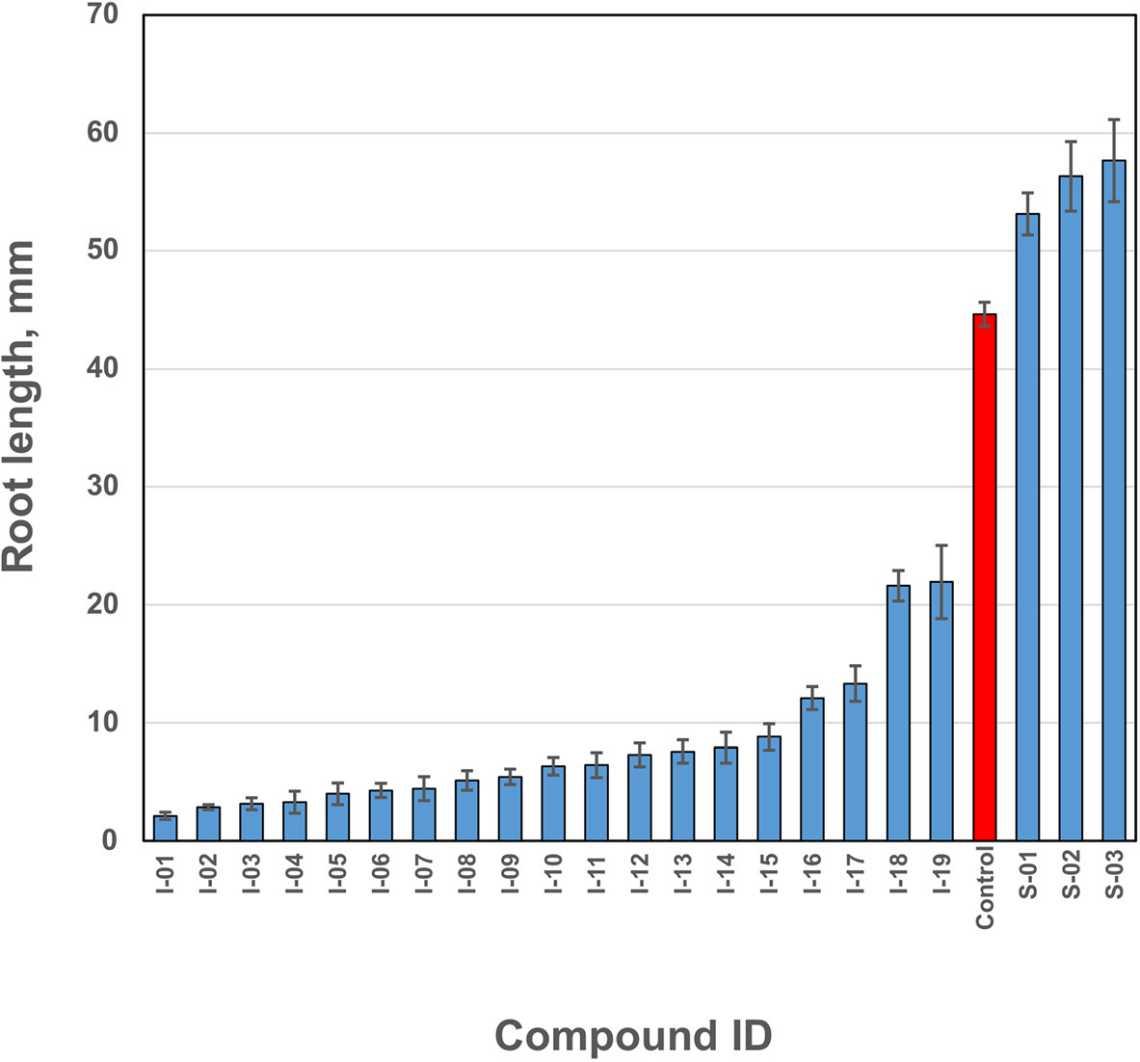
Effect of chemical compounds on Arabidopsis root length. Arabidopsis seeds were germinated on agar plates, supplemented with tested chemicals at concentrations of 10 μM. Then, root length was measured in mm. Data represent the means ± SE (n ≥10seeds). Control – Arabidopsis seeds, germinated without tested compounds

## Discussion

We have shown that a small chemical screen using pollen harvested from few flowering plants is not only one of the fastest systems for HTS, but also versatile as pollen from virtually any plant can be used. This broadens the screening space by linking biological diversity with the available chemical space. The success of the screen depends on the quality of the pollen grains to readily germinate under experimental conditions.

An optimization phase may be necessary for different plant species, but should take only a short time and effort to obtain satisfying conditions based on statistical analysis and enable robust, sensitive, reliable and reproducible screens. Rescreening of hits in different conditions and biological context such as germinating roots from seeds of same plants can be completed in less than 2 weeks. Our study demonstrates that broad versatility of the screen in which compounds either stimulating or inhibiting pollen tube growth are also effective in seed germination and root growth assays thereby suggesting shared targets in growth regulating pathways.

## Conclusion

In this study, we demonstrated the usefulness of plant pollen tubes for screening small chemical compound libraries for new biologically active compounds. The pollen tubes represent an ultra-rapid screening tool with which even large compound libraries can be analyzed in very short time intervals. The broadly applicable high-throughput protocol is suitable for automated phenotypic screening of germinating pollen resulting in combination with seed germination assays in identification of plant growth inhibitors and stimulators.

## Methods

### Plant materials

*Nicotiana tabacum L*. cv. SR1 seeds were kindly provided by Prof. A. Bachmair (University of Vienna, Austria). Plants were grown in greenhouse under long-day photoperiod (16 h-light/8 h-dark, 24 °C) with an illumination of 100–150 μE m^−2^ s^−1^ and 60–70 % relative humidity for 6–8 weeks with regular supply of fertilizers and routine watering until intensive flowering and pollen dispersal. Continuous flowering can be achieved by regular harvest of open flowers. Determination of pollen developmental stage was performed by DAPI (Invitrogen, cat. no. D1306) staining (DAPI stock solution: 5 mg DAPI in 1 ml 50 % ethanol). Flower buds at different developmental stages were collected, anthers were dissected from flower buds and placed on a glass slide in a drop of DAPI working solution (DAPI working solution: dilute the DAPI stock solution 1:2000 in 1x Pollen Isolation Buffer (PIB buffer). Anthers were squashed gently by the forceps on the slide to release pollen grains and the slide was covered with a glass coverslip. Preparations were incubated incubate for 5 min at room temperature and observed under a fluorescence microscope using a DAPI filter set.

### Pollen collection and preparation

Pollen grains were removed from anthers of open flowers using gentle shaking and collected into centrifuge tubes (volume 50 ml) containing GVH14 [25] medium (708 mg of Ca (NO_3_)_2_ x 4H_2_O, 100 mg of KNO_3_, 200 mg MgSO_4_ x 7H_2_O, 14 mg H_2_BO_3_, 1 g of Casein hydrolysate, 10 g of sucrose, and 0.5 g of 2-(N-morpholino) ethansulfonic acid (MES) dissolved in 1 l distilled water, pH = 5.9, filter sterilized). Pollen suspension was diluted to a density of 4.000 pollen grains per ml. At least 30–35 flowers were used for one experiment. Pollen suspension was filtered using the nylon mesh (40 μm) and clean pollen suspension was collected in another centrifuge tube.

### Chemical screening and treatment

Chemicals were generously provided by the Chemical Diversity Research Institute (CDRI, Moscow, Russia). All chemicals were dissolved at 100 μM GVH14 supplemented with 14 mg boric acid, and then dispensed by 100 μl per well using BiomekFXP robot system into 96-well plate as follows: 99 μl of medium GVH and 1 μl of chemical compound from 10 mM stock solution (compound library) were added into each well into the columns from 2 to 11 by using 96-tips of Biomek FXP to reach the 100 μM final concentration of the compound. In some wells 1 μl from 1 mM stock solution of salicylic acid (SA) was added into the wells from A1 to D1 and from E12 to H12 (marked as min in Additional file 5). Pollen suspensions were loaded into 384-well plates to final density of 4.000 pollen grains per ml pollen. This generates five 384-well plates for screening of more than 1.000 compounds. In each well 45 μl pollen suspension were automatically added by robotic system. For screening 20 μl compounds were automatically added from 100 μM stock solution to each well, and plates incubated at room temperature for 2 h, or stored at 4 °C until start of the screening procedure, but not longer than 4 h. For phenotypic screening plates were opened and four images per well were taken in transmitted light using 10x objective in ImageXpress Micro XL (Wide field High Content Screening System (Molecular Devices, USA). In total, 1536 images were taken for one plate. Data analysis was conducted using the algorithm, developed based on Custom Module Editor software with registration of individual pollen square (Molecular Devices, USA). Images were processed to define the objects of interest (pollen grains) based on the intensity of images and size of objects.

### Assessment of assay variability

Assay was performed in duplicates and during 3 consecutive days on 384-well plates with pollen adjusted to the maximum signal in control plates (medium GVH14 with 0.1 % DMSO). The mean and standard deviation were calculated for each maximum and minimum signal control plates. The data from two 384-multi-well maximum signal control plates were combined to obtain a mean and SD for the replicates. Finally, the combined data of the maximum and minimum plates were used to calculate the Z-factor.

### Seed germination and root growth test

Approximately 20 sterilized seeds were used for germination in Murashige and Skoog medium (MS-medium) (1.7 g of (MS) macro salt mixture, 1 g of (MS) micro salt mixture, 1 ml of 1000x (MS) vitamin stock solution and 0.5 g of MES hydrate and 10 g phytagar in 1 L deionized water, pH = 5.7, sterilize by autoclaving 15 min at 121 °C) in a Petri dish, vernalized for 2 to 3 days at 4 °C in the dark, and then transferred to a plant growth room (21–25 °C, 16 h photoperiod). The primary root length was measured after 10 days of growth. In each case at least 20 seedlings were measured. The experiments were repeated at least twice using different lots of seeds.

## Additional files

Additional file 1: Table S1. Statistical analysis of the assay data to interpret variability. The mean and standard deviation (SD) are calculated for each maximum (max) and minimum (min) signal plates for pollen assay to obtain the coefficient of variation (% CV) for each plate (intra-plate variability). The data from the maximum and minimum replicate plates are then combined to obtain a new mean and SD. These data are used to calculate the Z-factor (four plates testing) for each day. Finally, all of the data from all the maximum and minimum plates are combined to obtain the mean, SD and interplate variability. (DOCX 16 kb) Additional file 2: Table S2. Listing 1040 chemical compounds library screened on pollen cells. (DOCX 985 kb) Additional file 3: Table S3. Listing 65 chemical compounds screened in both assays: pollen germination and root growth. (DOCX 15 kb) Additional file 4: Table S4. Chemical diversity vs biological activity (root length and root growth (% of zero control plant growth) of 22 compounds selected after primary and secondary screens of 1040 chemicals from CDRI library of chemical compounds. (TIF 7536 kb) Additional file 5: Figure S1. General scheme of dispensing tested compounds and control compounds in 96-well plate. Columns 1 and 12 were used for control compounds. Wells A1:D1, E12:H12 were used as negative controls for the minimum signal (*n* =8). Wells E1:H1, A12:D12 are used as positive controls for the maximum signal (*n* = 8). All the remaining wells were used for tested chemicals from compound libraries (columns 2–11; *n* = 80). Positive controls and tested compounds were re-suspended in medium GVH14 with 0.1 % DMSO. Negative controls were re-suspended in medium GVH14 with 0.1 % DMSO in the presence of 1 μM salicylic acid. (DOCX 72 kb)

## Abbreviations

CDRI: Chemcial Diversity Research Institute
DAPI: 4′,6-diamidino-2-phenylindole
DMSO: Dimethyl sulfoxide
HTS: High-throughput screening
MES: 2-(N-morpholino) ethanesulfonic acid
MS-medium: Murashige and skoog medium
PIB: Pollen isolation buffer
SD: Standard deviation
