# Supplementary material for: Novel small molecule modulators of plant growth and development identified by high-content screening with plant pollen
Source: BMC Plant Biol. 2016 Sep 6;16(1):192. doi: 10.1186/s12870-016-0875-4 (PMC5011872; doi:10.1186/s12870-016-0875-4)
Supplement: Additional file 1: Table S1. — Statistical analysis of the assay data to interpret variability. The mean and standard deviation (SD) are calculated for each maximum (max) and minimum (min) signal plates for pollen assay to obtain the coefficient of variation (% CV) for each plate (intra-plate variability). The data from the maximum and minimum replicate plates are then combined to obtain a new mean and SD. These data are used to calculate the Z-factor (four plates testing) for each day. Finally, all of the data from all the maximum and minimum plates are combined to obtain the mean, SD and interplate variability. (DOCX 16 kb) [file 12870_2016_875_MOESM1_ESM.docx]

|  |  |  |  | **Area of objects, µM** | |  |  |
| --- | --- | --- | --- | --- | --- | --- | --- |
| **Variability** |  |  |  | **mean** | **sd** | **cv** | **Z-factor** |
| **Intra plate** | **day 1** | plate 1 | min | 105.428 | 8.014 | 7.60 |  |
|  |  | plate 2 | min | 113.197 | 7.525 | 6.65 |  |
|  |  | plate 3 | max | 271.151 | 19.451 | 7.17 |  |
|  |  | plate 4 | max | 295.720 | 27.688 | 9.36 | 0.46 |
|  | **day 2** | plate 1 | min | 122.329 | 7.015 | 5.73 |  |
|  |  | plate 2 | min | 119.456 | 9.287 | 7.77 |  |
|  |  | plate 3 | max | 298.635 | 23.208 | 7.77 |  |
|  |  | plate 4 | max | 307.855 | 20.533 | 6.67 | 0.49 |
|  | **day 3** | plate 1 | min | 102.113 | 9.102 | 8.91 |  |
|  |  | plate 2 | min | 112.972 | 8.215 | 7.27 |  |
|  |  | plate 3 | max | 269.644 | 24.199 | 8.97 |  |
|  |  | plate 4 | max | 288.534 | 22.740 | 7.88 | 0.44 |
|  | **day 4** | plate 1 | min | 110.325 | 9.096 | 8.24 |  |
|  |  | plate 2 | min | 118.516 | 7.214 | 6.09 |  |
|  |  | plate 3 | max | 281.714 | 20.408 | 7.24 |  |
|  |  | plate 4 | max | 295.428 | 30.841 | 10.44 | 0.42 |
| **Inter plate** |  | All plates | min | 113.042 | 7.699 | 6.81 |  |
|  |  | All plates | max | 288.590 | 13.522 | 4.69 | 0.45 |

**Additional file 1: Table S1.** Statistical analysis of the assay data to interpret variability

The mean and standard deviation (SD) are calculated for each maximum (max) and minimum (min) signal plates for pollen assay to obtain the coefficient of variation (% CV) for each plate (intra-plate variability). The data from the maximum and minimum replicate plates are then combined to obtain a new mean and SD. These data are used to calculate the Z-factor (four plates testing) for each day. Finally, all of the data from all the maximum and minimum plates are combined to obtain the mean, SD and interplate variability.
