# Supplementary material for: Novel small molecule modulators of plant growth and development identified by high-content screening with plant pollen
Source: BMC Plant Biol. 2016 Sep 6;16(1):192. doi: 10.1186/s12870-016-0875-4 (PMC5011872; doi:10.1186/s12870-016-0875-4)
Supplement: Additional file 2: Table S2. — Listing 1040 chemical compounds library screened on pollen cells. (DOCX 985 kb) [file 12870_2016_875_MOESM2_ESM.docx]

**Additional file 3:Table S3.** Listing 65 chemical compounds screened in both assays: pollen germination and root growth.

| # | Molecular Structure | Compound Name |
| --- | --- | --- |
| I-01 | 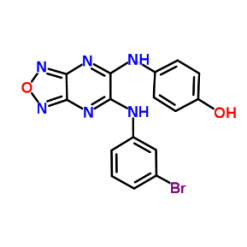 | 4‐({6‐[(3‐bromophenyl)amino]‐[1,2,5]oxadiazolo[3,4‐b]pyrazin‐5‐yl}amino)phenol |
| I-02 | 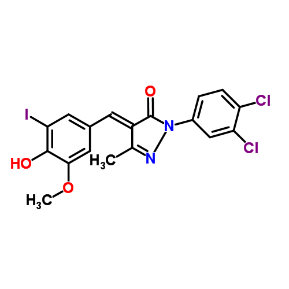 | (4E)‐1‐(3,4‐dichlorophenyl)‐4‐[(4‐hydroxy‐3‐iodo‐5‐methoxyphenyl)methylidene]‐3‐methyl‐4,5‐dihydro‐1H‐pyrazol‐5‐one |
| I-03 | 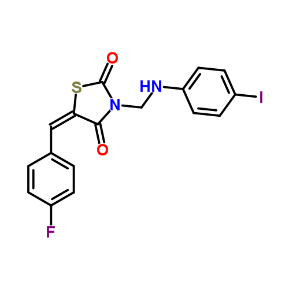 | (5E)-5-[(4-fluorophenyl)methylidene]-3-[(4-iodoanilino)methyl]-1,3-thiazolidine-2,4-dione |
| I-04 | 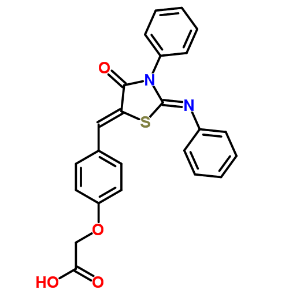 | 2‐(4‐{[(2Z,5Z)‐4‐oxo‐3‐phenyl‐2‐(phenylimino)‐1,3‐thiazolidin‐5‐ylidene]methyl}phenoxy)acetic acid |
| I-05 | 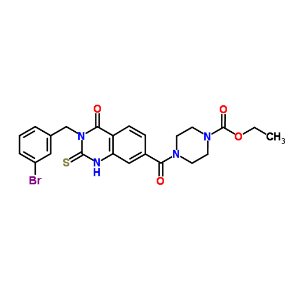 | ethyl 4-[3-[(3-bromophenyl)methyl]-4-oxo-2-sulfanylidene-1H-quinazoline-7-carbonyl]piperazine-1-carboxylate |
| I-06 | 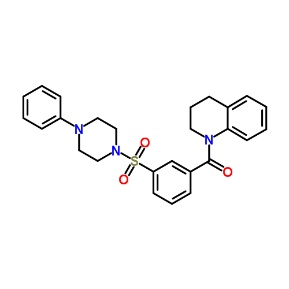 | 3,4-dihydro-2H-quinolin-1-yl-[3-(4-phenylpiperazin-1-yl)sulfonylphenyl]methanone |
| I-07 | 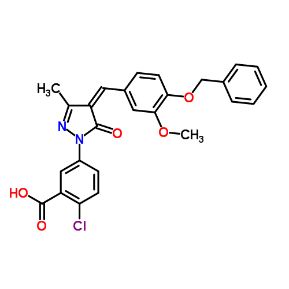 | 2-chloro-5-[(4Z)-4-[(3-methoxy-4-phenylmethoxyphenyl)methylidene]-3-methyl-5-oxopyrazol-1-yl]benzoic acid |
| I-08 | 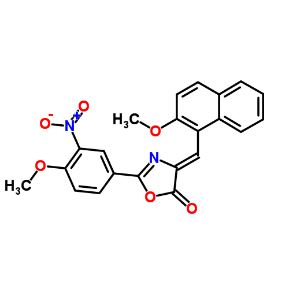 | (4Z)-4-[(2-methoxy-1-naphthyl)methylene]-2-(4-methoxy-3-nitrophenyl)-1,3-oxazol-5(4H)-one |
| I-09 | 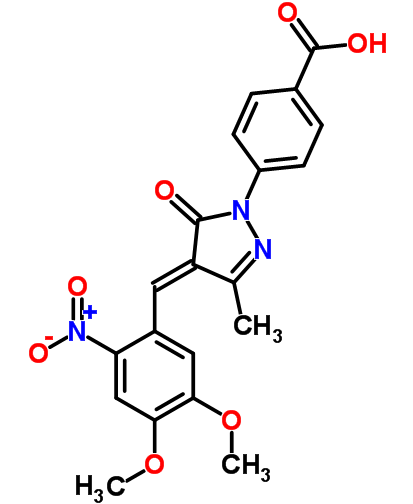 | 4-[(4E)-4-[(4,5-dimethoxy-2-nitrophenyl)methylidene]-3-methyl-5-oxopyrazol-1-yl]benzoic acid |
| I-10 | 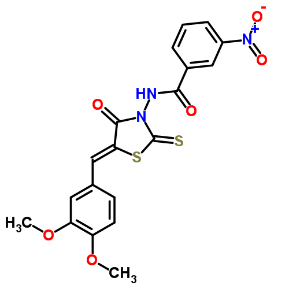 | N-[(5Z)-5-[(3,4-dimethoxyphenyl)methylidene]-4-oxo-2-sulfanylidene-1,3-thiazolidin-3-yl]-3-nitrobenzamide |
| I-11 | 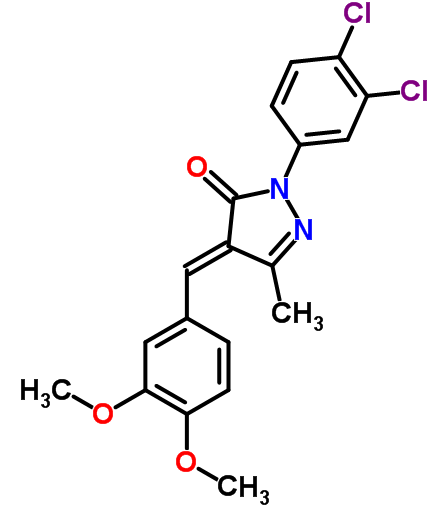 | (4E)-2-(3,4-dichlorophenyl)-4-[(3,4-dimethoxyphenyl)methylidene]-5-methylpyrazol-3-one |
| I-12 | 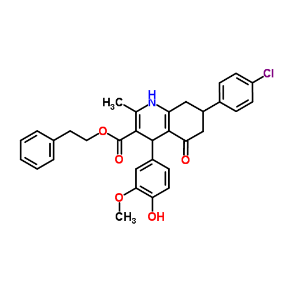 | 2-phenylethyl 7-(4-chlorophenyl)-4-(4-hydroxy-3-methoxyphenyl)-2-methyl-5-oxo-4,6,7,8-tetrahydro-1H-quinoline-3-carboxylate |
| I-13 | 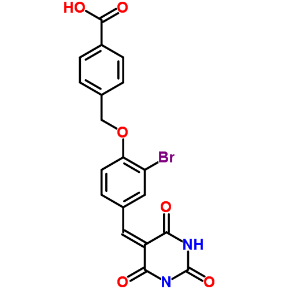 | 4-[[2-bromo-4-[(2,4,6-trioxo-1,3-diazinan-5-ylidene)methyl]phenoxy]methyl]benzoic acid |
| I-14 | 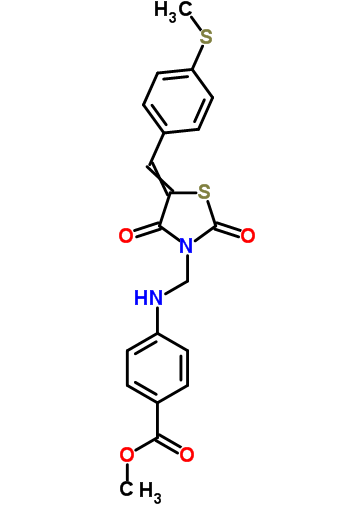 | methyl 4-[[(5E)-5-[(4-methylsulfanylphenyl)methylidene]-2,4-dioxo-1,3-thiazolidin-3-yl]methylamino]benzoate |
| I-15 | 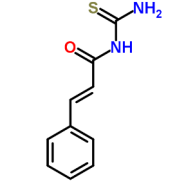 | 4-[4-(dimethylamino)phenyl]-8-{(E)-1-[4-(dimethylamino)phenyl]methylidene}-3,4,5,6,7,8-hexahydro-2(1H)-quinazolinethione |
| I-16 | 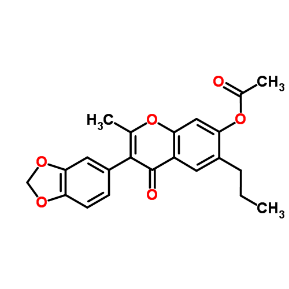 | [3-(1,3-benzodioxol-5-yl)-2-methyl-4-oxo-6-propylchromen-7-yl] acetate |
| I-17 | 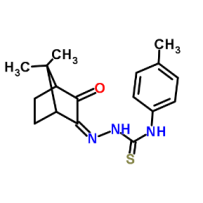 | 2-(7,7-dimethyl-3-oxobicyclo[2.2.1]hept-2-yliden)-N-(4-methylphenyl)-1-hydrazinecarbothioamide |
| I-18 | 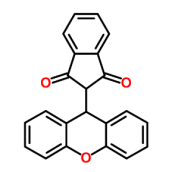 | 2-(9H-xanthen-9-yl)-1H-indene-1,3(2H)-dione |
| I-19 | 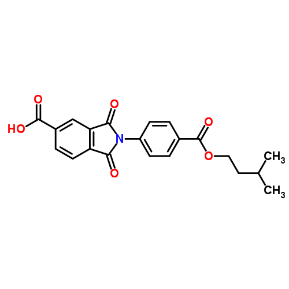 | 2-{4-[(isopentyloxy)carbonyl]phenyl}-1,3-dioxo-5-isoindolinecarboxylic acid |
| I-20 | 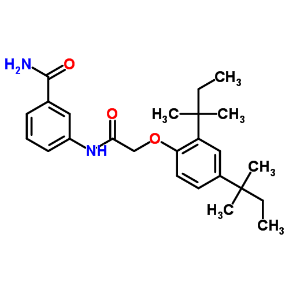 | 3-({2-[2,4-di(tert-pentyl)phenoxy]acetyl}amino)benzamide |
| I-21 | 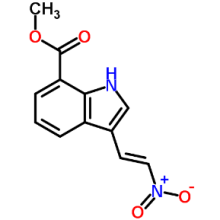 | methyl 3-[(E)-2-nitro-1-ethenyl]-1H-indole-7-carboxylate |
| I-22 | 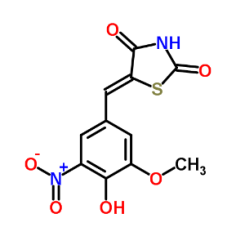 | 5-[(E)-1-(4-hydroxy-3-methoxy-5-nitrophenyl)methylidene]-1,3-thiazolane-2,4-dione |
| I-23 | 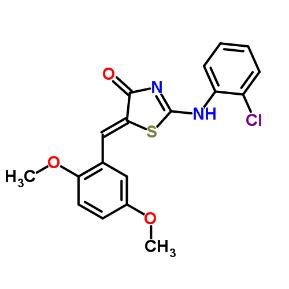 | (5Z)-2-(2-chloroanilino)-5-[(2,5-dimethoxyphenyl)methylidene]-1,3-thiazol-4-one |
| I-24 | 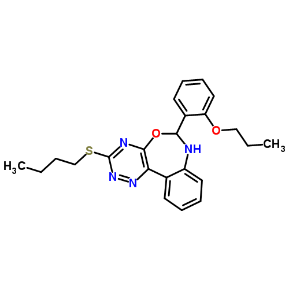 | 3-butylsulfanyl-6-(2-propoxyphenyl)-6,7-dihydro-[1,2,4]triazino[5,6-d][3,1]benzoxazepine |
| I-25 | 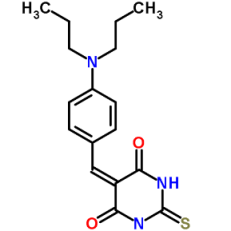 | 5-[[4-(dipropylamino)phenyl]methylidene]-2-sulfanylidene-1,3-diazinane-4,6-dione |
| I-26 | 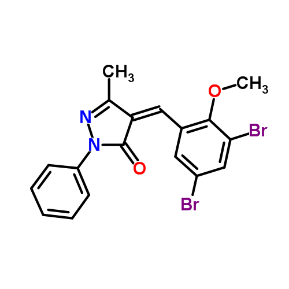 | (4Z)-4-[(3,5-dibromo-2-methoxyphenyl)methylidene]-5-methyl-2-phenylpyrazol-3-one |
| I-27 | 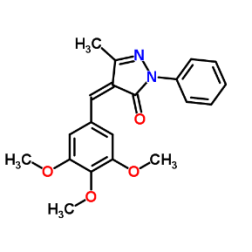 | (4Z)-5-methyl-2-phenyl-4-[(3,4,5-trimethoxyphenyl)methylidene]pyrazol-3-one |
| I-28 | 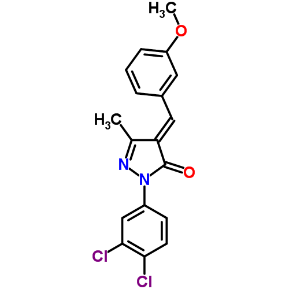 | (4E)-2-(3,4-dichlorophenyl)-4-[(3-methoxyphenyl)methylidene]-5-methylpyrazol-3-one |
| I-29 | 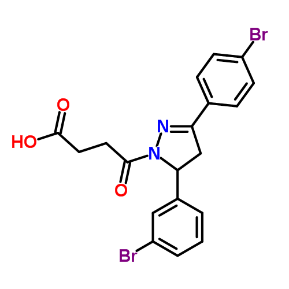 | 4-[3-(3-bromophenyl)-5-(4-bromophenyl)-3,4-dihydropyrazol-2-yl]-4-oxobutanoic acid |
| I-30 | 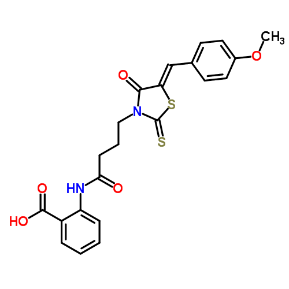 | 2-[4-[(5Z)-5-[(4-methoxyphenyl)methylidene]-4-oxo-2-sulfanylidene-1,3-thiazolidin-3-yl]butanoylamino]benzoic acid |
| I-31 | 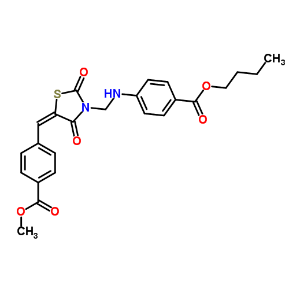 | methyl 4-[(E)-[3-[(4-butoxycarbonylanilino)methyl]-2,4-dioxo-1,3-thiazolidin-5-ylidene]methyl]benzoate |
| I-32 | 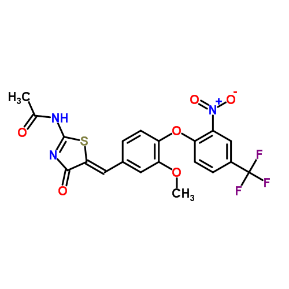 | N-[(5Z)-5-[[3-methoxy-4-[2-nitro-4-(trifluoromethyl)phenoxy]phenyl]methylidene]-4-oxo-1,3-thiazol-2-yl]acetamide |
| I-33 | 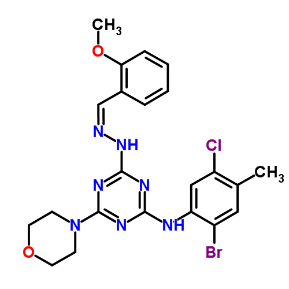 | 4-N-(2-bromo-5-chloro-4-methylphenyl)-2-N-[(Z)-(2-methoxyphenyl)methylideneamino]-6-morpholin-4-yl-1,3,5-triazine-2,4-diamine |
| I-34 | 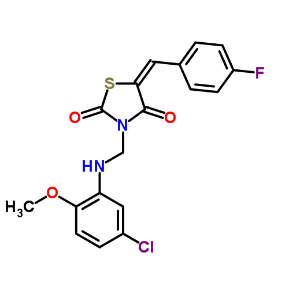 | (5E)-3-[(5-chloro-2-methoxyanilino)methyl]-5-[(4-fluorophenyl)methylidene]-1,3-thiazolidine-2,4-dione |
| I-35 | 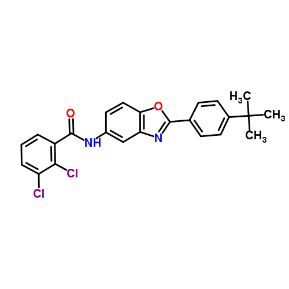 | N-[2-(4-tert-butylphenyl)-1,3-benzoxazol-5-yl]-2,3-dichlorobenzamide |
| I-36 | 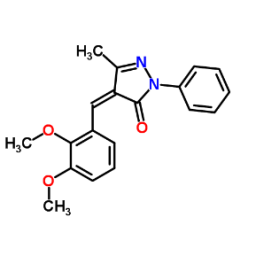 | (4Z)-4-[(2,3-dimethoxyphenyl)methylidene]-5-methyl-2-phenylpyrazol-3-one |
| I-37 | 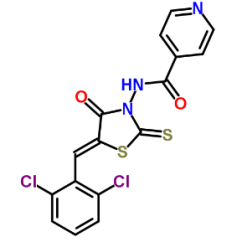 | N-[(5Z)-5-[(2,6-dichlorophenyl)methylidene]-4-oxo-2-sulfanylidene-1,3-thiazolidin-3-yl]pyridine-4-carboxamide |
| I-38 | 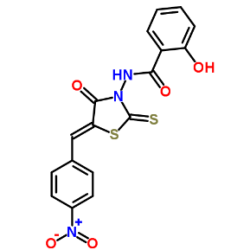 | 2-hydroxy-N-[(5Z)-5-[(4-nitrophenyl)methylidene]-4-oxo-2-sulfanylidene-1,3-thiazolidin-3-yl]benzamide |
| I-39 | 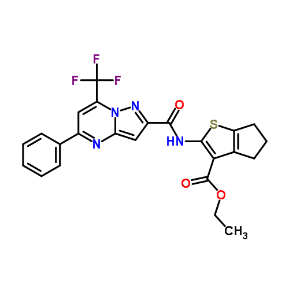 | ethyl 2-[[5-phenyl-7-(trifluoromethyl)pyrazolo[1,5-a]pyrimidine-2-carbonyl]amino]-5,6-dihydro-4H-cyclopenta[b]thiophene-3-carboxylate |
| I-40 | 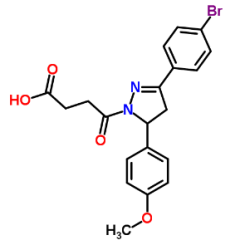 | 4-[5-(4-bromophenyl)-3-(4-methoxyphenyl)-3,4-dihydropyrazol-2-yl]-4-oxobutanoic acid |
| I-41 | 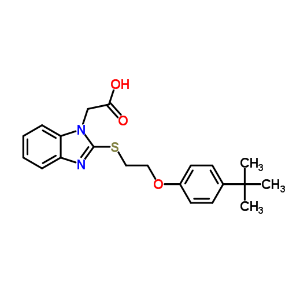 | 2-[2-[2-(4-tert-butylphenoxy)ethylsulfanyl]benzimidazol-1-yl]acetic acid |
| I-42 | 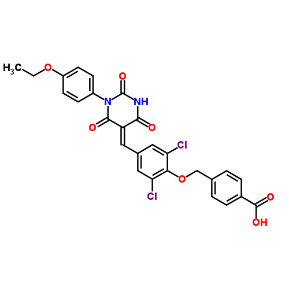 | 4-[[2,6-dichloro-4-[(E)-[1-(4-ethoxyphenyl)-2,4,6-trioxo-1,3-diazinan-5-ylidene]methyl]phenoxy]methyl]benzoic acid |
| I-43 | 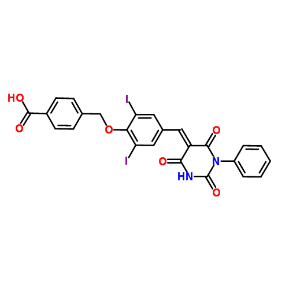 | 4-[[2,6-diiodo-4-[(E)-(2,4,6-trioxo-1-phenyl-1,3-diazinan-5-ylidene)methyl]phenoxy]methyl]benzoic acid |
| I-44 | 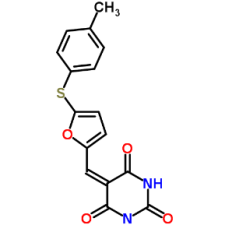 | 5-[[5-(4-methylphenyl)sulfanylfuran-2-yl]methylidene]-1,3-diazinane-2,4,6-trione |
| I-45 | 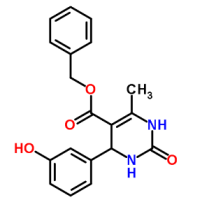 | benzyl 4-(3-hydroxyphenyl)-6-methyl-2-oxo-3,4-dihydro-1H-pyrimidine-5-carboxylate |
| I-46 | 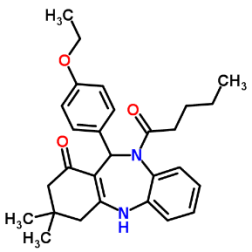 | 11-(4-ethoxyphenyl)-3,3-dimethyl-10-pentanoyl-2,3,4,5,10,11-hexahydro-1H-dibenzo[b,e][1,4]diazepin-1-one |
| I-47 | 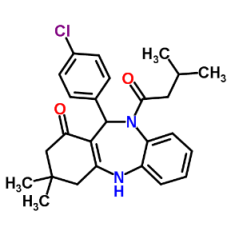 | 11-(4-chlorophenyl)-3,3-dimethyl-10-(3-methylbutanoyl)-2,3,4,5,10,11-hexahydro-1H-dibenzo[b,e][1,4]diazepin-1-one |
| I-48 | 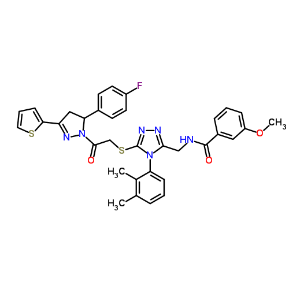 | N-[[4-(2,3-dimethylphenyl)-5-[2-[3-(4-fluorophenyl)-5-thiophen-2-yl-3,4-dihydropyrazol-2-yl]-2-oxoethyl]sulfanyl-1,2,4-triazol-3-yl]methyl]-3-methoxybenzamide |
| I-49 | 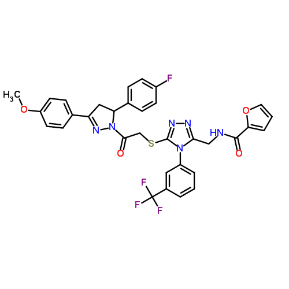 | N-[[5-[2-[3-(4-fluorophenyl)-5-(4-methoxyphenyl)-3,4-dihydropyrazol-2-yl]-2-oxoethyl]sulfanyl-4-[3-(trifluoromethyl)phenyl]-1,2,4-triazol-3-yl]methyl]furan-2-carboxamide |
| I-50 | 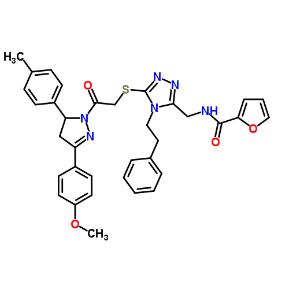 | N-[[5-[2-[5-(4-methoxyphenyl)-3-(4-methylphenyl)-3,4-dihydropyrazol-2-yl]-2-oxoethyl]sulfanyl-4-(2-phenylethyl)-1,2,4-triazol-3-yl]methyl]furan-2-carboxamide |
| I-51 | 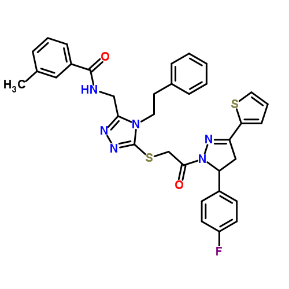 | N-[[5-[2-[3-(4-fluorophenyl)-5-thiophen-2-yl-3,4-dihydropyrazol-2-yl]-2-oxoethyl]sulfanyl-4-(2-phenylethyl)-1,2,4-triazol-3-yl]methyl]-3-methylbenzamide |
| I-52 | 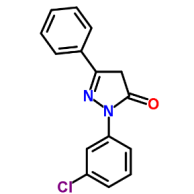 | 2-(3-chlorophenyl)-5-phenyl-4H-pyrazol-3-one |
| I-53 | 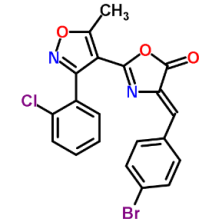 | (4Z)-4-(4-bromobenzylidene)-2-[3-(2-chlorophenyl)-5-methyl-1,2-oxazol-4-yl]-1,3-oxazol-5(4H)-one |
| S-01 | 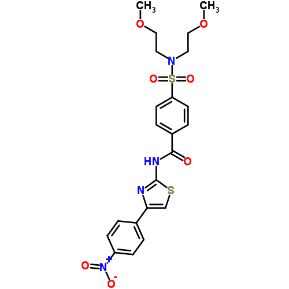 | 4-[bis(2-methoxyethyl)sulfamoyl]-N-[4-(4-nitrophenyl)-1,3-thiazol-2-yl]benzamide |
| S-02 | 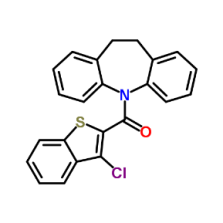 | (3-Chloro-1-benzothiophen-2-yl)(10,11-dihydro-5H-dibenzo[b,f]azepin-5-yl)methanone |
| S-03 | 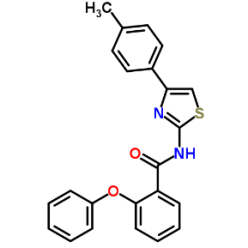 | N-[4-(4-methylphenyl)-1,3-thiazol-2-yl]-2-phenoxybenzamide |
| S-04 | 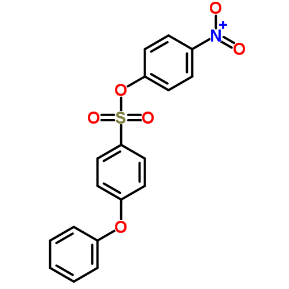 | 4-nitrophenyl 4-phenoxy-1-benzenesulfonate |
| S-05 | 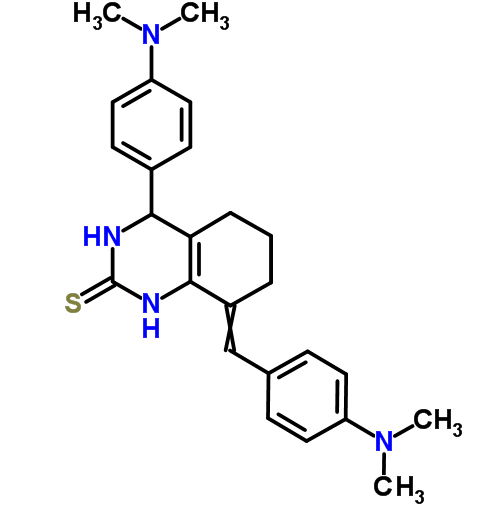 | 4-[4-(dimethylamino)phenyl]-8-{(E)-1-[4-(dimethylamino)phenyl]methylidene}-3,4,5,6,7,8-hexahydro-2(1H)-quinazolinethione |
| S-06 | 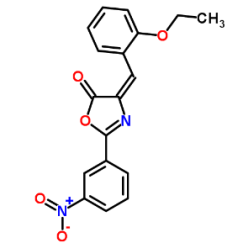 | (4E)-4-(2-Ethoxybenzylidene)-2-(3-nitrophenyl)-1,3-oxazol-5(4H)-one |
| S-07 | 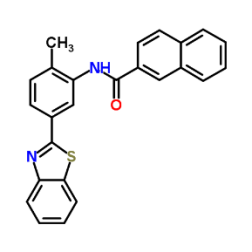 | N-[5-(1,3-benzothiazol-2-yl)-2-methylphenyl]naphthalene-2-carboxamide |
| S-08 | 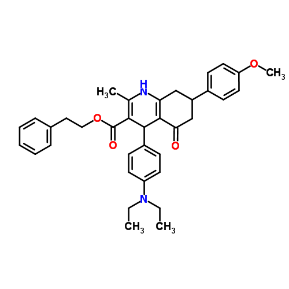 | 2-phenylethyl 4-[4-(diethylamino)phenyl]-7-(4-methoxyphenyl)-2-methyl-5-oxo-4,6,7,8-tetrahydro-1H-quinoline-3-carboxylate |
| S-09 | 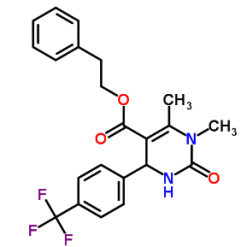 | 2-phenylethyl 3,4-dimethyl-2-oxo-6-[4-(trifluoromethyl)phenyl]-1,6-dihydropyrimidine-5-carboxylate |
| S-10 | 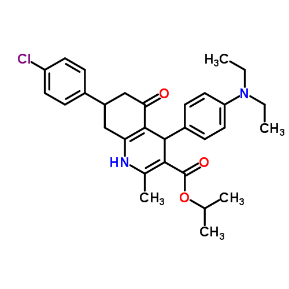 | propan-2-yl 7-(4-chlorophenyl)-4-[4-(diethylamino)phenyl]-2-methyl-5-oxo-4,6,7,8-tetrahydro-1H-quinoline-3-carboxylate |
| S-11 | 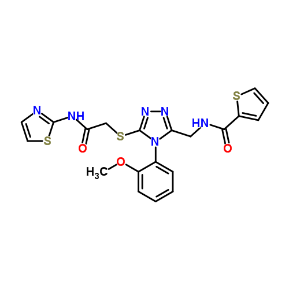 | N-[[4-(2-methoxyphenyl)-5-[2-oxo-2-(1,3-thiazol-2-ylamino)ethyl]sulfanyl-1,2,4-triazol-3-yl]methyl]thiophene-2-carboxamide |
| S-12 | 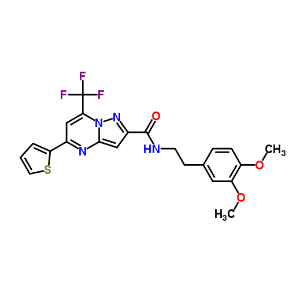 | N-[2-(3,4-dimethoxyphenyl)ethyl]-5-thiophen-2-yl-7-(trifluoromethyl)pyrazolo[1,5-a]pyrimidine-2-carboxamide |
