# Supplementary material for: Novel small molecule modulators of plant growth and development identified by high-content screening with plant pollen
Source: BMC Plant Biol. 2016 Sep 6;16(1):192. doi: 10.1186/s12870-016-0875-4 (PMC5011872; doi:10.1186/s12870-016-0875-4)
Supplement: Additional file 3: Table S3. — Listing 65 chemical compounds screened in both assays: pollen germination and root growth. (DOCX 15 kb) [file 12870_2016_875_MOESM3_ESM.docx]

| **Substance ID** | **Chemical nomenclature** | **Root length (mm)** | **Root growth (% of control)** |
| --- | --- | --- | --- |
| I-01 | Pyrazine | 2.13 ± 0.31 | 4.76 (p < 0.05) |
| I-02 | Pyrazole | 2.86 ± 0.22 | 6.40 (p < 0.05) |
| I-03 | Thiazolidine | 3.17 ± 0.51 | 7.10 (p < 0.05) |
| I-04 | Thiazolidine | 3.29 ± 0.94 | 7.36 (p < 0.05) |
| I-05 | Quinoline | 4.0 ± 0.91 | 8.96 (p < 0.05) |
| I-06 | Quinoline | 4.29 ± 0.60 | 9.60 (p < 0.05) |
| I-07 | Pyrazole | 4.43 ± 1.01 | 9.92 (p < 0.05) |
| I-08 | Oxazole | 5.14 ± 0.83 | 11.52 (p < 0.05) |
| I-09 | Pyrazole | 5.43 ± 0.66 | 12.16 (p < 0.05) |
| I-10 | Thiazolidine | 6.33 ± 0.74 | 14.19 (p < 0.05) |
| I-11 | Pyrazole | 6.43 ± 1.05 | 14.41 (p < 0.05) |
| I-12 | Quinoline | 7.29 ± 1.00 | 16.33 (p < 0.05) |
| I-13 | Diazinane | 7.57 ± 0.98 | 16.97 (p < 0.05) |
| I-14 | Thiazolidine | 7.90 ± 1.30 | 17.70 (p < 0.05) |
| I-15 | Thiourea | 8.81 ± 1.11 | 19.74 (p < 0.05) |
| I-16 | Benzodioxol | 12.09 ± 0.96 | 27.09 (p < 0.05) |
| I-17 | Thioamide | 13.31 ± 1.51 | 29.82 (p < 0.05) |
| I-18 | Xanthenyl | 21.64 ± 1.30 | 48.49 (p < 0.05) |
| I-19 | Indoline | 21.95 ± 3.12 | 49.20 (p < 0.05) |
| S-01 | Thiazolidine | 53.14 ± 1.79 | 119.09 (p < 0.05) |
| S-02 | Benzazepine | 56.33 ± 2.95 | 126.24 (p < 0.05) |
| S-03 | Thiazolidine | 57.67 ± 3.48 | 129.23 (p < 0.05) |

**Additional file 4: Table S4.** Chemical diversity vs biological activity (root length and root growth (% of zero control plant growth) of 22 compounds selected after primary and secondary screens of 1040 chemicals from CDRI library of chemical compounds.
