# Supplementary material for: Novel small molecule modulators of plant growth and development identified by high-content screening with plant pollen
Source: BMC Plant Biol. 2016 Sep 6;16(1):192. doi: 10.1186/s12870-016-0875-4 (PMC5011872; doi:10.1186/s12870-016-0875-4)
Supplement: Additional file 5: Figure S1. — General scheme of dispensing tested compounds and control compounds in 96-well plate. Columns 1 and 12 were used for control compounds. Wells A1:D1, E12:H12 were used as negative controls for the minimum signal (n =8). Wells E1:H1, A12:D12 are used as positive controls for the maximum signal (n = 8). All the remaining wells were used for tested chemicals from compound libraries (columns 2–11; n = 80). Positive controls and tested compounds were re-suspended in medium GVH14 with 0.1 % DMSO. Negative controls were re-suspended in medium GVH14 with 0.1 % DMSO in the presence of 1 μM salicylic acid. (DOCX 72 kb) [file 12870_2016_875_MOESM5_ESM.docx]

|  | **Additional file2:Table S2. Listing 1040 chemical compounds library screened on pollen cells.Additional file2:Table S2. Listing 1040 chemical compounds library screened on pollen cells.Additional file2:Table S2. Listing 1040 chemical compounds library screened on pollen cells.Additional file2:Table S2. Listing 1040 chemical compounds library screened on pollen cells.Additional file2:Table S2. Listing 1040 chemical compounds library screened on pollen cells.Additional file2:Table S2. Listing 1040 chemical compounds library screened on pollen cells.Additional file2:Table S2. Listing 1040 chemical compounds library screened on pollen cells.Additional file2:Table S2. Listing 1040 chemical compounds library screened on pollen cells.Additional file2:Table S2. Listing 1040 chemical compounds library screened on pollen cells.Additional file2:Table S2. Listing 1040 chemical compounds library screened on pollen cells.Additional file2:Table S2. Listing 1040 chemical compounds library screened on pollen cells.Additional file2:Table S2. Listing 1040 chemical compounds library screened on pollen cells.Additional file2:Table S2. Listing 1040 chemical compounds library screened on pollen cells.Additional file2:Table S2. Listing 1040 chemical compounds library screened on pollen cells.Additional file2:Table S2. Listing 1040 chemical compounds library screened on pollen cells.Additional file2:Table S2. Listing 1040 chemical compounds library screened on pollen cells.Additional file2:Table S2. Listing 1040 chemical compounds library screened on pollen cells.Additional file2:Table S2. Listing 1040 chemical compounds library screened on pollen cells.Additional file2:Table S2. Listing 1040 chemical compounds library screened on pollen cells.Additional file2:Table S2. Listing 1040 chemical compounds library screened on pollen cells.Additional file2:Table S2. Listing 1040 chemical compounds library screened on pollen cells.Additional file2:Table S2. Listing 1040 chemical compounds library screened on pollen cells.Additional file2:Table S2. Listing 1040 chemical compounds library screened on pollen cells.Additional file2:Table S2. Listing 1040 chemical compounds library screened on pollen cells.Additional file2:Table S2. Listing 1040 chemical compounds library screened on pollen cells.Additional file2:Table S2. Listing 1040 chemical compounds library screened on pollen cells.Additional file2:Table S2. Listing 1040 chemical compounds library screened on pollen cells.Additional file2:Table S2. Listing 1040 chemical compounds library screened on pollen cells.Additional file2:Table S2. Listing 1040 chemical compounds library screened on pollen cells.Additional file2:Table S2. Listing 1040 chemical compounds library screened on pollen cells.Additional file2:Table S2. Listing 1040 chemical compounds library screened on pollen cells.Additional file2:Table S2. Listing 1040 chemical compounds library screened on pollen cells.Additional file2:Table S2. Listing 1040 chemical compounds library screened on pollen cells.Additional file2:Table S2. Listing 1040 chemical compounds library screened on pollen cells.Additional file2:Table S2. Listing 1040 chemical compounds library screened on pollen cells.Additional file2:Table S2. Listing 1040 chemical compounds library screened on pollen cells.Additional file2:Table S2. Listing 1040 chemical compounds library screened on pollen cells.Additional file2:Table S2. Listing 1040 chemical compounds library screened on pollen cells.Additional file2:Table S2. Listing 1040 chemical compounds library screened on pollen cells.Additional file2:Table S2. Listing 1040 chemical compounds library screened on pollen cells.Additional file2:Table S2. Listing 1040 chemical compounds library screened on pollen cells.Additional file2:Table S2. Listing 1040 chemical compounds library screened on pollen cells.Additional file2:Table S2. Listing 1040 chemical compounds library screened on pollen cells.Additional file2:Table S2. Listing 1040 chemical compounds library screened on pollen cells.Additional file2:Table S2. Listing 1040 chemical compounds library screened on pollen cells.Additional file2:Table S2. Listing 1040 chemical compounds library screened on pollen cells.Additional file2:Table S2. Listing 1040 chemical compounds library screened on pollen cells.Additional file2:Table S2. Listing 1040 chemical compounds library screened on pollen cells.Additional file2:Table S2. Listing 1040 chemical compounds library screened on pollen cells.Additional file2:Table S2. Listing 1040 chemical compounds library screened on pollen cells.Additional file2:Table S2. Listing 1040 chemical compounds library screened on pollen cells.Additional file2:Table S2. Listing 1040 chemical compounds library screened on pollen cells.Additional file2:Table S2. Listing 1040 chemical compounds library screened on pollen cells.Additional file2:Table S2. Listing 1040 chemical compounds library screened on pollen cells.Additional file2:Table S2. Listing 1040 chemical compounds library screened on pollen cells.Additional file2:Table S2. Listing 1040 chemical compounds library screened on pollen cells.Additional file2:Table S2. Listing 1040 chemical compounds library screened on pollen cells.Additional file2:Table S2. Listing 1040 chemical compounds library screened on pollen cells.Additional file2:Table S2. Listing 1040 chemical compounds library screened on pollen cells.Additional file2:Table S2. Listing 1040 chemical compounds library screened on pollen cells.Additional file2:Table S2. Listing 1040 chemical compounds library screened on pollen cells.Additional file2:Table S2. Listing 1040 chemical compounds library screened on pollen cells.Additional file2:Table S2. Listing 1040 chemical compounds library screened on pollen cells.Additional file2:Table S2. Listing 1040 chemical compounds library screened on pollen cells.Additional file2:Table S2. Listing 1040 chemical compounds library screened on pollen cells.Additional file2:Table S2. Listing 1040 chemical compounds library screened on pollen cells.** |
| --- | --- |
| # | **SMILES** |
| 1 | ClC1(Cl)CC1(C(=O)Nc1ccc(OC)cc1)c1ccccc1 |
| 2 | O=C(N\N=C\c1ccccc1[N+](=O)[O-])C1C2C1CCC=CCC2 |
| 3 | O(C(=O)c1ccc(NC(=O)C(CC)(CC)C(OCC)=O)cc1)CC |
| 4 | S(=O)(=O)(NNC(=O)CC(=O)Nc1ccccc1C(O)=O)c1ccc([N+](=O)[O-])cc1 |
| 5 | s1c2c(CCCC2)c(C(=O)N)c1NC(=O)c1cc([N+](=O)[O-])ccc1 |
| 6 | OC(CNc1ccccc1)Cn1c2c(c3c1cccc3)cccc2 |
| 7 | OC(=O)c1ccccc1NC(=O)C1CCCCC1 |
| 8 | Clc1ccc(cc1)C(=O)c1ccc([N+](=O)[O-])cc1 |
| 9 | Brc1ccc(cc1)C(=O)Nc1cc2c(cc1)cccc2 |
| 10 | Brc1ccc(N(C(=O)C)CN2C(=O)c3c(ccc(Br)c3)C2=O)cc1 |
| 11 | Brc1cc2c(cc1)C(=O)N(c1ccccc1OC)C2=O |
| 12 | O=C1N(c2ccccc2C)C(=O)c2c1cc(cc2)C(=O)c1cc2c(cc1)C(=O)N(c1ccccc1C)C2=O |
| 13 | Brc1cc2c(cc1)C(=O)N(C2=O)c1ccc(cc1)C(OCCC(C)C)=O |
| 14 | O=C1N(C(=O)c2c1cc(cc2)C(O)=O)c1ccc(cc1)C(OCCC(C)C)=O |
| 15 | O=C1N(C(=O)c2c1cc(cc2)C(=O)c1cc2c(cc1)C(=O)N(C2=O)c1ccc(cc1)C(OCC(C)C)=O)c1ccc(cc1)C(OCC(C)C)=O |
| 16 | O=C1N(C(=O)c2c1c([N+](=O)[O-])ccc2)c1ccc(cc1)C(OCC(C)C)=O |
| 17 | O=C1N(C(=O)C2C1C1c3c(C2c2c1cccc2)cccc3)c1ccc(cc1)C(O)=O |
| 18 | O(C(=O)c1ccccc1-c1ccccc1)CC(=O)c1ccccc1 |
| 19 | O1CCN(CC1)CN1C(=O)c2c(cccc2)C1=O |
| 20 | O=C1N(Cc2ccccc2)C(=O)c2c1cc(cc2)C(O)=O |
| 21 | ClC(Cl)(Cl)C(c1cc([N+](=O)[O-])c(OC)c([N+](=O)[O-])c1)c1cc([N+](=O)[O-])c(OC)c([N+](=O)[O-])c1 |
| 22 | O(C(=O)c1ccccc1)c1ccc(cc1)C(=O)c1ccc(OC(=O)c2ccccc2)cc1 |
| 23 | Clc1ccccc1C(=O)Nc1ccc(Oc2cc(Oc3ccc(NC(=O)c4ccccc4Cl)cc3)ccc2)cc1 |
| 24 | O(CC(=O)Nc1cc(ccc1)C(=O)N)c1ccc(cc1C(CC)(C)C)C(CC)(C)C |
| 25 | Brc1cc([N+](=O)[O-])ccc1NC(=O)c1cc(ccc1)C(=O)Nc1ccc([N+](=O)[O-])cc1Br |
| 26 | O(CCCC)c1ccc(cc1)C(=O)NNC(=O)c1ccc(cc1)CCC |
| 27 | s1c(nnc1-c1ccc(cc1)CC)-c1ccc(cc1)CCCCCCC |
| 28 | o1c(nnc1-c1ccc(OCCCC)cc1)-c1ccc([N+](=O)[O-])cc1 |
| 29 | O(c1ccc(NC(=O)c2ccc(N)cc2)cc1)c1ccc(NC(=O)c2ccc(N)cc2)cc1 |
| 30 | O(C(=O)c1ccc(cc1)C(=O)NCCCc1[nH]c2c(n1)cccc2)C |
| 31 | O1CCN(CC1)Cc1[nH]c2c(n1)cc([N+](=O)[O-])cc2 |
| 32 | S(=O)(=O)(Nc1ccc(cc1)C(O)=O)\C=C\c1ccccc1 |
| 33 | N1=C2c3c(N=C2N(c2c1cccc2)C)cccc3 |
| 34 | OC(=O)c1nccnc1C(=O)Nc1ccc([N+](=O)[O-])cc1 |
| 35 | Oc1cc(N2C(=O)c3nccnc3C2=O)ccc1 |
| 36 | O(C)c1ccccc1NC(=O)CC1Nc2c(NC1=O)cccc2 |
| 37 | S=P(OC)(Nc1c(cc(cc1C(C)(C)C)C(C)(C)C)C(C)(C)C)c1c2c(cccc2N(C)C)ccc1 |
| 38 | O=C(Nc1c(cc(cc1C)C)C)C12CC3CC(C1)CC(C2)C3 |
| 39 | Brc1cc2c(NC(=O)C(C(=O)\C=C\c3cc4c(nc3Cl)cc(cc4)C)=C2c2ccccc2)cc1 |
| 40 | ClC(Cl)(Cl)C(NC(=O)Cc1cc(OC)cc(OC)c1)NC(=S)Nc1ccc(cc1)C(OC)=O |
| 41 | Brc1cc(NC(=S)NC(NC(=O)c2occc2)C(Cl)(Cl)Cl)ccc1 |
| 42 | Oc1ccccc1\C=N\Nc1nc2[nH]c3c(c2nn1)cccc3 |
| 43 | Brc1cc2c3nnc(nc3[nH]c2cc1)N\N=C\c1ccc(OCC)cc1O |
| 44 | c1c2Cc3cc(ccc3-c2ccc1\C=C\c1ccccc1)\C=C\c1ccccc1 |
| 45 | s1cc(nc1/C(=C\c1ccc(O)cc1)/C#N)-c1ccccc1 |
| 46 | S(CC(=O)N)C=1NC(C)=C(C(=O)Nc2ccccc2OC)C(C=1C#N)c1occc1 |
| 47 | S(Cc1ccccc1)C=1NC(=O)C=C(C)C=1C#N |
| 48 | O1c2c(C=C(C(NNC(=O)C)=N)C1=O)cccc2 |
| 49 | Clc1ccc(cc1)C(=O)\C=C\c1occc1 |
| 50 | Oc1cc(N(CC)CC)ccc1\C=N\NC(=O)c1ccc([N+](=O)[O-])cc1 |
| 51 | O1c2cc(O)c(cc2C=C(/C(=N/NC(=O)c2cc3c(cc2O)cccc3)/C)C1=O)CCCCCC |
| 52 | Clc1cc(C(OC)=O)c(Cl)cc1C(OC)=O |
| 53 | O=C/1/C(/CCC\C\1=C/c1ccc([N+](=O)[O-])cc1)=C/c1ccc(cc1)C |
| 54 | O=C/1/C(/CCC\C\1=C/c1ccc(cc1)C)=C/c1ccc(cc1)C |
| 55 | Brc1ccc(cc1)\C=C\1/CCC\C(=C/c2ccc(Br)cc2)\C/1=O |
| 56 | OC(=O)CCCCCNC(=O)C |
| 57 | Clc1cc2c(NC(=O)C(C(=O)\C=C\C3=Cc4c(NC3=O)cc(OCC)cc4)=C2c2ccccc2)cc1 |
| 58 | Brc1ccc(cc1)C(=O)\C=C\c1oc(N2CCOCC2)cc1 |
| 59 | o1c(ccc1\C=C(\C(=O)Nc1ccc(cc1C)C)/C#N)-c1cc([N+](=O)[O-])ccc1 |
| 60 | O(C)c1cc(NC(=O)\C(=C/c2ccc(OC)cc2)\C#N)ccc1 |
| 61 | O(CC)c1ccc(NC(=O)\C(=C/c2ccc(OC)cc2)\C#N)cc1 |
| 62 | S(=O)(=O)(N1CCOCC1)c1ccc(cc1)C(=O)Nc1cc2c(cc1)C(=O)N(C)C2=O |
| 63 | S(=O)(=O)(N1CCCCC1)c1ccc(cc1)C(=O)Nc1cc([N+](=O)[O-])ccc1 |
| 64 | S(=O)(=O)(N1CCOCC1)c1ccc(cc1)C(=O)Nc1ccc(cc1OC)C |
| 65 | S(=O)(=O)(Nc1nccnc1OC)c1ccc(NC(=O)C23CC4CC(C2)CC(C3)C4)cc1 |
| 66 | S(=O)(=O)(Nc1nccnc1OC)c1ccc(NC(=O)c2cc(ccc2)C)cc1 |
| 67 | O=[N+]([O-])c1ccc(N\N=C\2/N(N)C(=Nc3c/2cccc3)C)cc1 |
| 68 | O1CCN(CC1)c1c([N+](=O)[O-])c(N2CCOCC2)c([N+](=O)[O-])cc1C(=O)N1CC(=O)Nc2c1cccc2 |
| 69 | Brc1ccccc1C(=O)NCC(=O)N\N=C(\C)/C |
| 70 | S1c2c(OC1=O)cc(OC(OC)=O)cc2 |
| 71 | Brc1cc2OC(=O)N(c2cc1)C(=O)c1ccc(Cl)cc1 |
| 72 | Clc1cc(Cl)ccc1Oc1cc(C(O)=O)c([N+](=O)[O-])cc1C(O)=O |
| 73 | Clc1cc(S(=O)(=O)c2cc(S(=O)(=O)Nc3cc(ccc3)C(O)=O)ccc2)ccc1Cl |
| 74 | S(=O)(=O)(Nc1cc([N+](=O)[O-])ccc1)c1cc(ccc1)C(OC)=O |
| 75 | S(Oc1ccc([N+](=O)[O-])cc1)(=O)(=O)c1ccc(Oc2ccccc2)cc1 |
| 76 | o1cccc1C(=O)Nc1cc(ccc1)C(OCC(=O)c1ccccc1)=O |
| 77 | O=C1N(CNc2ccc(cc2)C)C(=O)c2c1cccc2 |
| 78 | O(c1cccc(C)c1C)c1ccc(N2C(=O)c3c(cccc3)C2=O)cc1 |
| 79 | O(c1cc([N+](=O)[O-])ccc1)c1cc2c(cc1)C(=O)N(c1ccccc1C(O)=O)C2=O |
| 80 | O(c1cc(C)c(cc1)C)c1ccc(N2C(=O)c3c(ccc(Oc4cc([N+](=O)[O-])ccc4)c3)C2=O)cc1 |
| 81 | O(CCOc1ccc(cc1)C#N)c1ccc(cc1)C(=O)C |
| 82 | O(CCOc1ccc([N+](=O)[O-])cc1)c1ccc(cc1)C(=O)C |
| 83 | O(CC(C)C)c1ccc(cc1)C(=O)c1ccc(OC)cc1 |
| 84 | O(CCCCCCCCC)c1ccc(cc1)\C(=N\O)\c1ccc(OC)cc1 |
| 85 | S=C(\N=C(\N(CC)CC)/C)Nc1ccccc1 |
| 86 | O1C(CO)C(O)C(O)C(O)C1Oc1cc(O)c(cc1)C(=O)Cc1ccccc1 |
| 87 | Brc1c2cc(OC)ccc2sc1C(O)=O |
| 88 | o1cccc1\C=C\1/CC\C(=C/c2occc2)\C/1=O |
| 89 | BrC=1C=CC2NC(=O)CN(C(OCc3ccccc3)=O)C(=C2C=1)c1ccccc1 |
| 90 | Brc1cc(C(N(S(=O)(=O)c2ccc(cc2)C)CC(O)=O)c2ccccc2)c(NS(=O)(=O)c2ccc(cc2)C)cc1 |
| 91 | OC(c1cc(ccc1N)C)c1ccccc1 |
| 92 | O(C)c1c(OC)cc(cc1OC)\C=N\c1ccc(cc1C(NCC(OC)=O)c1ccccc1)C |
| 93 | Oc1ccc(cc1)\C=N\NC(=O)C12CC3(CC(C1)(CC(C2)C3)C)C |
| 94 | O=C(N\N=C\c1ccc([N+](=O)[O-])cc1)C12CC3(CC(C1)(CC(C2)C3)C)C |
| 95 | O=C(N\N=C\c1cc([N+](=O)[O-])ccc1)C12CC3CC(C1)CCC(C2)C3 |
| 96 | Oc1ccccc1\C=N\NC(=O)NC12CC3(CC(C1)CC(C3)C2)c1ccc(cc1)C |
| 97 | O=C(N\N=C\c1cc([N+](=O)[O-])ccc1)C12CC3(CC(C1)CC(C3)C2)c1ccc(cc1)C |
| 98 | O(C)c1ccc(cc1N)C12CC3CC(C1)CC(C2)C3 |
| 99 | O=C(N\N=C\c1cc([N+](=O)[O-])ccc1)C12CC3CC(C1)CC(C2)C3 |
| 100 | Brc1ccc(cc1)\C=N\C(C(OC)=O)C12CC3CC(C1)CC(C2)C3 |
| 101 | O=C(\C=C\c1cccnc1)c1ccc(N(C)C)cc1 |
| 102 | s1cccc1C(=O)\C=C\c1cc2c3c(n(c2cc1)C)cccc3 |
| 103 | s1cc(nc1N1N=C\2C(CCC/C/2=C\c2ccc(N(C)C)cc2)C1c1ccc(N(C)C)cc1)-c1ccccc1 |
| 104 | s1cc(nc1N\N=C\c1ccccc1)-c1ccccc1 |
| 105 | S=C1NC(C2=C(N1)/C(/CCC2)=C/c1ccc(N(C)C)cc1)c1ccc(N(C)C)cc1 |
| 106 | s1cc(nc1N)-c1ccccc1 |
| 107 | O1c2c(C=C(/C(=N/NC(=O)c3ccccc3)/C)C1=O)cccc2 |
| 108 | Oc1cc(O)c(cc1CCCCCC)\C=N\NC(=O)c1ccccc1 |
| 109 | O(C)c1ccc(cc1)-c1ccc(cc1)\C=C\1/C(=O)C(CCC/1C)C(C)C |
| 110 | O=C(CNc1ccc(cc1)C)c1ccc([N+](=O)[O-])cc1 |
| 111 | Brc1ccc(cc1)C(=O)\C=C\c1ccc(F)cc1 |
| 112 | O=[N+]([O-])CC(Nc1ccccc1)c1ccccc1 |
| 113 | O=C(N\N=C\c1ccccc1)CCc1c2c([nH]c1)cccc2 |
| 114 | O(C)c1cc2[nH]c(C(OCC)=O)c(c2cc1)CCCN1C(=O)c2c(cccc2)C1=O |
| 115 | Clc1cc(NC(=S)NC(OC)=O)ccc1Oc1ccc(Cl)cc1 |
| 116 | Clc1cc(NC(=O)c2cc(Cl)ccc2O)ccc1Oc1cc2nsnc2cc1 |
| 117 | O=C(NCC1NCCc2c1cccc2)C1CCCCC1 |
| 118 | s1c2c(CCCC2)c(C#N)c1NC(=O)CCC(O)=O |
| 119 | o1c(c(-c2ccccc2)c(C#N)c1\N=C\c1cccc(CC=C)c1O)-c1ccccc1 |
| 120 | s1c(C)c(C)c(C#N)c1\N=C\c1cc(OC)ccc1O |
| 121 | s1c2c(CCCC2)c(C#N)c1\N=C\c1ccc(N(C)C)cc1 |
| 122 | Brc1ccc(cc1)C(=O)COC(=O)C1=Cc2c(OC1=O)c(ccc2)CC=C |
| 123 | Brc1ccc(cc1)C(=O)COC(=O)c1ccc(Br)cc1 |
| 124 | Brc1cc(C(=O)Nc2ccc(cc2)-c2ccc(NC(=O)c3cc(Br)ccc3O)cc2)c(O)cc1 |
| 125 | O(C(=O)c1c2[nH]cc(c2ccc1)\C=C\[N+](=O)[O-])C |
| 126 | Brc1cc(ccc1)C(Oc1ccc(cc1)\C=N\c1cc2nc(oc2cc1)-c1cc2c(cc1)cccc2)=O |
| 127 | Brc1cc(ccc1)\C=N\c1cc(-c2oc3c(n2)cc(cc3)C)c(O)cc1 |
| 128 | Brc1cc(ccc1OC)-c1oc2c(n1)cc(\N=C\c1cc(Cl)ccc1)cc2 |
| 129 | Clc1ccccc1N=Nc1c(nn(c1O)-c1ccccc1)C |
| 130 | O1C(COC(=O)C)C(OC(=O)C)C(OC(=O)C)C(NC(=O)C)C1OCCCCCCCCCCCCCCCC |
| 131 | O1C(COC(=O)C)C(OC(=O)C)C(OC(=O)C)C(NC(=O)C)C1OCCCCCCCC |
| 132 | Clc1cc2c(nc(C)c(C(=O)\C=C\c3cc(OC)c(OC)c(OC)c3)c2-c2ccccc2)cc1 |
| 133 | O1c2cc(ccc2OC1)\C=N\NC1=Nc2c(cccc2)C(=O)N1c1ccccc1 |
| 134 | Clc1nc2cc(OCC)ccc2cc1\C=C\C(=O)c1ccc(OC)cc1 |
| 135 | Clc1cc2c(nc3n(nc(c3c2-c2ccccc2)C)C(=O)C)cc1 |
| 136 | O(CCOCCOc1ccccc1O)c1ccccc1O |
| 137 | S(Oc1ccc(cc1)C)(=O)(=O)N1CCOCCOCCOCC1 |
| 138 | O(C)c1cc(ccc1OC)\C=C\c1cc(OC)c(OC)cc1 |
| 139 | s1c2c(nc1-c1ccccc1)cccc2 |
| 140 | S1C(\C=N\NC(=S)N)=C([n+]2ccc(cc2)C)[N-]C1=O |
| 141 | O1C(=N\C(=C\2/C=C(OC(=C/2)C)C)\C1=O)c1ccccc1 |
| 142 | O=C1CCCc2c1n(c1c2cccc1)C |
| 143 | o1cccc1\C=N\N(c1ccccc1)c1ccccc1 |
| 144 | O=C(Nc1c(n(nc1C)C(=O)C)C)C |
| 145 | O1c2c(cccc2)C(c2c1cccc2)C1C(=O)c2c(cccc2)C1=O |
| 146 | S(=O)(=O)(NC1CCCCC1)c1ccc(NC(=O)c2ccc([N+](=O)[O-])cc2)cc1 |
| 147 | O=C(Nc1cc([N+](=O)[O-])ccc1)C(c1ccccc1)c1ccccc1 |
| 148 | S(=O)(=O)(Nc1cc(F)ccc1)c1ccc(cc1C)C(=O)Nc1ccccc1C |
| 149 | S(Oc1ccc([N+](=O)[O-])cc1)(=O)(=O)c1cc(ccc1)C(=O)Nc1ccccc1 |
| 150 | Clc1ccc(OCc2ccc(cc2)C(=O)Nc2ccccc2)cc1 |
| 151 | O=C1NC(=O)NC(=O)C1[N+](=O)[O-] |
| 152 | O=C(Nc1ccc([N+](=O)[O-])cc1)Cc1ccccc1 |
| 153 | Brc1ccc(cc1)C(=O)NCC(O)=O |
| 154 | O(C(=O)c1ccc([N+](=O)[O-])cc1)c1cc2c(cc1)cccc2 |
| 155 | Clc1ccc(OC(=O)c2ccc([N+](=O)[O-])cc2)cc1 |
| 156 | S=C(NC(=O)\C=C\c1ccccc1)N |
| 157 | OC(=O)c1nccnc1C(=O)NC1CCCCC1 |
| 158 | OC(=O)c1nccnc1C(=O)Nc1ccc(NC(=O)c2nccnc2C(O)=O)cc1 |
| 159 | OC(=O)c1nccnc1C(=O)NC(C)(C)C |
| 160 | OC(=O)\C=C\C=1CCCCC=1C#N |
| 161 | O(c1cc(NC(=O)C)ccc1)c1cc2c(cc1)C(=O)N(C2=O)c1cc(NC(=O)C)ccc1 |
| 162 | Brc1ccc(cc1)-c1nc([nH]c1-c1ccc(Br)cc1)-c1ccccc1 |
| 163 | O=[N+]([O-])c1ccc(cc1)C#Cc1ccc(cc1)-c1nc2c(nc1-c1ccc(cc1)C#Cc1ccc([N+](=O)[O-])cc1)cccc2 |
| 164 | O=C(N)C12CC3(CC(C1)(CC(C2)(C3)C)C)C |
| 165 | O=C(Nc1cc(ccc1C)C12CC3CC(C1)CC(C2)C3)Nc1ccccc1 |
| 166 | s1c2cc(ccc2nc1-c1ccc(NC(=O)\C=C\c2ccc(F)cc2)cc1)C |
| 167 | s1c2c(nc1-c1ccc(NC(=O)\C=C\c3ccc([N+](=O)[O-])cc3)cc1)cccc2 |
| 168 | O(C)c1ccc(Nc2ccccc2)cc1 |
| 169 | Clc1cc(N)c(S(=O)(=O)Nc2ccccc2C)cc1 |
| 170 | S1(=O)(=O)Nc2c(-c3c1cccc3)c(C)c([N+](=O)[O-])cc2C |
| 171 | s1cc(nc1N(CCC(=O)N)CCC(=O)N)C |
| 172 | S(=O)(=O)(N(CCC(O)=O)CCC(O)=O)c1ccc(cc1)C |
| 173 | OC(=O)C1C2CC(C=C2)C1C(=O)N(Cc1ccccc1)C |
| 174 | P(OCC)(OCC)(=O)C(Nc1ccc([N+](=O)[O-])cc1)c1ccccc1 |
| 175 | S=P1(OCC(CO1)(C)C)C(Nc1ccccc1)c1ccc(OCC)cc1 |
| 176 | O=[N+]([O-])c1cc([N+](=O)[O-])ccc1N\N=C(/CCCC)\CC |
| 177 | Clc1ccc(N\N=C/2\C(=O)NC(=O)NC\2=O)cc1 |
| 178 | Clc1cc(C(=O)c2ccccc2)c(NC(=O)C(Cl)(Cl)Cl)cc1 |
| 179 | S(Oc1cc(ccc1)C=O)(=O)(=O)c1ccc(cc1)C |
| 180 | O=Cc1nc2c(nc1)cccc2 |
| 181 | s1c2c(CCCC2)c(C(OCC)=O)c1NC(=O)c1ccccc1 |
| 182 | o1cccc1C(OCC(=O)c1ccccc1)=O |
| 183 | Brc1ccc(NC(=O)COc2ccc(Cl)cc2)cc1 |
| 184 | Clc1ccc(NC(NC(=O)Cc2ccccc2)C(Cl)(Cl)Cl)cc1 |
| 185 | Brc1ccc(cc1)C(=O)NC(Nc1ccccc1C)C(Cl)(Cl)Cl |
| 186 | ClC(Cl)(Cl)C(NC(C)(C)C)NC(=O)Cc1ccccc1 |
| 187 | Clc1ccc(NC(=S)NC(NC(=O)C(C)C)C(Cl)(Cl)Cl)cc1 |
| 188 | ClC(Cl)(Cl)C(NC(=O)c1cc([N+](=O)[O-])ccc1)NC(=S)Nc1cc([N+](=O)[O-])ccc1 |
| 189 | Clc1cc(Cl)ccc1NC(=S)NC(NC(=O)c1cc(Cl)ccc1)C(Cl)(Cl)Cl |
| 190 | Clc1cc(Cl)cc(Cl)c1OC(NC(=O)\C=C\c1ccccc1)C(Cl)(Cl)Cl |
| 191 | S(=O)(=O)(NCC(O)=O)c1ccc(cc1)C |
| 192 | O=C1c2c(N(Cc3ccc(cc3)Cc3ccc(cc3)CN3c4c(cccc4)C(=O)C3=O)C1=O)cccc2 |
| 193 | S(O)(=O)(=O)c1cc([N+](=O)[O-])cc(N)c1O |
| 194 | S=C(Nc1ccc(cc1)C)N\N=C/1\C2CCC(C\1=O)C2(C)C |
| 195 | O(C)c1ccc(cc1)C1(CCCC1)C(O)=O |
| 196 | O(C)c1ccc(NC(=O)c2ccc(cc2)-c2ncc(cc2)CC)cc1 |
| 197 | Brc1cc2c(cc(OC(=O)c3ccc(cc3)-c3ncc(cc3)CC)cc2)cc1 |
| 198 | O(C(=O)c1ccc(cc1)-c1ncc(cc1)CCCCC)c1cc(NC(=O)c2ccc(cc2)-c2ncc(cc2)CCCCC)ccc1 |
| 199 | Brc1cc(cnc1)C(Oc1ccc(cc1)C(OCCCC)=O)=O |
| 200 | Brc1cc(cnc1)C(=O)Nc1ccc(cc1[N+](=O)[O-])C |
| 201 | Brc1cc(cnc1)C(Oc1ccc(cc1)C(OCCCCC)=O)=O |
| 202 | O(C(=O)c1nc(ccc1)C(OC)=O)c1ccc(cc1)C(OCCCCCCC)=O |
| 203 | O=C(Nc1ccc(cc1C)C)c1cccnc1 |
| 204 | O=C(Nc1ccc(cc1[N+](=O)[O-])C)c1cccnc1 |
| 205 | O(C(=O)c1cccnc1)c1ccc(cc1)C(OCCCCCC)=O |
| 206 | S(=O)(=O)(c1ccc(OC(=O)c2nc(ccc2)C(OCC)=O)cc1)c1ccc(OC(=O)c2nc(ccc2)C(OCC)=O)cc1 |
| 207 | O(C(=O)c1nc(ccc1)C(=O)Nc1nc(NC(=O)c2nc(ccc2)C(OC(C)C)=O)ccc1)C(C)C |
| 208 | O(C(=O)c1nc(ccc1)C(OC(C)C)=O)c1ccc(OCCCCCC)cc1 |
| 209 | O(C(=O)c1ccc(nc1)C(Oc1ccc(OCC)cc1)=O)c1ccc(OCC)cc1 |
| 210 | O(C(=O)c1ccc(nc1)C(Oc1ccc(OCCCC)cc1)=O)c1ccc(OCCCC)cc1 |
| 211 | s1cccc1C(=O)Nc1ncccn1 |
| 212 | s1cccc1C(Oc1cc(OC(=O)c2sccc2)ccc1)=O |
| 213 | O=C(NNC(=O)Cc1ccc(cc1)CCC)C1CCC(CC1)c1ccccc1 |
| 214 | O=C1CC(CC(NCCCC)=C1)(C)C |
| 215 | O=C(N\N=C/1\CCCc2c\1cccc2)CC#N |
| 216 | O=C(N\N=C\c1c2c([nH]c1)cccc2)C1CC1C(=O)N\N=C\c1c2c([nH]c1)cccc2 |
| 217 | Brc1cc([N+](=O)[O-])cc(\C=N\c2cc(ccc2O)C23CC4CC(C2)CC(C3)C4)c1O |
| 218 | Fc1cc(NC(=O)COc2cc(C)c(cc2)C)ccc1 |
| 219 | Brc1ccc(NS(=O)(=O)c2cc(ccc2)C(OC)=O)cc1 |
| 220 | S(Oc1ccc(N)cc1)(=O)(=O)c1cc(ccc1)C(=O)Nc1cc(N)ccc1 |
| 221 | Clc1ccc(OC(=O)C2CC(CCC2)C(Oc2ccc(Cl)cc2)=O)cc1 |
| 222 | S(=O)(=O)(c1ccc(-n2c(ccc2C)C)cc1)c1ccc(-n2c(ccc2C)C)cc1 |
| 223 | O(C(=O)c1ccc([N+](=O)[O-])cc1[N+](=O)[O-])c1ccc(cc1C)-c1cc(ccc1)C |
| 224 | O(C(=O)c1ccccc1OC)c1ccccc1-c1ccccc1OC(=O)c1ccccc1OC |
| 225 | S(=O)(=O)(c1ccc(OC(=O)c2ccc(F)cc2)cc1)c1ccc(OC(=O)c2ccc(F)cc2)cc1 |
| 226 | S1C(N(N=C1NC(=O)C)C(=O)C)(CCS(=O)(=O)c1ccc(cc1)C)C |
| 227 | Clc1cc(S(=O)(=O)C(CC(=O)C)(C)C)ccc1Cl |
| 228 | O(C(=O)c1ccc(cc1)C(OCc1ccc([N+](=O)[O-])cc1[N+](=O)[O-])=O)Cc1ccc([N+](=O)[O-])cc1[N+](=O)[O-] |
| 229 | O(c1ccc(cc1)-c1ccccc1)c1ccc([N+](=O)[O-])cc1 |
| 230 | Clc1cc(Cl)ccc1\C=N\c1cc(Cl)c(NC(=O)c2ccc(\N=C\c3ccc(Cl)cc3Cl)cc2)cc1 |
| 231 | n1c2n(c3c(c2nnc1-n1nc(cc1C)C)cccc3)CC |
| 232 | Fc1ccc(cc1)\C=N\Nc1nc2n(c3c(c2nn1)cccc3)CC |
| 233 | S(=O)(=O)([O-])CCC[n+]1c2c(cc(C)c(c2)C)c(cc1)C |
| 234 | O1CCN(CC1)C(=O)c1ccc(cc1)C(OC)=O |
| 235 | o1c2c(nc1-c1ccc(NC(=O)C3CCCCC3)cc1)cc(NC(=O)C1CCCCC1)cc2 |
| 236 | o1c2c(nc1CCc1ccc(N)cc1)cccc2 |
| 237 | O=[N+]([O-])c1cc2nc([nH]c2cc1)/C(=C/c1ccccc1)/C |
| 238 | Clc1ccc(nc1)NS(=O)(=O)c1ccccc1 |
| 239 | O1C(CO)C(O)C(O)C1n1c2N=CNC(=O)c2nc1NCc1ccccc1 |
| 240 | O=C1NC(=Nc2[nH]c(nc12)N1CCN(CC1)C)N |
| 241 | S(CCN1CCOCC1)c1[nH]c2c(n1)cccc2 |
| 242 | O(CCCC)c1c2N(C)C(=O)C(=Nc2c(OCCCC)cc1)c1ccccc1N |
| 243 | o1c2c(nc1-c1cc(NC(=O)c3ccc([N+](=O)[O-])cc3[N+](=O)[O-])c(cc1)C)cc(cc2C)C |
| 244 | s1cc(nc1/C(=C/c1ccc(OC(=O)C)cc1)/C#N)C1=Cc2c(OC1=O)cccc2 |
| 245 | O(C)c1ccc(cc1)C1C2=C(NC(C)=C1C(OC)=O)CCCC2=O |
| 246 | O=C1N(C(=Nc2c1cccc2)C)c1cc(ccc1)C |
| 247 | Clc1cc(ccc1Cl)-c1nc(sc1)NC(=O)c1ccc([N+](=O)[O-])cc1 |
| 248 | s1c2c(nc1NC(=O)c1ccc(F)cc1)c(cc(c2)C)C |
| 249 | o1c2c(cc(OC(=O)c3ccccc3OC)c3c2cccc3)c(C(OCC)=O)c1-c1ccccc1 |
| 250 | S1\C(=C\c2ccc(F)cc2)\C(=O)N(CN(C)C)C1=O |
| 251 | S1\C(=C\c2ccc(OC)cc2)\C(=O)N(CNc2ccc(F)cc2)C1=S |
| 252 | O(c1ccc([N+](=O)[O-])cc1[N+](=O)[O-])c1ccc(cc1OCC)\C=N\Nc1nc(nc(n1)N1CCCCC1)N1CCCCC1 |
| 253 | S1\C(=C\c2cc([N+](=O)[O-])c(O)c(OC)c2)\C(=O)NC1=O |
| 254 | S(=O)(=O)(N1CCCCC1)c1cc(ccc1)C(=O)Nc1ncccc1 |
| 255 | s1c2cc(NC(=O)c3ccc(cc3)C(OC)=O)ccc2nc1NC(=O)C |
| 256 | S\1\C(=C\c2oc(cc2)-c2ccc(cc2)C(O)=O)\C(=O)N(/C/1=N/c1ccccc1)c1ccccc1 |
| 257 | Ic1ccccc1C(=O)Nc1sc(nn1)CCOCCOCCc1sc(nn1)NC(=O)c1ccccc1I |
| 258 | Brc1ccc(cc1)\C=N\NC(=O)Cc1sc(nn1)NC(=O)c1ccc(cc1)C |
| 259 | Brc1cc(\C=N/NC(=O)Cc2sc(nn2)NC(=O)c2ccc(OC)cc2)c(O)cc1 |
| 260 | Clc1cc(Cl)ccc1\C=N\NC(=O)Cc1sc(nn1)NC(=O)CC |
| 261 | Clc1cc(Cl)ccc1COc1ccc(cc1OC)\C=N\Nc1nc(nc(n1)Nc1ccccc1)N1CCOCC1 |
| 262 | Oc1ccc(N2C(=O)c3c4c(cccc4c([N+](=O)[O-])cc3)C2=O)cc1 |
| 263 | O=C(Nc1ccc(cc1)-c1nc(c2cc(NC(=O)C)ccc2n1)-c1ccccc1)C |
| 264 | S(CCOc1ccccc1OC)c1nc2c(n1CC(O)=O)cccc2 |
| 265 | Ic1ccccc1C(=O)Nc1cc2nc(oc2cc1)-c1ccc(cc1)CC |
| 266 | O\1c2c(C=C(C(=O)N)/C/1=N/c1cc(ccc1)C(=O)N)cc(OC)cc2 |
| 267 | Brc1cc2C=C(C(=O)N)/C(/Oc2cc1)=N/c1ccc(F)cc1 |
| 268 | Clc1cc(Cl)cc2C=C(C(=O)Nc3ccc(F)cc3)C(Oc12)=O |
| 269 | S(CC(OCC)=O)c1nc2c(n1C)cccc2 |
| 270 | s1cc(nc1NC(=O)c1occc1)-c1ccc(cc1)C(C)C |
| 271 | s1c2c(nc1NC(=O)c1ccc(cc1)C(=O)C)c1c(cc2)cccc1 |
| 272 | O1CCN(CC1)c1c2c3c(cc1)C(=O)N(CCOC(=O)c1ccccc1)C(=O)c3ccc2 |
| 273 | Oc1ccc([N+](=O)[O-])cc1C1=NNC(C1)c1ccncc1 |
| 274 | O=C1c2c(C(=O)c3c1cccc3)c(N(C(=O)C)C)ccc2Nc1ccccc1 |
| 275 | Ic1cc(I)cc(\C=C/2\SC(=S)N(CC(O)=O)C\2=O)c1O |
| 276 | o1c2c(nc1-c1ccc(NC(=O)c3cc([N+](=O)[O-])c(cc3)C)cc1)cc(cc2)C |
| 277 | Clc1ccc(S(=O)(=O)N2CCOCC2)cc1C(=O)Nc1ccccc1O |
| 278 | S(CCOc1ccc(cc1)CC)c1nc2c(n1CC(O)=O)cccc2 |
| 279 | O=C\1N(c2ccccc2)C(=O)NC(=O)/C/1=C/c1c2c(n(c1)C(=O)C)cccc2 |
| 280 | Clc1cc(Cl)ccc1-c1oc2c(n1)cc(NC(=O)c1ccccc1F)cc2 |
| 281 | O=C1N(N(C)C(C)=C1\C=C/1\C(=O)NC(=O)NC\1=O)c1ccccc1 |
| 282 | S1/C(=C\C=C(\C=C/2\Sc3c4c(ccc3N\2CC)cccc4)/c2ccccc2)/C(=O)N(CC)C1=S |
| 283 | Ic1cc(ccc1)C(=O)Nc1cc2nc(oc2cc1)-c1ccccc1F |
| 284 | O(C)c1cc(OC)ccc1\C=C/1\C(=O)N(C)C(=O)N(C)C\1=O |
| 285 | O1c2c(OCC1C(=O)N\N=C\c1c3c(ccc1O)cccc3)cccc2 |
| 286 | Brc1cc2C=C(C(=O)NC(=O)C)/C(/Oc2cc1)=N/c1ccc(cc1)C(F)(F)F |
| 287 | Brc1cc2C=C(C(=O)Nc3ccccc3)/C(/Oc2cc1)=N/c1ccc(F)cc1 |
| 288 | s1cc(nc1N)C1=Cc2cc(OC)ccc2OC1=O |
| 289 | O=C1c2c3c(ncnc3ccc2N)-c2c1cccc2 |
| 290 | O1C2=C(C(C(C#N)=C1N)c1ccccc1)C(=O)CC(C2)(C)C |
| 291 | s1cc(nc1Nc1ccc(F)cc1)C1=Cc2cc(CCCCCC)c(O)cc2OC1=O |
| 292 | O\1c2c(C=C(C(=O)Nc3ccccc3)/C/1=N\NC(=O)c1ccccc1)cc(OC)cc2 |
| 293 | S1CC(=O)N(Cc2ccccc2)C1c1cc(OC)c(OC)c(OC)c1 |
| 294 | Clc1cc2c(OC=C(C(=O)c3ccccc3C)C2=O)cc1 |
| 295 | S1\C(=C\c2cc(ccc2)C)\C(=O)N(CN2CCCCC2)C1=O |
| 296 | Clc1ccccc1COc1ccc(cc1)\C=C/1\SC(=S)N(C\1=O)c1ccccc1 |
| 297 | O(C)c1ccc(NCN2C(=O)C3C(CCCC3)C2=O)cc1 |
| 298 | O=C1N(CNc2cc(ccc2C)C)C(=O)C2C1CCCC2 |
| 299 | Brc1c2c(oc(C=O)c2C(OCC)=O)c(cc1OC(=O)c1ccccc1Cl)C |
| 300 | Brc1cc(ccc1)Cn1c2c(nc1N\N=C\c1ccc(OC)cc1)N(C)C(=O)NC2=O |
| 301 | ClCCN(S(=O)(=O)c1ccc(cc1)C(=O)Nc1scc(n1)-c1cc(C)c(cc1)C)CCCl |
| 302 | s1c2cc(ccc2nc1NC(=O)c1ccc(Oc2ccccc2)cc1)C |
| 303 | Brc1cc2c(nc(nc2-c2ccccc2)Nc2ccc(cc2)C(=O)C)cc1 |
| 304 | S1\C(=C/c2ccc(OC)cc2)\C(=O)N(c2ccc(cc2)C)C1=S |
| 305 | O=C1N(Nc2ccc([N+](=O)[O-])cc2[N+](=O)[O-])C(=O)C2C1C1CC2C=C1 |
| 306 | Clc1ccccc1Cc1sc(nc1)NC(=O)c1occc1 |
| 307 | S1\C(=C/c2ccccc2)\C(=O)N(c2ccc(OCC)cc2)C1=S |
| 308 | S1\C(=C\c2oc(cc2)-c2ccc([N+](=O)[O-])cc2)\C(=O)N(C2CCS(=O)(=O)C2)C1=S |
| 309 | Brc1cc\2c(NC(=O)/C/2=C/2\SC(=S)N(Cc3ccccc3)C\2=O)cc1 |
| 310 | S1\C(=C/c2ccc(N(C)C)cc2)\C(=O)N(CCCC(O)=O)C1=S |
| 311 | S(CCOc1ccccc1F)C=1NC(=O)c2c(N=1)cccc2 |
| 312 | Brc1cc(OC)c(OC)cc1\C=C/1\Sc2c(cccc2)C\1=O |
| 313 | Clc1ccccc1COc1cc(ccc1)\C=C/1\SC(=S)N(CC(O)=O)C\1=O |
| 314 | Ic1ccccc1C(=O)Nc1ccc(cc1)-c1oc2cc(C)c(cc2n1)C |
| 315 | Ic1ccc(cc1)C(=O)Nc1cc2nc(oc2cc1)-c1ccc(Cl)cc1 |
| 316 | Ic1cc(ccc1)-c1oc2c(n1)cc(NC(=O)c1cc([N+](=O)[O-])cc([N+](=O)[O-])c1)cc2 |
| 317 | Fc1cc(ccc1)C(=O)Nc1cc(ccc1C)-c1oc2c(n1)cc(cc2C)C |
| 318 | O1c2c(C=C(c3oc(nn3)-c3ccc(OC)cc3)C1=O)cccc2OC |
| 319 | S1\C(=C/c2cc(OCC)c(OC(=O)c3cc([N+](=O)[O-])ccc3)cc2)\C(=O)NC1=O |
| 320 | S1\C(=C\c2ccccc2[N+](=O)[O-])\C(=O)N(CC)C1=S |
| 321 | Fc1cc(ccc1)C=1OC(=O)/C(/N=1)=C/c1oc(cc1)C |
| 322 | Ic1ccc(cc1)C=1OC(=O)/C(/N=1)=C/c1ccc(N(C)C)cc1 |
| 323 | O1C(=N\C(=C/c2ccc([N+](=O)[O-])cc2)\C1=O)c1cc2OCOc2cc1 |
| 324 | Brc1ccccc1\C=C\1/N=C(OC/1=O)c1cc(Cl)ccc1 |
| 325 | Fc1ccc(cc1)C=1OC(=O)/C(/N=1)=C/c1cc(OC(=O)C)c(OC)cc1 |
| 326 | O1C(=N\C(=C/c2ccc(OC(=O)C)cc2)\C1=O)c1occc1 |
| 327 | O1C(=N\C(=C/c2ccc(cc2)C)\C1=O)c1cc([N+](=O)[O-])ccc1 |
| 328 | O1C(=N\C(=C/c2ccc(OCc3ccccc3)cc2)\C1=O)c1occc1 |
| 329 | Brc1cc(cc(OCC)c1OC(=O)C)\C=C\1/N=C(OC/1=O)c1ccc(cc1)C |
| 330 | Ic1cc(ccc1)C=1OC(=O)/C(/N=1)=C/c1ccc(cc1)C(OC)=O |
| 331 | o1c2c(nc1-c1ccccc1C)cc(NC(=O)c1ccc(OCCC)cc1)cc2 |
| 332 | o1c2cc(C)c(cc2nc1-c1cc(NC(=O)c2cc(OCCC)ccc2)ccc1)C |
| 333 | Brc1c2c(ccc1)c(ccc2)-c1oc2c(n1)cc(NC(=O)c1ccccc1[N+](=O)[O-])cc2 |
| 334 | Brc1cc(-c2oc3c(n2)cc(NC(=O)c2ccc(OC)cc2)cc3)c(Cl)cc1 |
| 335 | Clc1ccc(Cl)cc1-c1oc2c(n1)cc(NC(=O)c1cc(OC)c(OC)c(OC)c1)cc2 |
| 336 | O(C)c1cc(\C=C/2\C(=NN(C\2=O)c2ccccc2)C)c([N+](=O)[O-])cc1OC |
| 337 | O(C)c1c(OC)cc(cc1OC)\C=C/1\C(=NN(C\1=O)c1ccccc1)C |
| 338 | Clc1cc(N2N=C(C)\C(=C\c3ccccc3OC)\C2=O)ccc1Cl |
| 339 | Brc1cc2OCOc2cc1\C=C\1/NC(=S)NC/1=O |
| 340 | Brc1ccc(cc1)C=1OC(=O)/C(/N=1)=C/Nc1ccccc1Cl |
| 341 | Brc1ccc(cc1)C=1OC(=O)/C(/N=1)=C/N1CCCCC1 |
| 342 | Brc1cc(ccc1)C1N(N=C(C1)c1ccc(Br)cc1)C(=O)CCC(O)=O |
| 343 | Brc1ccc(Nc2scc(n2)C2=Cc3c(OC2=O)c(OC)ccc3)cc1 |
| 344 | S1\C(=C/c2c3c([nH]c2)cccc3)\C(=O)N(c2ccc(F)cc2)C1=S |
| 345 | Brc1cc\2c(NC(=O)/C/2=C/2\SC(=S)N(NC(=O)c3ccccc3[N+](=O)[O-])C\2=O)cc1 |
| 346 | S1\C(=C/c2ccccc2OC)\C(=O)N(NC(=O)c2ccc([N+](=O)[O-])cc2)C1=S |
| 347 | S1\C(=C/c2ccccc2OC)\C(=O)N(CCC(OCCN(C)C)=O)C1=S |
| 348 | O1C(=N\C(=C/C2=Cc3c(OC2=O)c2CCCN4CCCc(c24)c3)\C1=O)c1cc(ccc1)C |
| 349 | O1C(=N\C(=C/c2cc(OC(=O)C)c(OC)cc2)\C1=O)c1cc([N+](=O)[O-])c(OC)cc1 |
| 350 | O1C(=N\C(=C/c2c3c(ccc2OC)cccc3)\C1=O)c1cc([N+](=O)[O-])c(OC)cc1 |
| 351 | Clc1c2n(nc1C(=O)N(Cc1ccccc1)c1ccccc1)C(=CC(=N2)c1sccc1)C(F)(F)F |
| 352 | Clc1ccccc1C=1OC(=O)/C(/N=1)=C\c1c(F)cccc1Cl |
| 353 | Clc1cc(C=2OC(=O)/C(/N=2)=C\c2ccc(OC)cc2OC)c(OC)cc1 |
| 354 | Clc1ccc(cc1[N+](=O)[O-])C=1OC(=O)/C(/N=1)=C\c1ccc(OC)cc1OC |
| 355 | O(C)c1ccccc1\C=N\NC(=O)C(NC(=O)c1ccccc1)C1=NNC(=O)c2c1cccc2 |
| 356 | O(C)c1cc([N+](=O)[O-])ccc1N1C(=O)C2C(C3CCC2C=C3)C1=O |
| 357 | O1c2cc(\C=N\Nc3nc(nc(n3)Nc3cccc(C)c3C)N3CCC(CC3)C)c([N+](=O)[O-])cc2OC1 |
| 358 | Ic1cc(cc(OCC)c1OC)\C=C\1/Sc2n(c3c(n2)cccc3)C/1=O |
| 359 | o1c(ccc1C1C2=C(OC3=C1C(=O)CC(C3)(C)C)CC(CC2=O)(C)C)-c1ccc([N+](=O)[O-])c(C)c1C |
| 360 | Brc1cc(C)c(Cl)cc1Nc1nc(nc(n1)N\N=C/c1cc([N+](=O)[O-])ccc1O)N1CCOCC1 |
| 361 | Brc1cc([N+](=O)[O-])cc(\C=N\Nc2nc(cc(COC)c2C#N)C)c1O |
| 362 | Brc1c(COC)c(C#N)c(OCC(=O)N\N=C\c2cc(OC)c(OCc3ccc(Cl)cc3)c(Br)c2)nc1C |
| 363 | Ic1cc(Br)cc(\C=N\NC(=O)COc2nc(C)c(Br)c(COC)c2C#N)c1O |
| 364 | O(c1ccc([N+](=O)[O-])cc1[N+](=O)[O-])c1ccc(cc1OC)\C=N\NC(=O)COc1c2ncccc2ccc1 |
| 365 | s1cccc1\C=N\Nc1nc(nc(n1)Nc1ccccc1)N1CCCC1 |
| 366 | S\1\C(=C/c2cc(OC)c(OC(=O)C34CC5CC(C3)CC(C4)C5)cc2)\C(=O)N/C/1=N\c1ccccc1 |
| 367 | S1\C(=C/c2cc(Oc3ccc([N+](=O)[O-])cc3[N+](=O)[O-])c(OC)cc2)\C(=O)N(CC=C)C1=S |
| 368 | Ic1ccccc1C(Oc1cc(ccc1)\C=C\1/SC(=S)N(C)C/1=O)=O |
| 369 | Brc1cc(cc(Br)c1O)\C=C\1/SC(=S)N(C/1=O)c1ccc(OC)cc1 |
| 370 | S1\C(=C/c2cc(OC)c(OCC=C)cc2)\C(=O)N(c2ccc([N+](=O)[O-])cc2)C1=S |
| 371 | Ic1cc(C(=O)Nc2cc3nc(oc3cc2)-c2ccc(N(C)C)cc2)c(Cl)cc1 |
| 372 | Brc1cc(-c2oc3c(n2)cc(NC(=O)c2cc(ccc2)C)cc3)c(Cl)cc1 |
| 373 | Brc1cc(C(=O)Nc2cc(ccc2)-c2sc3c(n2)cccc3)c(Cl)cc1 |
| 374 | Ic1ccc(I)cc1C(=O)Nc1ccc(cc1)-c1oc2cc(C)c(cc2n1)C |
| 375 | Clc1ccc([N+](=O)[O-])cc1C(=O)Nc1cc(-c2oc3c(n2)cc(cc3)C)c(O)cc1 |
| 376 | Clc1ccc([N+](=O)[O-])cc1C(=O)Nc1cc2nc(oc2cc1)-c1ccc(cc1)C(C)(C)C |
| 377 | S1\C(=C/c2cc([N+](=O)[O-])ccc2)\C(=O)N(c2ccccc2OC)C1=S |
| 378 | S1\C(=C/c2ccc(cc2)C)\C(=O)N(NC(=O)c2ccncc2)C1=S |
| 379 | Clc1c(cccc1Cl)\C=C\1/N=C(OC/1=O)c1ccccc1C |
| 380 | O1C(=N\C(=C/c2cc3OCOc3cc2)\C1=O)c1cc(C)c(cc1)C |
| 381 | O1C(=N\C(=C/c2ccc(N(C)C)cc2)\C1=O)c1cc(C)c(cc1)C |
| 382 | O1C(=N\C(=C/c2c3c(ccc2OC)cccc3)\C1=O)c1ccc(OCCCC)cc1 |
| 383 | Ic1ccc(N2C(=O)C3C(C4CCC3C=C4)C2=O)cc1 |
| 384 | Brc1ccc(cc1)-c1nc(sc1)NC(=O)c1ccc(S(=O)(=O)N(CCOC)CCOC)cc1 |
| 385 | O(C)c1cc(ccc1OC)C(C[N+](=O)[O-])c1c2c([nH]c1C)cccc2 |
| 386 | S\1C=2N(C(C(C(OCC)=O)=C(N=2)C)c2ccccc2)C(=O)/C/1=C\c1ccccc1OC(=O)C |
| 387 | Brc1ccc(NC(=O)CSC=2NC(=O)CC(C=2C#N)c2ccc(O)cc2)cc1 |
| 388 | S(C)C=1NC(=O)CC(C=1C#N)c1ccc(F)cc1 |
| 389 | S(C)C=1NC(=O)CC(C=1C#N)c1ccc(cc1)CC |
| 390 | s1cc(nc1C)C=1C(=O)c2c(OC=1C(F)(F)F)cc(OC)cc2 |
| 391 | s1cc(nc1C1=COc2c(cc(CC)c(OC(C(=O)NCCO)C)c2)C1=O)C |
| 392 | O1C=C(C(=O)c2c1cc(OCC(O)=O)cc2)c1cc2OCCOc2cc1 |
| 393 | FC(F)(F)C=1Oc2c(ccc(O)c2C[NH+](C)C)C(=O)C=1c1cc2OCCOc2cc1 |
| 394 | Brc1cc\2c(NC(=O)/C/2=C/2\SC(=S)N(c3ccccc3C)C\2=O)cc1 |
| 395 | S1\C(=C/c2ccccc2)\C(=O)N(CCC(OCC(C)C)=O)C1=S |
| 396 | Clc1cccc(Cl)c1\C=C\1/SC(=S)N(CCCC(O)=O)C/1=O |
| 397 | S(C(C(O)=O)c1ccccc1)c1nc(ccc1C#N)-c1ccccc1 |
| 398 | Clc1c(cccc1Cl)C1C2=C(NC(C)=C1C(OCCC)=O)CCCC2=O |
| 399 | Fc1ccccc1C1C2=C(NC(C)=C1C(OCCOc1ccccc1)=O)CC(CC2=O)(C)C |
| 400 | O1C(=N\C(=C/c2cc(OC)c(OC)c(OC)c2)\C1=O)c1ccccc1 |
| 401 | S1\C(=N\c2cc(OC)ccc2)\N(C)C(=O)CC1C(=O)Nc1ccccc1OC |
| 402 | Clc1ccc(\N=C\2/SC(CC(=O)N/2C)C(=O)Nc2ccc(OC)cc2)cc1C(F)(F)F |
| 403 | S1\C(=N\CC)\N(CC)C(=O)CC1C(=O)Nc1ccc(cc1)C(OC)=O |
| 404 | Clc1ccc(cc1)CN1/C(/SC(CC1=O)C(=O)Nc1ccc(cc1)C(O)=O)=N/c1ccccc1 |
| 405 | S1\C(=N/c2ccccc2)\N(CCc2ccccc2)C(=O)CC1C(=O)Nc1ccc(cc1)C(O)=O |
| 406 | s1c2CCCc2c2c1N=C(SCC(OCC)=O)N(C2=O)c1ccc(cc1)C |
| 407 | Fc1ccccc1\C=C\C1=Nc2c(cccc2)C(=O)N1c1ccccc1C |
| 408 | O1CC(C2(O)c3c(N(CCC)C2=O)cccc3)C(=O)CC1(C)C |
| 409 | Brc1cc(\C=C\C2=Nc3c(cccc3)C(=O)N2c2ccccc2OC)c(O)cc1 |
| 410 | Brc1c2n(nc1C(=O)NC(C)c1ccccc1)C(=CC(=N2)c1sccc1)C(F)(F)F |
| 411 | Fc1ccc(cc1)C1=Nc2n(nc(c2)C(=O)Nc2cc(O)ccc2)C(=C1)C(F)(F)F |
| 412 | Clc1cc(NC(=O)c2nn3c(N=C(C=C3C(F)(F)F)c3ccc(OC)cc3)c2)c(O)cc1 |
| 413 | s1c2c(CCC2)c(C(OCC)=O)c1NC(=O)c1nn2c(N=C(C=C2C(F)(F)F)c2ccccc2)c1 |
| 414 | FC(F)(F)C=1n2nc(cc2N=C(C=1)c1cc2OCOc2cc1)C(=O)N1CCN(CC1)C(c1ccccc1)c1ccccc1 |
| 415 | Clc1c(n(nc1C(F)(F)F)CC(=O)Nc1ccc(cc1)C#N)C |
| 416 | Clc1c(n(nc1C(F)(F)F)CC(=O)Nc1ccc(F)cc1F)C |
| 417 | Clc1c2n(nc1C(=O)NCCOC)C(=CC(=N2)c1ccc(Cl)cc1)C(F)(F)F |
| 418 | o1c2cccnc2nc1-c1ccc(NC(=O)COc2ccccc2C)cc1 |
| 419 | o1c2c(nc1-c1cc(NC(=O)c3cc4c(cc3OC)cccc4)c(OC)cc1)cccc2 |
| 420 | Clc1ccc(cc1[N+](=O)[O-])C(=O)Nc1cc(ccc1C)-c1oc2c(n1)cc(cc2C)C |
| 421 | O(C)c1ccc(cc1O)C1C2=C(NC(C)=C1C(OCc1ccc(OC)cc1)=O)CCCC2=O |
| 422 | O1c2cc(C3C4=C(NC(C)=C3C(OCCc3ccccc3)=O)CC(CC4=O)c3ccccc3)c([N+](=O)[O-])cc2OC1 |
| 423 | S1\C(=C/c2ncccc2)\C(=O)N(NC(=O)c2ccc(cc2)C)C1=S |
| 424 | Brc1ccc(cc1)C1=NN(C(=O)CCC(O)=O)C(C1)c1ccc(OC)cc1 |
| 425 | Clc1cc2c(nc(OCC)nc2-c2ccccc2)cc1 |
| 426 | Clc1cc(ccc1OCC)C(=O)Nc1cc2nc(oc2cc1)-c1ccncc1 |
| 427 | o1c2c(nc1-c1cc(NC(=O)c3cc(OCC)c(OCC)c(OCC)c3)ccc1O)cccc2 |
| 428 | Brc1cc(ccc1OCc1ccc(cc1)C(O)=O)\C=C/1\C(=O)NC(=O)NC\1=O |
| 429 | O=C/1N(N=C(C)\C\1=C\c1ccc(N(C)C)cc1)c1ccc(cc1)C(O)=O |
| 430 | S(CC(O)=O)c1nc(cc(COC)c1C#N)C |
| 431 | Brc1ccccc1C1NC(=O)NC(C)=C1C(=O)Nc1ccccc1OC |
| 432 | s1c(C(=O)Nc2ccccc2C)c(C)c(C(OCC)=O)c1NC(=O)c1cc([N+](=O)[O-])c(cc1)C |
| 433 | O=C1N(C2CCCCC2)C(=O)C2C1C1C2C2C3C(C1C=C2)C(=O)N(C1CCCCC1)C3=O |
| 434 | Clc1ccc(cc1)C1NC(=O)NC(C)=C1C(=O)Nc1ccccc1OC |
| 435 | S(CCOc1ccc(cc1)C(C)(C)C)c1nc2c(n1CCC(O)=O)cccc2 |
| 436 | Brc1cc(cc(OC)c1O)\C=C\1/C(=O)N(c2ccc(OC)cc2)C(=O)NC/1=O |
| 437 | Clc1cc(cc(Cl)c1OCC(O)=O)\C=C/1\C(=O)NC(=S)NC\1=O |
| 438 | Fc1cc(ccc1)COc1ccc(cc1OC)\C=C/1\C(=O)N(c2ccc(OC)cc2)C(=O)NC\1=O |
| 439 | Fc1ccc(N2C(=O)\C(=C\c3cc(OC)c(OCc4ccc(cc4)C(O)=O)cc3)\C(=O)NC2=O)cc1 |
| 440 | Ic1cc(cc(OC)c1OCc1ccc(Cl)cc1)\C=C/1\C(=O)N(c2ccc(OC)cc2)C(=O)NC\1=O |
| 441 | [nH]1cc(c2c1cccc2)CCNCc1cc2c3c(n(c2cc1)CC)cccc3 |
| 442 | S1(=O)(=O)N=C(OCc2ccccc2)c2c1cccc2 |
| 443 | s1ccnc1NC(=O)c1cc(NC(=O)c2cc(OC(=O)C)ccc2)ccc1 |
| 444 | Clc1c2c(sc1C(=O)N1c3c(cccc3)C(CC1(C)C)(C)c1ccc(cc1)C)cc([N+](=O)[O-])cc2 |
| 445 | Clc1cc(NC(=O)c2ccc(S(=O)(=O)N(CCC)CCC)cc2)ccc1Sc1sc2c(n1)cccc2 |
| 446 | O(CC)c1cc(ccc1OCCOc1cc(ccc1)C)\C=C/1\C(=O)NC(=O)NC\1=O |
| 447 | Clc1cc(cc(OC)c1OCCOc1cc(ccc1)C)\C=C\1/C(=O)N(c2cc(ccc2)C)C(=O)NC/1=O |
| 448 | Brc1cc(cc(OCC)c1OCCOc1ccc(cc1)C)\C=C/1\C(=O)N(C)C(=O)N(C)C\1=O |
| 449 | BrCCOc1ccc(cc1)\C=C/1\C(=O)NC(=O)NC\1=O |
| 450 | s1c2CCCc2c2c1N=C(SCCCC)N(C1CCCCC1)C2=O |
| 451 | s1c2CCCCc2c2c1N=C(SCC(C)=C)N(C2=O)c1ccccc1 |
| 452 | o1c2c(nc1-c1cccnc1)cc(NC(=O)c1cc(OCC)cc(OCC)c1)cc2 |
| 453 | Clc1cc(cc(OC)c1O)C1C2=C(NC(C)=C1C(OCC1OCCC1)=O)CC(CC2=O)(C)C |
| 454 | O(C)c1cc(ccc1O)C1C2=C(NC(C)=C1C(OCc1ccccc1)=O)CC(CC2=O)c1ccccc1 |
| 455 | Ic1ccccc1C1C2=C(NC(C)=C1C(OCc1ccccc1)=O)CCCC2=O |
| 456 | O(CCOC(=O)Nc1ccccc1)c1nc(nc(n1)N(C)C)N(C)C |
| 457 | S(=O)(=O)(N)c1ccc(N2C(=O)C3C(CC=C(C3)C)C2=O)cc1 |
| 458 | s1c2cc(S(=O)(=O)C)ccc2nc1NC(=O)c1cc(ccc1)C |
| 459 | O1C=C(C2C3=C(NC(C)=C2C(OCc2ccc(OC)cc2)=O)CC(CC3=O)(C)C)C(=O)c2c1cccc2 |
| 460 | O=C1N(NC(=O)c2ccc(cc2)C(C)(C)C)C(=Nc2c1cccc2)c1cc(C)c(cc1)C |
| 461 | O=C1N(NC(=O)C2CCC(CC2)CCCCCC)C(=Nc2c1cccc2)c1cc([N+](=O)[O-])ccc1 |
| 462 | O(CC(=O)NN1C(=Nc2c(cccc2)C1=O)C)c1ccccc1 |
| 463 | s1c(C(=O)Nc2ccccc2F)c(C)c(C(OCC)=O)c1NC(=O)Cc1ccc(OC)cc1 |
| 464 | S(=O)(=O)(N1CCOCC1)c1cc(ccc1C)C(=O)Nc1ccccc1 |
| 465 | S(=O)(=O)(N1CCCC1)c1cc(ccc1)C(=O)Nc1ccccc1O |
| 466 | Clc1ccc(cc1S(=O)(=O)N1CCCCC1)C(=O)N1CCN(CC1)c1ccccc1 |
| 467 | FC(F)(F)c1ccc(cc1)C1NC(=O)N(C)C(C)=C1C(OCCc1ccccc1)=O |
| 468 | Fc1ccccc1-c1oc2c(n1)cc(NC(=O)c1cc(OC)c(OC)cc1)cc2 |
| 469 | o1c2cc(ccc2nc1-c1ccc(NC(=O)c2cc(OC)cc(OC)c2)cc1)C |
| 470 | Brc1ccc(Oc2ccc(NC(=O)c3cc(nc4c3cccc4)-c3ccccc3)cc2)cc1 |
| 471 | Oc1cc(N2C(=O)C3C(CCC(C3)C)C2=O)ccc1 |
| 472 | Clc1ccc(cc1)CC(=O)N1c2c(NC3=C(C1c1ccccc1F)C(=O)CC(C3)(C)C)cccc2 |
| 473 | s1cccc1C1N(c2c(NC3=C1C(=O)CC(C3)(C)C)cccc2)C(=O)Cc1ccccc1 |
| 474 | FC(F)(F)C(=O)N1c2c(NC3=C(C1c1oc(cc1)-c1ccccc1[N+](=O)[O-])C(=O)CC(C3)(C)C)cccc2 |
| 475 | Fc1cc(ccc1)C1N(c2c(NC3=C1C(=O)CC(C3)(C)C)cccc2)C(=O)Cc1ccccc1 |
| 476 | O=C1C2=C(Nc3c(N(C(=O)C(C)C)C2c2ccc([N+](=O)[O-])cc2)cccc3)CC(C1)(C)C |
| 477 | Clc1ccccc1C(Oc1cc2OC=C(Oc3ccc(OC)cc3)C(=O)c2cc1)=O |
| 478 | O(C)c1ccccc1C1Nc2c(NC3=C1C(=O)CC(C3)c1ccc(OC)cc1)cccc2 |
| 479 | Clc1cc(Cl)ccc1C(=O)NN1C(=Nc2c(cccc2)C1=O)C |
| 480 | Clc1ccc(cc1)C(=O)NN1C(=Nc2c(cccc2)C1=O)c1cc(OC)c(OC)cc1 |
| 481 | O=C1N(C(CCCCN2C(=O)C3C(C4CC3C=C4)C2=O)C(OC)=O)C(=O)C2C1C1CC2C=C1 |
| 482 | Brc1ccccc1C=1OC(=O)/C(/N=1)=C/c1oc(Br)cc1 |
| 483 | Clc1cc(F)c(F)cc1C=1OC(=O)/C(/N=1)=C/c1cc2OCOc2cc1 |
| 484 | Brc1ccccc1\C=C\1/N=C(OC/1=O)c1ccc(F)cc1 |
| 485 | Clc1cc(Cl)ccc1\C=C\1/N=C(OC/1=O)c1cc2OCOc2cc1 |
| 486 | Clc1ccccc1C=1OC(=O)/C(/N=1)=C/c1cc2c(cc1)cccc2 |
| 487 | O1C(=N\C(=C/c2ccccc2C)\C1=O)c1ccc(cc1)C |
| 488 | Clc1ccc([N+](=O)[O-])cc1C=1OC(=O)/C(/N=1)=C/c1ccc(OCc2ccccc2)cc1 |
| 489 | O1C(=N\C(=C/c2oc(cc2)-c2cc([N+](=O)[O-])ccc2)\C1=O)c1cc([N+](=O)[O-])c(cc1)C |
| 490 | Fc1cc(ccc1)C=1OC(=O)/C(/N=1)=C/c1ccc(cc1)C(OC)=O |
| 491 | s1c2c(nc1-c1cc(NC(=O)c3cc(OCCC)ccc3)ccc1)cccc2 |
| 492 | Brc1ccc([N+](=O)[O-])cc1C(=O)Nc1cc(ccc1C)-c1oc2c(n1)cccc2 |
| 493 | o1c2c(nc1-c1cc(NC(=O)c3ccccc3[N+](=O)[O-])c(cc1)C)cc(cc2)C |
| 494 | Clc1ccc(cc1[N+](=O)[O-])C(=O)Nc1cc(ccc1)-c1oc2c(n1)cc(Cl)cc2 |
| 495 | o1c2c(nc1-c1cc(NC(=O)c3cc(OC)c(OC)c(OC)c3)ccc1O)cccc2 |
| 496 | Clc1ccc(N2N=C(C)\C(=C\c3cc(OC)c(OCc4ccccc4)cc3)\C2=O)cc1C(O)=O |
| 497 | O(Cc1ccccc1)c1ccc(cc1OC)\C=C/1\C(=NN(C\1=O)c1ccccc1)C |
| 498 | Clc1cc(N2N=C(C)\C(=C/c3cc(OC)c(OC)cc3)\C2=O)ccc1Cl |
| 499 | S=C1NC(=O)/C(/N1)=C\c1ccccc1OCC=C |
| 500 | s1cccc1\C=C\1/N=C(OC/1=O)\C=C\c1ccccc1OC |
| 501 | Clc1cc(Cl)ccc1Nc1nc(c2c(n1)cccc2)-c1ccccc1 |
| 502 | S1\C(=C/C=C/2\N(C=CC=C\2)CC)\C(=O)N(CC)C1=S |
| 503 | Brc1ccc(Nc2scc(n2)C2=Cc3cc(Br)ccc3OC2=O)cc1 |
| 504 | O1c2c(OCC1C(=O)N\N=C\c1c3c([nH]c1)cccc3)cccc2 |
| 505 | S1\C(=C/c2ccc(cc2)C(OC)=O)\C(=O)N(c2ccc(N(CC)CC)cc2)C1=S |
| 506 | S1\C(=C/c2ccccc2)\C(=O)N(CCCC(=O)Nc2ccc(cc2)C(OCCN(CC)CC)=O)C1=S |
| 507 | S1\C(=C/c2cc(OC)ccc2)\C(=O)N(CCC(=O)NNC(=O)c2ccccc2OC)C1=S |
| 508 | Clc1ccc(cc1[N+](=O)[O-])C=1OC(=O)/C(/N=1)=C/C1=Cc2c(OC1=O)c1CCCN3CCCc(c13)c2 |
| 509 | Clc1ccc(cc1)C=1OC(=O)/C(/N=1)=C/Nc1sc2cc(OC)ccc2n1 |
| 510 | Clc1cc(NC(=O)c2sc(NC(=O)c3ccccc3F)c(C(OCC)=O)c2C)ccc1 |
| 511 | Clc1c2c(sc1C(=O)Nc1ccc(OC)cc1OC)cc([N+](=O)[O-])cc2 |
| 512 | O1C(=O)/C(/N=C1\C=C\c1cc([N+](=O)[O-])ccc1)=C\Nc1cc([N+](=O)[O-])ccc1C |
| 513 | O1C(=N\C(=C/c2cc3OCOc3cc2)\C1=O)c1cc(OC)c(OC)c(OC)c1 |
| 514 | Clc1cc(C=2OC(=O)/C(/N=2)=C\c2cc(OC)c(OC)cc2OC)c(OC)cc1 |
| 515 | Brc1c2c(sc1\C=N\NC(=O)C(NC(=O)c1ccccc1)C1=NNC(=O)c3c1cccc3)cccc2 |
| 516 | S1\C(=C\c2ccc(cc2)C(OC)=O)\C(=O)N(CNc2ccc(cc2)C(OCCCC)=O)C1=O |
| 517 | S\1c2n(c3cc(C)c(cc3n2)C)C(=O)/C/1=C\c1cc(OC)c(OCc2ccccc2)cc1 |
| 518 | o1nc2nc(Nc3ccc(cc3)C(OCC)=O)c(nc2n1)N\N=C\c1ccccc1[N+](=O)[O-] |
| 519 | Brc1cc(Nc2nc3nonc3nc2Nc2ccc(O)cc2)ccc1 |
| 520 | Ic1oc(cc1)\C=N\Nc1nc(cc(COC)c1C#N)C |
| 521 | o1c(ccc1\C=N\Nc1nc(cc(COC)c1C#N)C)-c1cc([N+](=O)[O-])ccc1C |
| 522 | Brc1c(COC)c(C#N)c(OCC(=O)N\N=C\c2c3c(ccc2O)cccc3)nc1C |
| 523 | Ic1ccc(cc1)-c1oc(cc1)\C=N\NC(=O)COc1nc(C)c(Br)c(COC)c1C#N |
| 524 | S1\C(=C\c2cc(n(c2C)-c2ccccc2)C)\C(=O)N(C)C1=O |
| 525 | Clc1cc(N2C(=O)/C(/SC2=O)=C/c2sccc2)ccc1Cl |
| 526 | Clc1ccccc1COc1ccccc1\C=C\1/NC(=S)NC/1=O |
| 527 | Clc1cc(NCN2C(=O)/C(/SC2=O)=C\c2ccc(F)cc2)c(OC)cc1 |
| 528 | Clc1cc(N2C(=O)/C(/SC2=S)=C/c2cc(OC)c(OCC=C)cc2)ccc1 |
| 529 | S1\C(=C/c2oc(cc2)-c2ccc([N+](=O)[O-])cc2C)\C(=O)N(Cc2ccccc2)C1=S |
| 530 | Clc1c(cccc1Cl)C(=O)Nc1cc2nc(oc2cc1)-c1ccc(F)cc1 |
| 531 | o1c2c(nc1-c1ccc(N(C)C)cc1)cc(NC(=O)c1ccc([N+](=O)[O-])cc1[N+](=O)[O-])cc2 |
| 532 | o1c2c(nc1-c1cc(ccc1)C)cc(NC(=O)c1cc(OC)c(OC)c(OC)c1)cc2 |
| 533 | Brc1cc(C(=O)Nc2cccc(-c3oc4c(n3)cc(cc4C)C)c2C)c(Cl)cc1 |
| 534 | Ic1ccc(I)cc1C(=O)Nc1cc(ccc1)-c1oc2c(n1)cccc2 |
| 535 | Ic1ccc(I)cc1C(=O)Nc1cc2nc(oc2cc1)-c1ccc(cc1)C(C)(C)C |
| 536 | s1c2c(nc1-c1cc(NC(=O)c3ccc(OCC)cc3)c(cc1)C)cccc2 |
| 537 | Clc1ccc(cc1[N+](=O)[O-])C(=O)Nc1scc(n1)CC(OCC)=O |
| 538 | S1\C(=C/2\SC(=S)N(CCC(=O)Nc3sccn3)C\2=O)\C(=O)N(CCC(=O)Nc2sccn2)C1=S |
| 539 | O1C(=N/C(=C\C=C\c2ccccc2OC)/C1=O)c1ccccc1C |
| 540 | O1C(=N\C(=C/c2ccccc2OCC)\C1=O)c1cc(C)c(cc1)C |
| 541 | O1C(=N\C(=C\C2=COc3c(cccc3)C2=O)\C1=O)c1cc(C)c(cc1)C |
| 542 | O1C(=N\C(=C/c2ccc(OC)cc2OC)\C1=O)c1ccc(OCCCC)cc1 |
| 543 | o1cccc1C1=Nc2c(cccc2)C(=O)N1CCc1ccccc1 |
| 544 | Clc1c2nc(sc2ccc1)NC(=O)c1ccccc1Oc1ccccc1 |
| 545 | Brc1cc2sc(nc2cc1)NC(=O)c1ccc(S(=O)(=O)N(CCOC)CCOC)cc1 |
| 546 | Brc1cc(ccc1)\C=C/1\SC=2N(C(C(C(OCC)=O)=C(N=2)C)c2ccccc2)C\1=O |
| 547 | s1cc(nc1/C(=C\c1cc(OCC)c(O)cc1)/C#N)C1=Cc2c3c(ccc2OC1=O)cccc3 |
| 548 | S(Cc1ccccc1)C=1NC(=O)CC(C=1C#N)c1cc(OC)ccc1 |
| 549 | s1cc(nc1C1=Cc2c3c(ccc2OC1=O)cccc3)-c1ccc(OC)cc1 |
| 550 | s1cc(nc1C)C=1C(=O)c2c(OC=1C(F)(F)F)c(C)c(cc2)C |
| 551 | o1c2c(cc1C1=COc3c(ccc(O)c3)C1=O)cccc2 |
| 552 | FC(F)(F)C=1Oc2c(cc(CC)c(OC(C)C)c2)C(=O)C=1c1cc2OCOc2cc1 |
| 553 | O1c2c(cc(CCC)c(OC(=O)C)c2)C(=O)C(c2cc3OCOc3cc2)=C1C |
| 554 | S1\C(=C/2\c3c(NC\2=O)cccc3)\C(=O)N(c2ccccc2OC)C1=S |
| 555 | Clc1ccc(cc1)\C=C\1/SC(=S)N(CCCCCC(OCC)=O)C/1=O |
| 556 | Clc1cc(Cl)ccc1\C=C\1/SC(=S)N(C/1=O)c1ccc(cc1)C |
| 557 | S(CC(OCC)=O)c1nncc2c1cccc2 |
| 558 | O=C1N(C(=O)C2C1C(C=C(C2)C)C)c1ccc(cc1)C(O)=O |
| 559 | O(c1cc(ccc1)C1C2=C(NC(C)=C1C(OCCc1ccccc1)=O)CC(CC2=O)(C)C)c1ccccc1 |
| 560 | O(CCOC(=O)C=1C(C2=C(NC=1C)CC(CC2=O)(C)C)c1cccnc1)c1ccccc1 |
| 561 | S(CC#N)C1=Nc2c(ccc(c2)C(=O)NCCC=2CCCCC=2)C(=O)N1CCCCC |
| 562 | S(Cc1ncccc1)C1=Nc2c(ccc(c2)C(=O)NCc2ccccc2)C(=O)N1CCCCC |
| 563 | s1c2N=C(SCc3cc([N+](=O)[O-])ccc3)N(Cc3ccccc3)C(=O)c2c(C)c1C(=O)Nc1ccccc1C(F)(F)F |
| 564 | S1C2=C(N=C(SCc3cc([N+](=O)[O-])ccc3)N(C2=O)c2ccccc2)N(c2ccccc2OC)C1=S |
| 565 | S1C2=C(N=C(SCc3ccncc3)N(C2=O)c2ccccc2)N(c2cc(ccc2)C(F)(F)F)C1=S |
| 566 | Clc1cc(N2C(=O)C=3SC(=S)N(C=3N=C2SCC(OCC)=O)Cc2ccccc2)ccc1 |
| 567 | S1C2=C(N=C(SCc3ccncc3)N(c3c(cccc3C)C)C2=O)N(CCc2ccccc2)C1=S |
| 568 | S=C1Nc2c(ccc(c2)C(=O)N(CC)CC)C(=O)N1Cc1ccccc1 |
| 569 | Clc1cc(NC(=O)c2cc3NC(=S)N(CCc4ccccc4)C(=O)c3cc2)ccc1 |
| 570 | S=C1Nc2c(ccc(c2)C(=O)N2CCN(CC2)c2ccccc2)C(=O)N1c1ccccc1F |
| 571 | S=C1Nc2c(ccc(c2)C(=O)N2CCOCC2)C(=O)N1c1cc(OC)c(OC)cc1 |
| 572 | Brc1cc(ccc1)CN1C(=O)c2c(NC1=S)cc(cc2)C(=O)N1CCN(CC1)C(OCC)=O |
| 573 | S=C1Nc2c(ccc(c2)C(=O)Nc2ccccc2OC)C(=O)N1c1ccccc1 |
| 574 | Clc1cc(NC(=O)c2cc3NC(=S)N(C(=O)c3cc2)c2ccccc2)ccc1OC |
| 575 | Clc1ccc(cc1)CCNC(=O)c1cc2NC(=S)N(C3CCCCC3)C(=O)c2cc1 |
| 576 | S=C1Nc2c(ccc(c2)C(=O)NC2CCCC(C)C2C)C(=O)N1CC=C |
| 577 | S=C1Nc2c(ccc(c2)C(=O)NCC2OCCC2)C(=O)N1c1ccc(OC)cc1OC |
| 578 | S=C1Nc2c(ccc(c2)C(=O)NCCCN2CCCC2=O)C(=O)N1c1ccc(OC)cc1OC |
| 579 | S=C1Nc2c(ccc(c2)C(=O)NCCc2cc(OC)c(OC)cc2)C(=O)N1Cc1occc1 |
| 580 | S=C1Nc2c(ccc(c2)C(=O)NCCCN2CCOCC2)C(=O)N1CC1OCCC1 |
| 581 | Clc1cc(NC(=O)c2cc3NC(=S)N(C(=O)c3cc2)c2cc(OC)c(OC)cc2)ccc1F |
| 582 | Brc1cc(ccc1)CN1C(=O)c2c(NC1=S)cc(cc2)C(=O)NCc1ccc(Cl)cc1 |
| 583 | S=C1Nc2c(ccc(c2)C(=O)NCCc2cc(OC)c(OC)cc2)C(=O)N1Cc1ccc(OC)cc1 |
| 584 | s1ccnc1NC(=O)CSc1nnc(n1-c1ccccc1OC)CNC(=O)c1cc(OC)c(OC)c(OC)c1 |
| 585 | s1ccnc1NC(=O)CSc1nnc(n1-c1ccccc1OC)CNC(=O)c1ccc(S(=O)(=O)N2CCCC2)cc1 |
| 586 | S(CC(=O)N1CCc2c1cccc2)c1nnc(n1-c1cc(OC)ccc1OC)CNC(=O)c1ccccc1OC |
| 587 | S(CC(=O)N1CCCc2c1cccc2)c1nnc(n1-c1cc(OC)ccc1OC)CNC(=O)c1ccc(S(=O)(=O)N(C)C)cc1 |
| 588 | s1cccc1C(=O)NCc1nnc(SCC(=O)Nc2ccc(OC)cc2)n1-c1cc(OC)ccc1OC |
| 589 | s1ccnc1NC(=O)CSc1nnc(n1-c1cc(ccc1)C)CNC(=O)c1cc(OC)c(OC)cc1 |
| 590 | S(CC(=O)N1CCc2c1cccc2)c1nnc(n1-c1cc(ccc1)C)CNC(=O)c1ccc(S(=O)(=O)N2CCCCC2)cc1 |
| 591 | S(CC(=O)Nc1ccc(F)cc1)c1nnc(n1-c1ccc(F)cc1)CNC(=O)c1occc1 |
| 592 | s1ccnc1NC(=O)CSc1nnc(n1-c1ccc(OCC)cc1)CNC(=O)c1occc1 |
| 593 | S1C2=C(N=C(SCC(=O)Nc3ccccc3C(F)(F)F)N(c3ccccc3OC)C2=O)CC1 |
| 594 | S1C(C(=O)NCCc2ccccc2)=C(N)N(C2CCCCC2)C1=S |
| 595 | Clc1cc(N2C(=O)C=3SC(=S)N(C=3NC2=S)c2c(cccc2C)C)ccc1 |
| 596 | s1cccc1C1=NN(C(=O)CSc2nnc(n2-c2ccccc2)CNC(=O)c2ccccc2)C(C1)c1cccc(OC)c1OC |
| 597 | Brc1ccc(cc1)C1=NN(C(=O)CSc2nnc(n2C)CNC(=O)c2ccccc2)C(C1)c1ccc(OC)cc1 |
| 598 | s1cccc1C1=NN(C(=O)CSc2nnc(n2-c2ccccc2OC)CNC(=O)c2ccccc2F)C(C1)c1ccc(OC)cc1 |
| 599 | s1cccc1C1=NN(C(=O)CSc2nnc(n2-c2ccc(OCC)cc2)CNC(=O)c2cc(F)ccc2)C(C1)c1cccc(OC)c1OC |
| 600 | s1cccc1C1=NN(C(=O)CSc2nnc(n2-c2ccc(OCC)cc2)CNC(=O)c2ccc([N+](=O)[O-])cc2)C(C1)c1ccc(OC)cc1 |
| 601 | s1cccc1C1=NN(C(=O)CSc2nnc(n2-c2ccccc2OC)CNC(=O)c2ccccc2OC)C(C1)c1ccc(F)cc1 |
| 602 | s1cccc1C1=NN(C(=O)CSc2nnc(n2-c2cccc(C)c2C)CNC(=O)c2cc(OC)ccc2)C(C1)c1ccc(F)cc1 |
| 603 | s1cccc1C1=NN(C(=O)CSc2nnc(n2-c2cc(ccc2C)C)CNC(=O)c2ccc(OC)cc2)C(C1)c1ccc(F)cc1 |
| 604 | s1cccc1C1=NN(C(=O)CSc2nnc(n2C)CNC(=O)c2cccc([N+](=O)[O-])c2C)C(C1)c1cccc(OC)c1OC |
| 605 | s1cccc1C1=NN(C(=O)CSc2nnc(n2-c2ccccc2OC)CNC(=O)c2cc(OC)c(OC)cc2)C(C1)c1ccc(OC)cc1 |
| 606 | s1cccc1C1=NN(C(=O)CSc2nnc(n2-c2ccccc2)CNC(=O)Cc2ccccc2)C(C1)c1cccc(OC)c1OC |
| 607 | s1cccc1C1=NN(C(=O)CSc2nnc(n2-c2ccccc2OC)CNC(=O)COc2ccccc2)C(C1)c1ccc(cc1)C |
| 608 | Brc1ccc(cc1)C1=NN(C(=O)CSc2nnc(n2-c2ccccc2)CNC(=O)c2occc2)C(C1)c1ccc(cc1)C |
| 609 | S(CC(=O)N1N=C(CC1c1cccc(OC)c1OC)c1ccc(OC)cc1)c1nnc(n1-c1ccc(F)cc1)CNC(=O)c1occc1 |
| 610 | S(CC(=O)N1N=C(CC1c1ccc(OC)cc1)c1ccc(OC)cc1)c1nnc(n1-c1cc(ccc1)C)CNC(=O)c1occc1 |
| 611 | s1cccc1C(=O)NCc1nnc(SCC(=O)N2N=C(CC2c2ccc(F)cc2)c2ccc(OC)cc2)n1-c1ccccc1OC |
| 612 | Brc1ccc(cc1)C1=NN(C(=O)CSc2nnc(n2Cc2ccccc2)CNC(=O)c2occc2)C(C1)c1ccc(cc1)C |
| 613 | s1cccc1C1=NN(C(=O)CSc2nnc(n2Cc2ccccc2)CNC(=O)c2sccc2)C(C1)c1ccc(cc1)C |
| 614 | S(CC(=O)N1N=C(CC1c1ccc(cc1)C)c1ccc(OC)cc1)c1nnc(n1CCc1ccccc1)CNC(=O)c1occc1 |
| 615 | s1cccc1C1=NN(C(=O)CSc2nnc(n2CCc2ccccc2)CNC(=O)c2occc2)C(C1)c1ccc(F)cc1 |
| 616 | Fc1ccc(cc1)CNc1ccccc1C1=Nc2c(NC1=O)cccc2 |
| 617 | Clc1cc(\N=C\2/Oc3c(C=C/2C(=O)NC(=O)C)c2c(cc3)cccc2)ccc1F |
| 618 | Brc1cc(F)c(NC(=O)C=2C(=O)Nc3c(cccc3)C=2O)cc1 |
| 619 | Clc1c2c(sc1C(=O)N\N=C\c1cccc(OC)c1O)cccc2 |
| 620 | s1c(C)c(C)c(C(OCC)=O)c1NC(=O)c1nn2c(N=C(C=C2C(F)(F)F)c2ccc(OC)cc2)c1 |
| 621 | Clc1c2n(nc1C(=O)N1CCN(CC1)C(=O)c1ccccc1)C(=CC(=N2)c1ccccc1)C(F)(F)F |
| 622 | Brc1c2n(nc1C(=O)Nc1ccc(cc1)C#N)C(=CC(=N2)c1sccc1)C(F)(F)F |
| 623 | FC(F)(F)C=1n2ncc(c2N=C(C=1)c1cc(OC)c(OC)cc1)C(=O)NC(C)c1ccccc1 |
| 624 | s1cccc1C1=Nc2n(ncc2C(=O)Nc2c3c(ccc2)cccc3)C(=C1)C(F)(F)F |
| 625 | FC(F)(F)C=1n2nc(cc2N=C(C=1)c1ccccc1)C(=O)NCC#N |
| 626 | Brc1c2n(nc1C(=O)Nc1sc3c(CCCC3)c1C(OC)=O)C(=CC(=N2)c1sccc1)C(F)(F)F |
| 627 | Brc1c(n(nc1[N+](=O)[O-])CC(=O)Nc1sc2c(CCCC2)c1C(=O)N)C |
| 628 | s1c2c(CCCCC2)c(C#N)c1NC(=O)Cn1nc([N+](=O)[O-])nc1 |
| 629 | FC(F)(F)C=1n2ncc(c2N=C(C=1)c1ccccc1)C(=O)NC12CC3CC(C1)CC(C2)C3 |
| 630 | Brc1ccc(cc1)C1=Nc2n(nc(c2)C(=O)Nc2cc(OC)c(OC)c(OC)c2)C(=C1)C(F)(F)F |
| 631 | Brc1ccc(cc1)C1=Nc2n(ncc2C(=O)Nc2sc3c(CCCC3)c2C(OC)=O)C(=C1)C(F)(F)F |
| 632 | Brc1ccc(cc1)C1=Nc2n(nc(C(=O)N3CCc4c(C3)cccc4)c2Cl)C(=C1)C(F)(F)F |
| 633 | FC(F)(F)C=1n2nc(cc2N=C(C=1)c1ccc([N+](=O)[O-])cc1)C(=O)N1CCCCC1 |
| 634 | Clc1cc(ccc1Cl)C1=Nc2n(nc(c2)C(=O)NC2CCCCC2)C(=C1)C(F)(F)F |
| 635 | Clc1cccc(F)c1\C=C\1/N=C(OC/1=O)c1cc(OC)c(OC)c(OC)c1 |
| 636 | Brc1oc(cc1)C=1OC(=O)/C(/N=1)=C/c1ccccc1Cl |
| 637 | O1C(=N\C(=C\c2cc(OC)c(OC)c(OC)c2)\C1=O)c1ccc(cc1)C(C)(C)C |
| 638 | O1C(=N\C(=C/c2cc(OC)ccc2)\C1=O)c1cc(OC)ccc1 |
| 639 | Fc1ccc(cc1)\C=C\1/N=C(OC/1=O)c1ccc(cc1)-c1ccc(cc1)CCCCCCCC |
| 640 | O1C(=N\C(=C\c2c3c(ccc2OC)cccc3)\C1=O)c1ccc([N+](=O)[O-])cc1 |
| 641 | s1c(nnc1Nc1ccc(F)cc1)C1=Cc2c(OC1=O)cccc2 |
| 642 | O(C(=O)c1cc2C3C(CC=C3)C(Nc2cc1)c1ccc([N+](=O)[O-])cc1)CC |
| 643 | O=C1NC(C(C(OCCC)=O)=C(N1C)C)c1ccc([N+](=O)[O-])cc1 |
| 644 | Clc1ccc(cc1)C1C2=C(NC(C)=C1C#N)CC(CC2=O)(C)C |
| 645 | O1C2=C(C(C(C(OC)=O)=C1N)c1ccc(OC)cc1)C(=O)CCC2 |
| 646 | Cl\C(=C\Cn1c2c(nc1SCC=C)N(C)C(=O)NC2=O)\C |
| 647 | Brc1ccc(cc1)C(=O)CSc1nc2CCCCCc2cc1C#N |
| 648 | Clc1cc(ccc1Cl)C1NC(=O)N(C)C(C)=C1C(OCc1ccccc1)=O |
| 649 | S=C1NC(=O)\C(=C\c2cc(O)c(OC)cc2)\C(=O)N1 |
| 650 | S1\C(=C/c2cc(OC)c(OCC)cc2)\C(=O)N(c2ccc(OC)cc2)C1=S |
| 651 | Brc1c2n(nc1C(=O)Nc1ccc([N+](=O)[O-])cc1Cl)C(=CC(=N2)c1ccc(OC)cc1)C(F)(F)F |
| 652 | Brc1cc(F)cc(F)c1NC(=O)Cn1nc(cc1C)C(F)(F)F |
| 653 | Clc1cc(NC(=O)c2nn3c(N=C(C=C3C)c3ccccc3)c2)c(OC)cc1 |
| 654 | Clc1cc(NC(=O)Cn2nc([N+](=O)[O-])nc2)ccc1F |
| 655 | O1CCCC1CNC(=O)c1nn2c(N=C(C=C2c2ccccc2)c2ccccc2)c1 |
| 656 | O1C(=N\C(=C/c2occc2)\C1=O)c1cc(OC)c(OC)cc1 |
| 657 | Oc1cc(N(CC)CC)ccc1\C=N\NC(=O)Cc1c2c([nH]c1)cccc2 |
| 658 | S\1C=2N(C(C(C(OCC)=O)=C(N=2)C)c2ccc(OC(=O)C)cc2)C(=O)/C/1=C/c1cn(nc1-c1ccccc1)-c1ccccc1 |
| 659 | O=C1C2=C(NC(C)=C(C#N)C2c2ccc(N(C)C)cc2)CCC1 |
| 660 | Fc1ccccc1NC(=O)C1=Cc2c(OC1=O)c(ccc2)CC=C |
| 661 | Brc1cc2C=C(c3nc(sc3)Nc3ccc(Cl)cc3Cl)C(Oc2cc1)=O |
| 662 | O\1c2c(C=C(C(=O)N)/C/1=N/c1cc(ccc1)C)cccc2OCC |
| 663 | S(C)c1nc2OC(Nc3c(-c2nn1)cccc3)c1ccc(cc1)C(C)C |
| 664 | O1C2=C(C(C(C#N)=C1N)c1c3c(cc4c1cccc4)cccc3)C(=O)CC(C2)(C)C |
| 665 | Clc1ccc(cc1[N+](=O)[O-])-c1oc(cc1)\C=C/1\SC(=S)N(CC=C)C\1=O |
| 666 | S1\C(=C\c2cc3c(cc2)cccc3)\C(=O)N(CC)C1=S |
| 667 | S1\C(=C\c2cc(ccc2)C(F)(F)F)\C(=O)N(CC)C1=S |
| 668 | Brc1ccc(S(=O)(=O)CCc2sc(nn2)NC(=O)c2ccccc2)cc1 |
| 669 | Clc1ccc(S(=O)(=O)CCc2sc(nn2)NC(=O)C2CCCCC2)cc1 |
| 670 | Clc1ccc(cc1N\N=C\1/C(=NN(C/1=O)c1scc(n1)-c1ccc(OC)cc1)C)C(F)(F)F |
| 671 | Clc1ccc(cc1)-c1nc(sc1)N1N=C(C)\C(=N/Nc2ccccc2[N+](=O)[O-])\C1=O |
| 672 | Brc1ccc(cc1)C1N2CC3C(C=CC3)c3cc(cc(C4C1CC=C4)c23)C(OCC)=O |
| 673 | O(C(=O)C)c1ccc(cc1OC)C1C2=C(NC(C)=C1C(OCC)=O)CCCC2=O |
| 674 | O1C(=N\C(=C/c2ccccc2)\C1=O)c1cc([N+](=O)[O-])c(cc1)C |
| 675 | O1C(=N\C(=C/c2cc([N+](=O)[O-])ccc2)\C1=O)c1cc(OC)c(OC)c(OC)c1 |
| 676 | Clc1cc(ccc1Cl)C(=O)N\N=C\c1cc(CSc2oc3c(n2)cccc3)c(OC)cc1 |
| 677 | S\1\C(=C/c2ccccc2)\C(=O)N(/C/1=N/c1ccccc1OC)c1ccccc1OC |
| 678 | Brc1cc\2c(NC(=O)/C/2=C/2\S\C(=N/c3ccc(cc3)C)\N(C\2=O)c2ccc(cc2)C)cc1 |
| 679 | O1c2c(C=C(C(=O)Nc3ccc([N+](=O)[O-])cc3OC)C1=O)cccc2 |
| 680 | S1\C(=C\c2cc(OC)c(OC)cc2)\C(=O)N(CC(=O)Nc2noc(c2)C)C1=S |
| 681 | S\1\C(=C/c2ccc(N(C)C)cc2)\C(=O)N(/C/1=N/Nc1ccccc1)c1ccccc1 |
| 682 | Ic1ccccc1N\C=C\1/N=C(OC/1=O)c1ccccc1F |
| 683 | Clc1cccc(F)c1\C=C\1/N=C(OC/1=O)c1cc([N+](=O)[O-])c(cc1)C |
| 684 | Brc1ccccc1C=1OC(=O)/C(/N=1)=C/Nc1cc(Cl)ccc1 |
| 685 | s1c2c(CCCC2)c(C(OCC)=O)c1NC(=O)C1N(S(=O)(=O)c2c3nsnc3ccc2)CCC1 |
| 686 | O=[N+]([O-])c1ccc(Nc2nc(nc(n2)N\N=C\c2c3c(cc4c2cccc4)cccc3)N2CCCC2)cc1 |
| 687 | S1\C(=C/c2ccsc2)\C(=O)N(CC(O)=O)C1=S |
| 688 | Clc1ccccc1\N=C\1/S\C(=C/c2cc(OC)ccc2OC)\C(=O)N/1 |
| 689 | S(CCC(O)=O)c1nc2c(n1CCC)cccc2 |
| 690 | Brc1ccccc1C(OCCCNC1=NS(=O)(=O)c2c1cccc2)=O |
| 691 | Brc1cc2OCOc2cc1C1NC(=O)N(C)C(C)=C1C(OC1CCCC1)=O |
| 692 | s1ccnc1NC(=O)c1ccc(OCCCC)cc1 |
| 693 | o1c2c(nc1-c1cc(NC(=O)c3ccccc3)ccc1O)cc(cc2)C |
| 694 | Clc1ccc(NC(=O)c2cc([N+](=O)[O-])c(OCC)cc2)cc1-c1oc2c(n1)cccc2 |
| 695 | S1\C(=C\c2ccc(SC)cc2)\C(=O)N(CNc2ccc(cc2)C(OC)=O)C1=O |
| 696 | S1\C(=C\C(=C\c2ccccc2)\C)\C(=O)N(c2cc(F)ccc2)C1=S |
| 697 | S1\C(=C/c2cc(Oc3ccc([N+](=O)[O-])cc3[N+](=O)[O-])ccc2)\C(=O)N(C2CCCCC2)C1=S |
| 698 | Clc1ccc(N2C(=O)/C(/SC2=O)=C/c2ccccc2OC)cc1 |
| 699 | Brc1ccc(cc1)-c1oc(cc1)\C=C\1/SC=2N(C(C(C(OCC)=O)=C(N=2)C)c2ccccc2)C/1=O |
| 700 | Brc1cc(ccc1N(C)C)\C=C\1/SC=2N(C(C(C(OCC)=O)=C(N=2)C)c2ccc(OC)cc2)C/1=O |
| 701 | S\1C=2N(C(C(C(OCC)=O)=C(N=2)C)c2ccc(N(C)C)cc2)C(=O)/C/1=C/c1cc2OCOc2cc1 |
| 702 | S\1C=2N(C(C(C(OCC)=O)=C(N=2)C)c2ccc(N(C)C)cc2)C(=O)/C/1=C/c1c2c(cc3c1cccc3)cccc2 |
| 703 | Clc1ccc(cc1)C1N2C(S\C(=C/c3cc(OC)c(OCc4ccccc4)cc3)\C2=O)=NC(C)=C1C(OCC)=O |
| 704 | Clc1ccc(cc1)C1N2C(S\C(=C/c3ccccc3OCc3ccc(Cl)cc3)\C2=O)=NC(C)=C1C(OCC)=O |
| 705 | S\1C=2N(C(C(C(OCC)=O)=C(N=2)C)c2cc3OCOc3cc2)C(=O)/C/1=C/c1oc(cc1)-c1cc(ccc1)C(O)=O |
| 706 | Clc1cc(Cl)ccc1COc1ccccc1\C=C\1/SC=2N(C(C(C(OCC)=O)=C(N=2)C)c2cc(OC)c(OC)cc2)C/1=O |
| 707 | Clc1ccc(cc1)COc1ccc(cc1)\C=C\1/SC=2N(C(C(C(OCC)=O)=C(N=2)C)c2cc(OC)c(OC)cc2)C/1=O |
| 708 | S\1c2n(c3c(n2)c(C)c(cc3)C)C(=O)/C/1=C\c1ccc(SC)cc1 |
| 709 | Fc1ccc(Nc2nc(nc(n2)N\N=C\c2ccc(OC(=O)C)c(OC)c2[N+](=O)[O-])N2CCCCC2)cc1 |
| 710 | S\1\C(=C/c2ccc(OCC(O)=O)cc2)\C(=O)N(/C/1=N/c1ccccc1)c1ccccc1 |
| 711 | Brc1cc(ccc1)C(=O)N\N=C/1\Oc2c(C=C\1C(=O)N)cc(OC)cc2 |
| 712 | s1c2c(nc1NC(=O)\C=C\c1ccc(OC)cc1)cccc2 |
| 713 | Clc1cc(Cl)ccc1OCCCC(=O)Nc1sc(SCC)nn1 |
| 714 | Clc1nc2cc(OCC)ccc2cc1\C=N\N1C(=Nc2c(cccc2)C1=O)C |
| 715 | Brc1ccccc1C(OCCN1C(=O)c2c3c(cccc3c(N3CCCCC3)cc2)C1=O)=O |
| 716 | O1C(=N\C(=C/c2ccc(OCCCCCCCC)cc2)\C1=O)c1c(noc1C)-c1ccccc1 |
| 717 | S(C)c1nc2OC(Nc3c(-c2nn1)cccc3)c1ccc(cc1)C(C)(C)C |
| 718 | S(CCCC)c1nc2OC(Nc3c(-c2nn1)cccc3)c1ccccc1OCCC |
| 719 | S(CCCC)c1nc2OC(Nc3c(-c2nn1)cccc3)C(=O)c1ccccc1 |
| 720 | S=C1NC(=O)\C(=C\c2ccc(N(CCC)CCC)cc2)\C(=O)N1 |
| 721 | Ic1ccc(cc1)C=1OC(=O)/C(/N=1)=C/c1occc1 |
| 722 | Brc1oc(cc1)\C=C\1/N=C(OC/1=O)c1cc([N+](=O)[O-])c(cc1)C |
| 723 | Brc1ccccc1C=1OC(=O)/C(/N=1)=C/c1cc2OCOc2cc1Cl |
| 724 | Ic1ccccc1\C=C\1/N=C(OC/1=O)c1cc(Cl)ccc1 |
| 725 | O1C(=N\C(=C/c2cc(OC(=O)C)c(OC)cc2)\C1=O)c1ccccc1 |
| 726 | O1C(=N\C(=C/c2cc3c(cc2)cccc3)\C1=O)c1ccc(cc1)C(C)(C)C |
| 727 | Clc1cc(F)c(F)cc1C=1OC(=O)/C(/N=1)=C/c1ccccc1C |
| 728 | Clc1cc(F)c(F)cc1C=1OC(=O)/C(/N=1)=C/c1sccc1 |
| 729 | O1C(=N\C(=C/c2ccc(OCCCC)cc2)\C1=O)c1ccc(cc1)C |
| 730 | o1c2c(nc1-c1ccc(NC(=O)c3ccccc3OC)cc1)c1c(cc2)cccc1 |
| 731 | Brc1cc(-c2oc3c(n2)cc(NC(=O)c2cc(OCCC)ccc2)cc3)c(Cl)cc1 |
| 732 | Brc1ccc([N+](=O)[O-])cc1C(=O)Nc1cc2nc(oc2cc1)-c1cc2c(cc1)cccc2 |
| 733 | o1c2c(nc1-c1cc(NC(=O)c3ccccc3[N+](=O)[O-])ccc1)cc(cc2C)C |
| 734 | Brc1cc(-c2oc3c(n2)cc(NC(=O)c2cc(Cl)ccc2)cc3)c(Cl)cc1 |
| 735 | Clc1ccc(cc1[N+](=O)[O-])C(=O)Nc1cc(-c2oc3c(n2)cc(cc3)C)c(O)cc1 |
| 736 | Clc1ccc(cc1C(OCC)=O)-c1oc(cc1)\C=C/1\C(=O)NC(=O)NC\1=O |
| 737 | Clc1ccc(cc1C(OCCCC)=O)-c1oc(cc1)\C=C/1\C(=O)NC(=S)NC\1=O |
| 738 | Ic1cc(cc(OC)c1O)\C=C\1/C(=NN(C/1=O)c1cc(Cl)c(Cl)cc1)C |
| 739 | S=C1NC(=O)/C(/N1)=C\c1oc(cc1)-c1ccc([N+](=O)[O-])cc1OC |
| 740 | Brc1ccc(cc1)C=1OC(=O)/C(/N=1)=C/Nc1ccc(F)cc1 |
| 741 | O(C)c1ccc(cc1)C1=NN(C(C1)c1ccccc1)c1nc(c2cc(ccc2n1)C)-c1ccccc1 |
| 742 | S(CC(=O)N\N=C\c1ccc(N(C)C)cc1)C1=Nc2c(cccc2)C(=O)N1CC(C)=C |
| 743 | O\1c2c(C=C(C(=O)NC(=O)C)/C/1=N/c1ccc(OC)cc1)cccc2 |
| 744 | Clc1ccc(cc1)Cc1sc(nc1)NC(=O)CCN1C(=O)/C(/SC1=S)=C/c1ccc(F)cc1 |
| 745 | Brc1cc\2c(NC(=O)/C/2=C/2\SC(=S)N(C\2=O)c2cc(ccc2)C(F)(F)F)cc1 |
| 746 | S1\C(=C/c2ccc(OC)cc2)\C(=O)N(CCCC(=O)Nc2ccccc2C(O)=O)C1=S |
| 747 | Brc1ccc(cc1)C=1OC(=O)/C(/N=1)=C/Nc1cc(Cl)ccc1C |
| 748 | Clc1ccc(cc1)C=1OC(=O)/C(/N=1)=C/Nc1cc([N+](=O)[O-])ccc1C |
| 749 | Clc1ccc(cc1)C=1OC(=O)/C(/N=1)=C/Nc1sc2cc(OCC)ccc2n1 |
| 750 | s1ccc(C)c1\C=C\1/N=C(OC/1=O)c1cc([N+](=O)[O-])c(OC)cc1 |
| 751 | Clc1c2n(nc1C(=O)Nc1cc(Oc3ccc(F)cc3)cc(Oc3ccc(F)cc3)c1)C(=CC(=N2)c1sccc1)C(F)(F)F |
| 752 | O1C(=O)/C(/N=C1\C=C\c1cc([N+](=O)[O-])ccc1)=C\Nc1ccc(cc1)C |
| 753 | Brc1cc(\C=C/2\N=C(OC\2=O)c2cc([N+](=O)[O-])c(cc2)C)c(OC)cc1 |
| 754 | O1C(=N\C(=C\c2ccc(OCC)cc2)\C1=O)c1ccc(cc1)C(C)(C)C |
| 755 | Clc1ccc([N+](=O)[O-])cc1\C=N\NC(=O)C(NC(=O)c1ccccc1)C1=NNC(=O)c2c1cccc2 |
| 756 | Clc1cccc(NCN2C(=O)/C(/SC2=S)=C\c2ccc(OC)cc2)c1C |
| 757 | Ic1cc(cc(OC)c1OCC#C)\C=C/1\Sc2n(c3cc(C)c(cc3n2)C)C\1=O |
| 758 | o1nc2nc(Nc3ccc(cc3)C(OCC)=O)c(nc2n1)N\N=C\c1c[nH]nc1-c1ccccc1 |
| 759 | S\1c2c(cccc2)C(=O)/C/1=C/c1cc(OC)c(OCC#N)cc1 |
| 760 | O(C)c1ccc(OC)cc1\C=N\Nc1nc(cc(COC)c1C#N)C |
| 761 | Brc1cc(\C=N\Nc2nc(cc(COC)c2C#N)C)c(O)c(OC)c1 |
| 762 | Brc1c(COC)c(C#N)c(OCC(=O)N\N=C\c2cc(Br)c(OC)c(Br)c2)nc1C |
| 763 | Brc1c(COC)c(C#N)c(OCC(=O)N\N=C\c2cc(OC)c(OCc3ccccc3)cc2)nc1C |
| 764 | S1\C(=C/c2cc(OC)c(Oc3ncc([N+](=O)[O-])cc3)cc2)\C(=O)NC1=S |
| 765 | Ic1ccc(NCN2C(=O)/C(/SC2=O)=C\c2ccc(F)cc2)cc1 |
| 766 | S1\C(=C/c2oc(cc2)-c2cc(C)c([N+](=O)[O-])cc2)\C(=O)NC1=S |
| 767 | S1\C(=C/c2oc(cc2)-c2cc(C)c([N+](=O)[O-])cc2)\C(=O)N(c2ccccc2)C1=S |
| 768 | Clc1cc(N2C(=O)/C(/SC2=S)=C/c2oc(cc2)-c2ccccc2[N+](=O)[O-])ccc1 |
| 769 | S1\C(=C/c2oc(cc2)-c2cc(C)c([N+](=O)[O-])cc2)\C(=O)N(c2ccc(OC)cc2)C1=S |
| 770 | Clc1c(cccc1Cl)C(=O)Nc1ccc(cc1)-c1oc2cccnc2n1 |
| 771 | Ic1ccc(Cl)cc1C(=O)Nc1cc2nc(oc2cc1)-c1ccc(F)cc1Cl |
| 772 | Brc1cc(-c2oc3c(n2)cc(NC(=O)c2ccc(Br)cc2)cc3)c(Cl)cc1 |
| 773 | Brc1cc(C(=O)Nc2cc(ccc2)-c2oc3cccnc3n2)c(Cl)cc1 |
| 774 | Brc1cc(C(=O)Nc2cc3nc(oc3cc2)-c2cc(Cl)c(Cl)cc2)c(Cl)cc1 |
| 775 | Clc1ccc([N+](=O)[O-])cc1C(=O)Nc1ccc(cc1)-c1oc2c(n1)cccc2 |
| 776 | s1c2c(nc1-c1cc(NC(=O)c3ccccc3C)c(cc1)C)cccc2 |
| 777 | S1\C(=C/c2occc2)\C(=O)N(Cc2ccccc2)C1=O |
| 778 | S1\C(=C/c2ccc(cc2)C(OC)=O)\C(=O)N(c2cccc(C)c2C)C1=S |
| 779 | FC(F)(F)c1cc(ccc1)\C=C/1\N=C(OC\1=O)c1ccccc1C |
| 780 | Clc1cc(ccc1Cl)C=1OC(=O)/C(/N=1)=C\c1cc(OC(=O)C)c(OC)cc1 |
| 781 | O1C(=N\C(=C/c2cc(OC)c(OC)c(OC)c2)\C1=O)c1cc(ccc1)C |
| 782 | Clc1ccc(cc1)\C=C\1/N=C(OC/1=O)c1ccc(OCCCC)cc1 |
| 783 | O1C(=N\C(=C/c2cc(OC(=O)C)ccc2)\C1=O)c1ccc(OCCCCCCCCCC)cc1 |
| 784 | s1cc(nc1NC(=O)c1ccccc1Oc1ccccc1)-c1ccc(cc1)C |
| 785 | s1cc(nc1NC(=O)c1ccc(S(=O)(=O)N(CCOC)CCOC)cc1)-c1ccc([N+](=O)[O-])cc1 |
| 786 | S\1C=2N(C(C(C(OCC)=O)=C(N=2)C)c2ccc(OC(=O)C)cc2)C(=O)/C/1=C\c1ccc(cc1)C |
| 787 | S(CC(OCCCC)=O)C=1NC(=O)CC(C=1C#N)c1ccccc1 |
| 788 | Clc1ccc(cc1)C1CC(=O)NC(SCC(OCCC)=O)=C1C#N |
| 789 | s1cc(nc1/C(=C\c1cc(OC)ccc1OC)/C#N)C1=Cc2c(OC1=O)cccc2 |
| 790 | s1cc(nc1C=1C(=O)c2cc(CCC)c(OC)cc2OC=1N)C |
| 791 | O1C=C(C(=O)c2c1cc(OC)cc2)c1nocc1 |
| 792 | O1c2c(cc(CC)c(OC(C)C)c2)C(=O)C(c2cc3OCCCOc3cc2)=C1C(C)C |
| 793 | O1C=C(C(=O)c2cc(CC)c(OC)cc12)c1nc2c(cc1)cccc2 |
| 794 | O1C=C(C(=O)c2cc(CCC)c(OC(=O)C)cc12)c1cc2OCCOc2cc1 |
| 795 | S1\C(=C/c2ccccc2)\C(=O)N(CCCCCC(OC)=O)C1=S |
| 796 | S1\C(=C/c2cc(OC)c(OC)cc2)\C(=O)N(NC(=O)c2cc([N+](=O)[O-])ccc2)C1=S |
| 797 | S1\C(=C/c2ccc([N+](=O)[O-])cc2)\C(=O)N(NC(=O)c2ccccc2O)C1=S |
| 798 | O(CC)c1cc(ccc1O)C1NC(=O)NC(C)=C1C(OC1CCCCCCC1)=O |
| 799 | O(C(C)C)c1ccccc1C1C2=C(NC(C)=C1C(OCCOc1ccccc1)=O)CC(CC2=O)(C)C |
| 800 | Clc1ccc(cc1)\C=C\1/N=C(OC/1=O)c1ccccc1 |
| 801 | Clc1cc(NC(=O)C2S\C(=N\c3ccccc3OC)\N(C)C(=O)C2)ccc1 |
| 802 | S1\C(=N\c2ccccc2OC)\N(C)C(=O)CC1C(=O)Nc1ccc(cc1)C(O)=O |
| 803 | Clc1ccc(\N=C\2/SC(CC(=O)N/2CCc2cc(OC)c(OC)cc2)C(=O)Nc2ccc(cc2C)C)cc1 |
| 804 | Clc1ccc(cc1)CN1/C(/SC(CC1=O)C(=O)Nc1ccc(OC)cc1)=N/c1ccccc1 |
| 805 | S1\C(=N/c2cc(OC)ccc2)\N(C)C(=O)CC1C(=O)Nc1ccc(cc1)C(O)=O |
| 806 | O=C(N(CC)CC)CN(C#N)c1nc(cc(n1)C)C |
| 807 | O(C)c1cc2c(n(C)c(C)c2\C=C\C2=Nc3c(cccc3)C(=O)N2c2ccccc2C)cc1 |
| 808 | ClC=1CC2C(CC=1)C(=O)N(CCO)C2=O |
| 809 | S1(=O)(=O)N(CN(C#N)c2nc(cc(n2)C)C)C(=O)c2c1cccc2 |
| 810 | Clc1c2n(nc1C(=O)NCCc1ccccc1)C(=CC(=N2)c1ccccc1)C(F)(F)F |
| 811 | Clc1c2n(nc1C(=O)NCCCn1ccnc1)C(=CC(=N2)c1sccc1)C(F)(F)F |
| 812 | Fc1ccc(cc1)C1=Nc2n(ncc2C(=O)NCC2OCCC2)C(=C1)C(F)(F)F |
| 813 | FC(F)(F)C=1n2ncc(c2N=C(C=1)c1ccc(OC)cc1)C(=O)NC12CC3CC(C1)CC(C2)C3 |
| 814 | s1cccc1CNC(=O)c1c2n(nc1)C(=CC(=N2)c1cc(OC)c(OC)c(OC)c1)C(F)(F)F |
| 815 | Clc1c(n(nc1C(F)(F)F)CC(=O)Nc1cc(C)c(cc1)C)C |
| 816 | Clc1c(n(nc1C(F)(F)F)CC(=O)Nc1cc(OC)c(Cl)cc1OC)C |
| 817 | Clc1c2n(nc1C(=O)NCCCN1CCOCC1)C(=CC(=N2)c1ccc(Cl)cc1)C(F)(F)F |
| 818 | Brc1c2n(nc1C(=O)Nc1cc([N+](=O)[O-])cc(OC)c1)C(=CC(=N2)c1oc(Br)cc1)C(F)(F)F |
| 819 | Clc1ccc(cc1[N+](=O)[O-])C(=O)Nc1ccc(cc1)-c1oc2c(n1)cccc2 |
| 820 | Clc1ccc(cc1[N+](=O)[O-])C(=O)Nc1cc2nc(oc2cc1)-c1ccncc1 |
| 821 | O(C)c1ccc(cc1)C1CC(=O)C2=C(NC(C)=C(C(OCCc3ccccc3)=O)C2c2ccc(N(CC)CC)cc2)C1 |
| 822 | s1c(C(=O)Nc2ccccc2C)c(C)c(C(OCC)=O)c1NC(=O)c1ccc(F)cc1 |
| 823 | S1\C(=C/c2sccc2)\C(=O)N(NC(=O)c2ccc(cc2)C)C1=S |
| 824 | Clc1cc2c(NC(=O)C(=C2c2ccccc2)C2=NN(C(=O)C)C(C2)c2ccccc2F)cc1 |
| 825 | Brc1cc2c(nc(Oc3ccc(OC)cc3)nc2-c2ccccc2)cc1 |
| 826 | Clc1cc(ccc1OCC)C(=O)Nc1cc(ccc1C)-c1oc2cccnc2n1 |
| 827 | Clc1ccc(OCC(=O)Nc2ccc(cc2)-c2oc3cccnc3n2)cc1C |
| 828 | Brc1cc(ccc1OCC(O)=O)\C=C/1\C(=O)NC(=O)NC\1=O |
| 829 | O(C)c1cc(\C=C\2/C(=NN(C/2=O)c2ccc(cc2)C(O)=O)C)c([N+](=O)[O-])cc1OC |
| 830 | FC(F)(F)c1cc(ccc1)C1C(C(OC)=O)=C(N(C)C(C)=C1C(OC)=O)C |
| 831 | O(C)c1ccccc1NC(=O)C=1C(NC(=O)NC=1C)c1cc(OC)c(O)cc1 |
| 832 | Clc1cc2OCOc2cc1C1C2=C(NC(C)=C1C(OC1CCCC1)=O)CC(CC2=O)c1ccc(Cl)cc1 |
| 833 | S(CC(OCC)=O)C1=NC2=C(C3(CCCC3)Cc3c2cccc3)C(=O)N1c1ccccc1 |
| 834 | S(CCOc1ccc(cc1)C(C)(C)C)c1nc2c(n1CC(O)=O)cccc2 |
| 835 | Fc1ccc(cc1)C(=O)N1N=C(CC1c1ccc(N(C)C)cc1)c1ccccc1 |
| 836 | O(CC#N)c1cc(ccc1OC)\C=C\1/C(=O)N(c2ccc(OC)cc2)C(=O)NC/1=O |
| 837 | Clc1cc(cc(Cl)c1OCc1ccc(cc1)C(O)=O)\C=C/1\C(=O)N(c2ccc(OCC)cc2)C(=O)NC\1=O |
| 838 | Ic1cc(cc(I)c1OCc1ccc(cc1)C(O)=O)\C=C/1\C(=O)N(c2ccccc2)C(=O)NC\1=O |
| 839 | S=C1NC(=O)\C(=C\c2cc(OC)c(OCc3ccc(cc3)C(O)=O)cc2)\C(=O)N1 |
| 840 | s1c2cc(NC(=O)CSc3sc4cc(OCC)ccc4n3)ccc2nc1SCC(=O)NC1CC1 |
| 841 | S(CC(OC)=O)c1nc2c(CCC2)c(C2CCC=CC2)c1C#N |
| 842 | s1ccnc1NC(=O)c1ccc(NC(=O)Cc2ccccc2)cc1 |
| 843 | Clc1c2c(sc1C(=O)N1c3c(CCc4c1cccc4)cccc3)cccc2 |
| 844 | O=C1NC(=NC1CC(O)=O)Nc1nc(c2cc(ccc2n1)C)C |
| 845 | Clc1ccc(cc1)C1CC(=O)C2=C(NC(C)=C(C(OC3CCCC3)=O)C2c2cc(O)ccc2)C1 |
| 846 | O(CCOc1ccc(cc1)\C=C\1/C(=O)N(c2ccc(cc2)C)C(=O)NC/1=O)c1ccc(cc1)C(CC)C |
| 847 | O(CCOc1ccc(cc1)C)c1ccc(cc1OC)\C=C/1\C(=O)N(C)C(=O)N(C)C\1=O |
| 848 | O(CCOc1ccc([N+](=O)[O-])cc1)c1cc(ccc1)\C=C/1\C(=O)N(C)C(=O)N(C)C\1=O |
| 849 | S(c1ccc(cc1)C)c1oc(cc1)\C=C/1\C(=O)NC(=O)NC\1=O |
| 850 | Clc1ccc(cc1)C(=O)CSC1=Nc2sc3CCCCc3c2C(=O)N1CC |
| 851 | s1c2CCCCc2c2c1N=C(SCc1ccc(cc1C)C)N(CC=C)C2=O |
| 852 | S(=O)(=O)(N(NC1=NS(=O)(=O)c2c1cccc2)C(=O)CCC)c1ccc(cc1)C |
| 853 | Clc1cc(cc(OC)c1O)C1C2=C(NC(C)=C1C(OC(C)C)=O)CC(CC2=O)c1ccccc1 |
| 854 | s1c(C(=O)Nc2ccccc2F)c(C)c(C(OCC)=O)c1NC(=O)c1cc([N+](=O)[O-])c(cc1)C |
| 855 | O(C)c1nc(nc(n1)NCC=C)C |
| 856 | S(=O)(=O)(Nc1nc(nc(n1)NCC)NC(C)C)c1ccc(cc1)C |
| 857 | O(c1ccc(cc1)C(OC)=O)c1nc(nc(n1)NCC)NCC |
| 858 | O(C)c1cc(ccc1OC)C1C(C(OC)=O)=C(N(C)C(C)=C1C(OC)=O)C |
| 859 | O1CCCC1COC(=O)C=1C(C2=C(NC=1C)CC(CC2=O)(C)C)C1=COc2c(cccc2)C1=O |
| 860 | O=C1N(NC(=O)c2ccccc2[N+](=O)[O-])C(=Nc2c1cccc2)C |
| 861 | O=C1N(NC(=O)c2cc(C)c(cc2)C)C(=Nc2c1cccc2)c1ccc(cc1)C |
| 862 | s1cc(nc1N1N=C(CC1c1occc1)c1ccc(OC)cc1)-c1cc(N2C(=O)c3c(cccc3)C2=O)ccc1 |
| 863 | s1cc(nc1N1N=C(CC1c1ccc(F)cc1)c1cc([N+](=O)[O-])ccc1)-c1ccc(S(=O)(=O)N2CCCC2)cc1 |
| 864 | Clc1ccc(cc1S(=O)(=O)N1CCOCC1)C(=O)NCc1ccccc1 |
| 865 | S(=O)(=O)(N1CCN(CC1)c1ccccc1)c1cc(ccc1)C(=O)N1CCCc2c1cccc2 |
| 866 | s1c(nnc1NC(=O)C(Sc1nc(cc(n1)C)C)CC)C |
| 867 | Clc1ccc(cc1)C1CC(=O)C2=C(NC(C)=C(C(OCCc3ccccc3)=O)C2c2cc(OC)c(O)cc2)C1 |
| 868 | Oc1cc(ccc1)C1NC(=O)NC(C)=C1C(OCc1ccccc1)=O |
| 869 | o1c2cccnc2nc1-c1cc(NC(=O)c2cc(OC)cc(OC)c2)ccc1 |
| 870 | o1nc(CC)c(C)c1-c1[nH]c2c(cccc2)c1C(=O)C(=O)N(C)C |
| 871 | O=C1N(C(=O)C2C1CC(CC2)C)c1cc(ccc1)C |
| 872 | O(CC)c1ccc(cc1)C1N(c2c(NC3=C1C(=O)CC(C3)(C)C)cccc2)C(=O)CCCC |
| 873 | Clc1ccc(cc1)C1N(c2c(NC3=C1C(=O)CC(C3)(C)C)cccc2)C(=O)CC(C)C |
| 874 | O(CCC)c1cc(ccc1)C1Nc2c(NC3=C1C(=O)CC(C3)(C)C)cccc2 |
| 875 | o1c(ccc1-c1cc([N+](=O)[O-])ccc1)C1N(c2c(NC3=C1C(=O)CC(C3)(C)C)cccc2)C(=O)c1ccccc1 |
| 876 | Oc1ccccc1\C=C/C1=Nc2c(cccc2)C(=O)N1c1ccc(cc1)C(O)=O |
| 877 | O1C=C(Oc2ccc(OC)cc2)C(=O)c2c1c(CN1CCOCC1)c(O)cc2 |
| 878 | Clc1ccc(cc1)C1CC(=O)C2=C(NC(C)=C(C(OC(C)C)=O)C2c2ccc(N(CC)CC)cc2)C1 |
| 879 | Fc1ccccc1C1=Nc2c(cccc2)C(=O)N1NC(=O)c1ccc(cc1)-c1cc(nc2c1cccc2)-c1ccccc1 |
| 880 | O(C)c1c(OC)cc(cc1OC)C1=Nc2c(cccc2)C(=O)N1NC(=O)c1ccc(cc1)CCCC |
| 881 | S(CC#N)C1=Nc2c(ccc(c2)C(=O)N2CCCC2)C(=O)N1CCCCC |
| 882 | S(CC(=O)c1ccccc1)C1=Nc2c(ccc(c2)C(=O)N2CCN(CC2)c2ccccc2)C(=O)N1CCCCC |
| 883 | s1c2N=C(SCC(=O)c3ccccc3)N(Cc3ccccc3)C(=O)c2c(C)c1C(=O)NC1CCCCC1 |
| 884 | Clc1ccc(N2C=3N=C(SCc4cc([N+](=O)[O-])ccc4)N(C(=O)C=3SC2=S)c2ccccc2)cc1 |
| 885 | S1C2=C(N=C(SCCN3CCOCC3)N(CCc3ccccc3)C2=O)N(c2c(cccc2C)C)C1=S |
| 886 | Brc1ccc(N2C(=O)C=3SC(=S)N(C=3N=C2SCC#N)c2c(cccc2C)C)cc1 |
| 887 | S1C2=C(N=C(SCc3ccncc3)N(c3ccccc3OC)C2=O)N(Cc2ccccc2)C1=S |
| 888 | Clc1cc(ccc1)CNC(=O)c1cc2NC(=S)N(C3CCCCC3)C(=O)c2cc1 |
| 889 | Clc1ccccc1CNC(=O)c1cc2NC(=S)N(CCc3ccccc3)C(=O)c2cc1 |
| 890 | S=C1Nc2c(ccc(c2)C(=O)NCCC=2CCCCC=2)C(=O)N1c1ccccc1F |
| 891 | S=C1Nc2c(ccc(c2)C(=O)NCc2ccccc2)C(=O)N1c1cc(OC)c(OC)cc1 |
| 892 | S=C1Nc2c(ccc(c2)C(=O)N2CCN(CC2)c2ccccc2)C(=O)N1Cc1ccc(OC)cc1 |
| 893 | S=C1Nc2c(ccc(c2)C(=O)Nc2ccc(OC)cc2)C(=O)N1c1ccccc1 |
| 894 | S=C1Nc2c(ccc(c2)C(=O)NCC2OCCC2)C(=O)N1C1CCCCC1 |
| 895 | S=C1Nc2c(ccc(c2)C(=O)NCCCN2CCOCC2)C(=O)N1CCCCC |
| 896 | S=C1Nc2c(ccc(c2)C(=O)NC2CCCC(C)C2C)C(=O)N1CCc1ccccc1 |
| 897 | S=C1Nc2c(ccc(c2)C(=O)Nc2c3c(ccc2)cccc3)C(=O)N1c1cc(OC)ccc1OC |
| 898 | Clc1cc(N2C(=O)c3c(NC2=S)cc(cc3)C(=O)NC2CCCC(C)C2C)ccc1F |
| 899 | S=C1Nc2c(ccc(c2)C(=O)Nc2cc(OC)c(OC)c(OC)c2)C(=O)N1Cc1occc1 |
| 900 | S=C1Nc2c(ccc(c2)C(=O)NCCSCc2ccccc2)C(=O)N1c1ccc(F)cc1 |
| 901 | S=C1Nc2c(ccc(c2)C(=O)NCc2occc2)C(=O)N1c1cc(OC)c(OC)cc1 |
| 902 | Clc1ccc(cc1N1C(=O)c2c(NC1=S)cc(cc2)C(=O)NCc1occc1)C(F)(F)F |
| 903 | S=C1Nc2c(ccc(c2)C(=O)Nc2cc3CCCc3cc2)C(=O)N1Cc1ccc(OC)cc1 |
| 904 | S=C1Nc2c(ccc(c2)C(=O)Nc2cc3CCCc3cc2)C(=O)N1c1ccc(OC)cc1 |
| 905 | s1c(nnc1NC(=O)CSc1nnc(n1-c1ccccc1OC)CNC(=O)c1ccc(S(=O)(=O)N(C)C)cc1)C |
| 906 | s1cccc1C(=O)NCc1nnc(SCC(=O)Nc2sccn2)n1-c1ccccc1OC |
| 907 | S(CC(=O)Nc1ccc(F)cc1)c1nnc(n1-c1ccc(OCC)cc1)CNC(=O)c1ccc(S(=O)(=O)N(C)C)cc1 |
| 908 | S(CC(=O)N1CCc2c1cccc2)c1nnc(n1-c1ccccc1OC)CNC(=O)c1cc(OC)ccc1 |
| 909 | s1ccnc1NC(=O)CSc1nnc(n1-c1cc(OC)ccc1OC)CNC(=O)c1cc(OC)cc(OC)c1 |
| 910 | s1ccnc1NC(=O)CSc1nnc(n1-c1ccc(OCC)cc1)CNC(=O)c1ccc(S(=O)(=O)N(CC)CC)cc1 |
| 911 | s1ccnc1NC(=O)CSc1nnc(n1-c1cc(OC)ccc1OC)CNC(=O)c1occc1 |
| 912 | Brc1ccc(N2C(=O)C=3SCCC=3N=C2SCC(=O)N2CCC(CC2)Cc2ccccc2)cc1 |
| 913 | S1C2=C(N=C(SCC(=O)N3CCCc4c3cccc4)N(C2=O)c2ccc(OCC)cc2)CC1 |
| 914 | S1C(C(=O)NCCCC)=C(N)N(c2c(cccc2C)C)C1=S |
| 915 | S=C1Nc2c(ccc(c2)C(O)=O)C(=O)N1c1cc(ccc1)C(F)(F)F |
| 916 | S(CC(=O)N1N=C(CC1c1cccc(OC)c1OC)c1ccc(OC)cc1)c1nnc(n1-c1ccccc1)CNC(=O)c1ccccc1 |
| 917 | Brc1ccc(cc1)C1=NN(C(=O)CSc2nnc(n2C)CNC(=O)c2ccccc2)C(C1)c1ccc(cc1)C |
| 918 | s1cccc1C1=NN(C(=O)CSc2nnc(n2-c2cccc(C)c2C)CNC(=O)c2ccccc2F)C(C1)c1cccc(OC)c1OC |
| 919 | S(CC(=O)N1N=C(CC1c1ccc(OC)cc1)c1ccc(OC)cc1)c1nnc(n1-c1ccc([N+](=O)[O-])cc1)CNC(=O)c1cc(F)ccc1 |
| 920 | s1cccc1C1=NN(C(=O)CSc2nnc(n2-c2ccc(F)cc2)CNC(=O)c2ccc([N+](=O)[O-])cc2)C(C1)c1ccc(F)cc1 |
| 921 | s1cccc1C1=NN(C(=O)CSc2nnc(n2-c2ccc(F)cc2)CNC(=O)c2ccccc2OC)C(C1)c1ccc(cc1)C |
| 922 | s1cccc1C1=NN(C(=O)CSc2nnc(n2-c2ccccc2)CNC(=O)c2ccc(OC)cc2)C(C1)c1ccc(OC)cc1 |
| 923 | s1cccc1C1=NN(C(=O)CSc2nnc(n2C)CNC(=O)c2ccc(OC)cc2)C(C1)c1ccc(F)cc1 |
| 924 | s1cccc1C1=NN(C(=O)CSc2nnc(n2-c2ccc(F)cc2)CNC(=O)c2cccc([N+](=O)[O-])c2C)C(C1)c1ccc(F)cc1 |
| 925 | s1cccc1C1=NN(C(=O)CSc2nnc(n2-c2ccc(F)cc2)CNC(=O)c2cc(OC)c(OC)cc2)C(C1)c1ccc(OC)cc1 |
| 926 | s1cccc1C1=NN(C(=O)CSc2nnc(n2C)CNC(=O)c2ccc(S(=O)(=O)N3CCCC3)cc2)C(C1)c1ccc(cc1)C |
| 927 | S(CC(=O)N1N=C(CC1c1ccc(F)cc1)c1ccc(OC)cc1)c1nnc(n1C)CNC(=O)Cc1ccccc1 |
| 928 | s1cccc1C1=NN(C(=O)CSc2nnc(n2-c2cc(ccc2)C)CNC(=O)COc2ccccc2)C(C1)c1cccc(OC)c1OC |
| 929 | S(CC(=O)N1N=C(CC1c1ccc(F)cc1)c1ccc(OC)cc1)c1nnc(n1-c1cc(ccc1)C(F)(F)F)CNC(=O)c1occc1 |
| 930 | S(CC(=O)N1N=C(CC1c1ccc(F)cc1)c1ccc(OC)cc1)c1nnc(n1-c1ccc(OCC)cc1)CNC(=O)c1occc1 |
| 931 | s1cccc1C(=O)NCc1nnc(SCC(=O)N2N=C(CC2c2ccc(OC)cc2)c2ccc(OC)cc2)n1-c1ccccc1 |
| 932 | s1cccc1C(=O)NCc1nnc(SCC(=O)N2N=C(CC2c2cccc(OC)c2OC)c2ccc(OC)cc2)n1C |
| 933 | s1cccc1C1=NN(C(=O)CSc2nnc(n2Cc2ccccc2)CNC(=O)Cc2ccccc2)C(C1)c1ccc(cc1)C |
| 934 | S(CC(=O)N1N=C(CC1c1cccc(OC)c1OC)c1ccc(OC)cc1)c1nnc(n1Cc1ccccc1)CNC(=O)c1occc1 |
| 935 | s1cccc1C1=NN(C(=O)CSc2nnc(n2CCc2ccccc2)CNC(=O)c2cc(ccc2)C)C(C1)c1ccc(F)cc1 |
| 936 | Fc1cc2N=C(c3ccccc3N)C(=O)Nc2cc1 |
| 937 | Clc1cc(N2N=C(CC2=O)c2ccccc2)ccc1 |
| 938 | O\1c2cc(N(CC)CC)ccc2C=C(C(=O)N)/C/1=N/c1ccc(cc1)C(=O)c1ccccc1 |
| 939 | Clc1cc(Cl)c2c(nc(N\N=C\c3cc4CCCN5CCCc(c45)c3O)cc2)c1O |
| 940 | s1cccc1C1=Nc2n(nc(c2)C(=O)Nc2c3nsnc3ccc2)C(=C1)C(F)(F)F |
| 941 | Brc1c(n(nc1[N+](=O)[O-])CC(=O)N1CCOCC1)C |
| 942 | Clc1c2n(nc1C(=O)N1CCCCC1)C(=CC(=N2)c1sccc1)C(F)(F)F |
| 943 | Clc1ccc(NC(=O)c2c3n(nc2)C(=CC(=N3)c2cc(OC)c(OC)cc2)C(F)(F)F)cc1 |
| 944 | FC(F)(F)C=1n2nc(cc2N=C(C=1)c1ccc(OC)cc1)C(=O)N1CCN(CC1)C(c1ccccc1)c1ccccc1 |
| 945 | s1c2CC(CCc2c(C#N)c1NC(=O)c1nn2c(N=C(C=C2C(F)(F)F)c2ccccc2)c1)C |
| 946 | Clc1c2n(nc1C(=O)Nc1sc3CC(CCc3c1C#N)C)C(=CC(=N2)c1sccc1)C(F)(F)F |
| 947 | s1cccc1C1=Nc2n(nc(c2)C(=O)NCCc2cc(OC)c(OC)cc2)C(=C1)C(F)(F)F |
| 948 | s1c2c(CCCC2)c(C(OC)=O)c1NC(=O)Cn1nc([N+](=O)[O-])nc1 |
| 949 | Brc1ccc(cc1)C1=Nc2n(nc(c2)C(=O)Nc2ccc(Cl)cc2)C(=C1)C(F)(F)F |
| 950 | Brc1ccc(cc1)C1=Nc2n(nc(c2)C(=O)Nc2cc([N+](=O)[O-])cc(Oc3ccccc3)c2)C(=C1)C(F)(F)F |
| 951 | Brc1ccc(cc1)C1=Nc2n(nc(C(=O)N)c2Cl)C(=C1)C(F)(F)F |
| 952 | Brc1ccc(cc1)C1=Nc2n(nc(C(=O)Nc3sc4CC(CCc4c3C#N)C(C)(C)C)c2Cl)C(=C1)C(F)(F)F |
| 953 | Brc1c2n(nc1C(=O)NCCOC)C(=CC(=N2)c1ccc(Br)cc1)C(F)(F)F |
| 954 | Clc1cc(ccc1Cl)C1=Nc2n(nc(c2)C(=O)N2CCOCC2)C(=C1)C(F)(F)F |
| 955 | O1C(=N\C(=C/c2cc(OC(=O)C)ccc2)\C1=O)c1cc(OC)c(OC)c(OC)c1 |
| 956 | Brc1cc(sc1)\C=C/1\N=C(OC\1=O)c1oc(Br)cc1 |
| 957 | O1C(=N/C(=C\C(=C\c2ccccc2)\C)/C1=O)c1ccc(cc1)C(C)(C)C |
| 958 | Brc1ccc(cc1)\C=C\1/N=C(OC/1=O)c1c(noc1C)-c1ccccc1Cl |
| 959 | O1C(=N\C(=C/c2ccc(OC)cc2)\C1=O)C1CCC(CC1)CCC |
| 960 | O1C(=N\C(=C\c2cc(OC)ccc2)\C1=O)c1ccc([N+](=O)[O-])cc1 |
| 961 | O=C1N(CNc2cc(ccc2C)C)C(=O)C2C1CC=CC2 |
| 962 | Ic1oc(cc1)\C=C\1/N=C(OC/1=O)c1ccccc1C |
| 963 | Clc1cc(ccc1)C=1OC(=O)/C(/N=1)=C/c1ccc([N+](=O)[O-])cc1 |
| 964 | Brc1ccccc1\C=C\1/N=C(OC/1=O)c1ccc(Br)cc1 |
| 965 | O1C(=N\C(=C/c2ccc(OC(=O)C)cc2)\C1=O)c1ccc(cc1)C(C)(C)C |
| 966 | Clc1ccc(cc1[N+](=O)[O-])C=1OC(=O)/C(/N=1)=C/c1cc2c(cc1)cccc2 |
| 967 | O1C(=N\C(=C/c2ccccc2C)\C1=O)c1occc1 |
| 968 | Clc1ccc(cc1[N+](=O)[O-])C=1OC(=O)/C(/N=1)=C/c1ccc(cc1)C |
| 969 | Brc1cc(cc(OCC)c1OC(=O)C)\C=C\1/N=C(OC/1=O)c1ccccc1 |
| 970 | Ic1cc(cc(OCC)c1OC(=O)C)\C=C\1/N=C(OC/1=O)c1cc(Br)ccc1 |
| 971 | o1c2c(nc1-c1cccc(NC(=O)c3ccc(OCCC)cc3)c1C)cc(cc2)C |
| 972 | S(CCOc1ccc(OCC)cc1)c1nc2c(n1CCOc1ccccc1F)cccc2 |
| 973 | o1c2cc(ccc2nc1-c1cc(NC(=O)c2ccccc2[N+](=O)[O-])c(cc1)C)C |
| 974 | Brc1cc(-c2oc3c(n2)cc(NC(=O)c2ccc(Cl)cc2Cl)cc3)c(Cl)cc1 |
| 975 | o1c2c(nc1-c1ccccc1)cc(NC(=O)c1cc(OC)c(OC)c(OC)c1)cc2 |
| 976 | Brc1cc(Br)cc(\C=C/2\C(=NN(C\2=O)c2ccccc2)C)c1OC |
| 977 | Clc1cc(N2N=C(C)\C(=C\c3ccc(OCc4ccccc4)cc3)\C2=O)ccc1 |
| 978 | Clc1cc(N2N=C(C)\C(=C/c3cc(OC)ccc3)\C2=O)ccc1Cl |
| 979 | O(C)c1cc(N(CC)CC)ccc1\C=C/1\NC(=O)NC\1=O |
| 980 | O1C(=O)/C(/N=C1\C=C\c1ccccc1OC)=C/c1cccnc1 |
| 981 | Brc1cc(ccc1)C1N(N=C(C1)c1ccc(Br)cc1)C(=O)c1ccc(Br)cc1 |
| 982 | S1\C(=C/Nc2ccccc2)\C(=O)N(C(C(O)=O)C)C1=S |
| 983 | Brc1cc2C=C(c3nc(sc3)Nc3ccc(OC)cc3)C(Oc2cc1)=O |
| 984 | S1\C(=C/c2cc(OC)c(OC)c(OC)c2)\C(=O)N(c2cc(ccc2)C(F)(F)F)C1=S |
| 985 | S1\C(=C/c2ccccc2[N+](=O)[O-])\C(=O)N(c2ccc(N(CC)CC)cc2)C1=S |
| 986 | S1\C(=C/c2ccc(OC)cc2)\C(=O)N(CCCC(=O)Nc2cc(ccc2)C(O)=O)C1=S |
| 987 | S1\C(=C/c2ccc(N(C)C)cc2)\C(=O)N(NC(=O)c2ccccc2[N+](=O)[O-])C1=S |
| 988 | Clc1ccc(cc1)C=1OC(=O)/C(/N=1)=C/Nc1ccc(O)cc1 |
| 989 | Clc1ccc([N+](=O)[O-])cc1N\C=C\1/N=C(OC/1=O)c1ccc(Cl)cc1 |
| 990 | s1ccc(C)c1\C=C\1/N=C(OC/1=O)c1cc(OC)ccc1 |
| 991 | Clc1cc(ccc1)C1NC(=O)NC(C)=C1C(OC1CCCC1)=O |
| 992 | O1C(=N\C(=C\c2ccccc2OCC)\C1=O)c1cc([N+](=O)[O-])ccc1 |
| 993 | FC(F)(F)c1cc(ccc1)\C=C/1\N=C(OC\1=O)c1cc([N+](=O)[O-])ccc1 |
| 994 | Clc1cc(C=2OC(=O)/C(/N=2)=C\c2ccc(OC)c(OC)c2OC)c(OC)cc1 |
| 995 | Brc1cc(cc(OC)c1O)\C=C/1\C(=O)N(C)C(=O)N(C)C\1=O |
| 996 | S1(=O)(=O)N=C(NCC(O)CN(CC)CC)c2c1cccc2 |
| 997 | S\1\C(=C/c2cc(OC)c(Oc3ccc(cc3[N+](=O)[O-])C(F)(F)F)cc2)\C(=O)N/C/1=N\C(=O)C |
| 998 | S\1\C(=C/c2cc(OC)c(OCc3ccc(cc3)C)cc2)\C(=O)N(/C/1=N/c1ccccc1)c1ccccc1 |
| 999 | Clc1cc(F)c(F)cc1C(Oc1ccc(cc1OC)\C=C/1\C(=O)N(C)C(=O)N(C)C\1=O)=O |
| 1000 | Brc1cc(C)c(Cl)cc1Nc1nc(nc(n1)N\N=C/c1ccccc1OC)N1CCOCC1 |
| 1001 | Brc1c(COC)c(C#N)c(OCC(=O)N\N=C\c2cc(Br)ccc2)nc1C |
| 1002 | Brc1c(COC)c(C#N)c(OCC(=O)N\N=C\c2cc3OCOc3cc2[N+](=O)[O-])nc1C |
| 1003 | Brc1c(COC)c(C#N)c(OCC(=O)N\N=C\c2cc(n(c2-c2ccccc2)-c2ccc(Br)cc2)-c2ccccc2)nc1C |
| 1004 | O(c1ccc([N+](=O)[O-])cc1[N+](=O)[O-])c1ccc(cc1)\C=N\NC(=O)COc1c2ncccc2ccc1 |
| 1005 | S1\C(=C/c2sccc2)\C(=O)N(C2CCCCC2)C1=S |
| 1006 | Clc1ccccc1COc1ccccc1\C=C\1/SC(=S)NC/1=O |
| 1007 | O1CCN(CC1)c1nc(nc(n1)N\N=C\c1cc(Oc2ccc([N+](=O)[O-])cc2[N+](=O)[O-])c(OC)cc1)N1CCOCC1 |
| 1008 | Clc1ccccc1COc1ccccc1\C=C\1/SC(=O)N(C/1=O)c1ccccc1 |
| 1009 | S1\C(=C/c2cc(OC)c(OC(=O)C34CC5CC(C3)CC(C4)C5)cc2)\C(=O)N(c2ccc(OC)cc2)C1=S |
| 1010 | Clc1c(cccc1Cl)C(=O)Nc1cc2nc(oc2cc1)-c1ccc(cc1)C(C)(C)C |
| 1011 | Fc1cc(ccc1)C(=O)Nc1cc2nc(oc2cc1)-c1ccc(N(C)C)cc1 |
| 1012 | Brc1cc(C(=O)Nc2cc3nc(oc3cc2)-c2ccc(N(C)C)cc2)c(Cl)cc1 |
| 1013 | Brc1cc(C(=O)Nc2cc(-c3oc4c(n3)cc(cc4)C)c(O)cc2)c(Cl)cc1 |
| 1014 | Ic1ccc(I)cc1C(=O)Nc1ccc(cc1)-c1sc2c(n1)cccc2 |
| 1015 | Brc1ccccc1-c1oc2c(n1)cc(NC(=O)c1cc([N+](=O)[O-])ccc1Cl)cc2 |
| 1016 | s1c2c(nc1-c1cc(NC(=O)c3cc4c(cc3)cccc4)c(cc1)C)cccc2 |
| 1017 | O(C)c1c(cccc1OC)\C=C/1\C(=NN(C\1=O)c1ccccc1)C |
| 1018 | Clc1cccc(Cl)c1\C=C\1/SC(=S)N(NC(=O)c2ccncc2)C/1=O |
| 1019 | Clc1ccc(cc1)\C=C\1/N=C(OC/1=O)c1ccccc1C |
| 1020 | Brc1ccc(cc1)\C=C\1/N=C(OC/1=O)c1cc(C)c(cc1)C |
| 1021 | Clc1c(cccc1Cl)\C=C\1/N=C(OC/1=O)c1cc(ccc1)C |
| 1022 | O1C(=N\C(=C/c2cc3OCOc3cc2)\C1=O)c1ccc(OCCCC)cc1 |
| 1023 | Clc1c(NC(=O)c2nn3c(N=C(C=C3C(F)(F)F)c3sccc3)c2Cl)cccc1Cl |
| 1024 | s1c2c(nc1NC(=O)c1ccccc1Oc1ccccc1)c(OC)ccc2 |
| 1025 | s1cc(nc1NC(=O)c1ccc(S(=O)(=O)N(CCOC)CCOC)cc1)-c1ccc(cc1)C |
| 1026 | S(C)C=1NC(=O)CC(C=1C#N)c1ccc(OC)cc1 |
| 1027 | O1c2c(C=C(C(=O)Nc3ccccc3)C1=O)c1c(cc2)cccc1 |
| 1028 | Brc1ccc(NC(=O)CSC=2NC(=O)CC(C=2C#N)c2ccc(Cl)cc2)cc1 |
| 1029 | O1c2c(cc(CCC)c(O)c2)C(=O)C(c2cc3OCCOc3cc2)=C1C |
| 1030 | s1cc(nc1C)C1=COc2c(C1=O)c(O)cc(c2)C |
| 1031 | o1c2c(cc1C1=COc3c(ccc(OC(=O)C)c3)C1=O)cccc2 |
| 1032 | o1c2c(cc1C1=COc3c(ccc(O)c3C)C1=O)cccc2 |
| 1033 | o1c2c(cc1C1=COc3c(cc(CCC)c(OC(=O)C)c3)C1=O)cccc2 |
| 1034 | s1cc(nc1-c1ccccc1)C1=COc2c(cc(CCCCCC)c(O)c2)C1=O |
| 1035 | S1\C(=C/c2cc(OC)c(OC)cc2)\C(=O)N(c2ccc(OCC)cc2)C1=S |
| 1036 | S1\C(=C/c2ccccc2)\C(=O)N(CC(=O)Nc2sc(cn2)Cc2cc(ccc2)C)C1=S |
| 1037 | S1\C(=C/c2ccc([N+](=O)[O-])cc2)\C(=O)N(NC(=O)CCCCCCC)C1=S |
| 1038 | O=C1NC(C(C(OC\C=C\C)=O)=C(N1)C)c1c2c(ccc1)cccc2 |
| 1039 | Clc1cc(ccc1)C1C2=C(NC(C)=C1C(OCCOC)=O)CCCC2=O |
| 1040 | s1cccc1C1C2=C(NC(C)=C1C(OCCOc1ccccc1)=O)CCCC2=O |
